# Supplementary figures and images for: Gαq-PKD/PKCμ signal regulating the nuclear export of HDAC5 to induce the IκB expression and limit the NF-κB-mediated inflammatory response essential for early pregnancy
Source: eLife. 2023 Jul 27;12:e83083. doi: 10.7554/eLife.83083 (PMC10374280; doi:10.7554/eLife.83083)

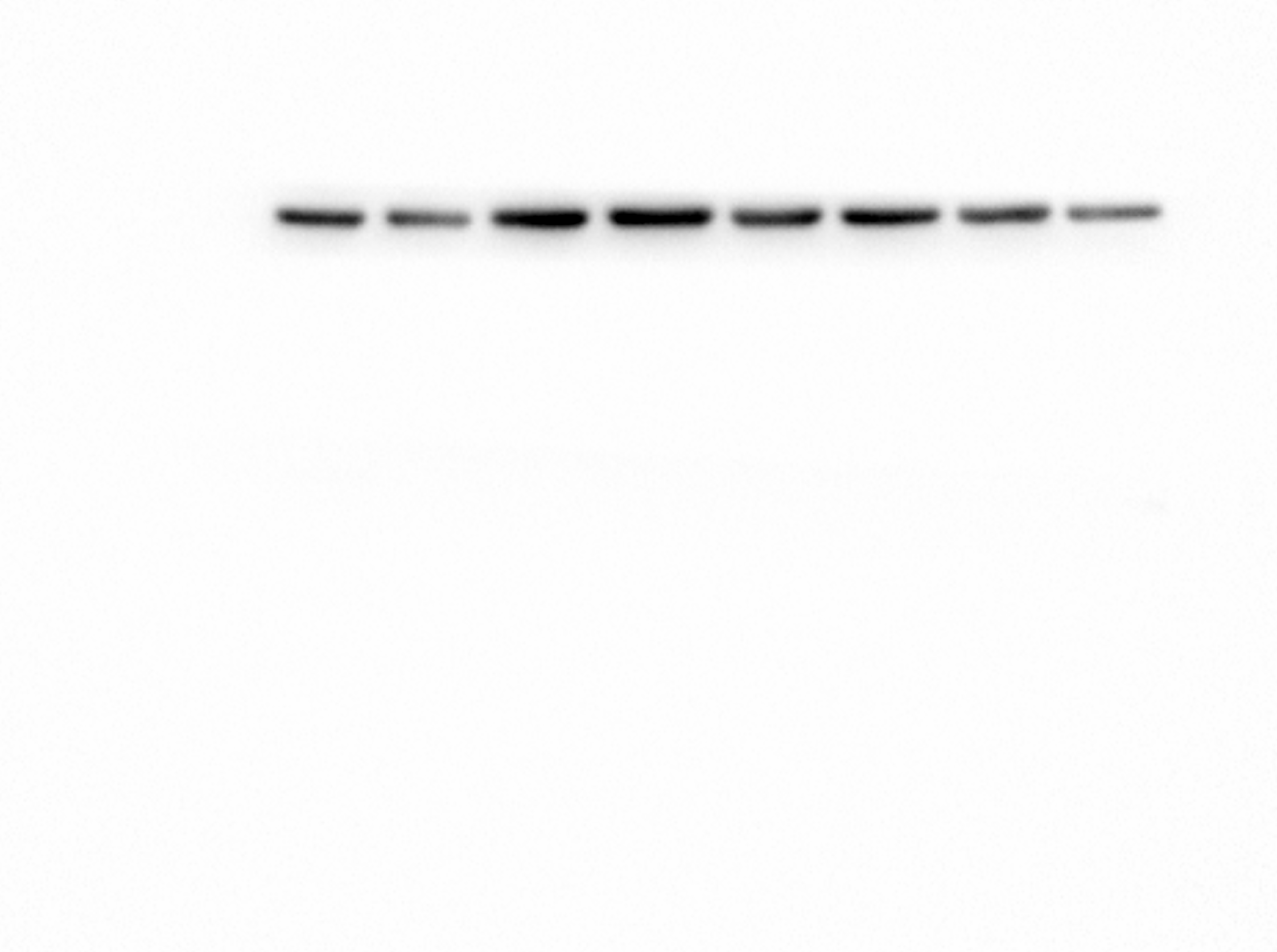

Supplement: Figure 1—source data 1. [file elife-83083-fig1-data1.zip › Figure 1-source data/Figure 1D GAPDH.tif]

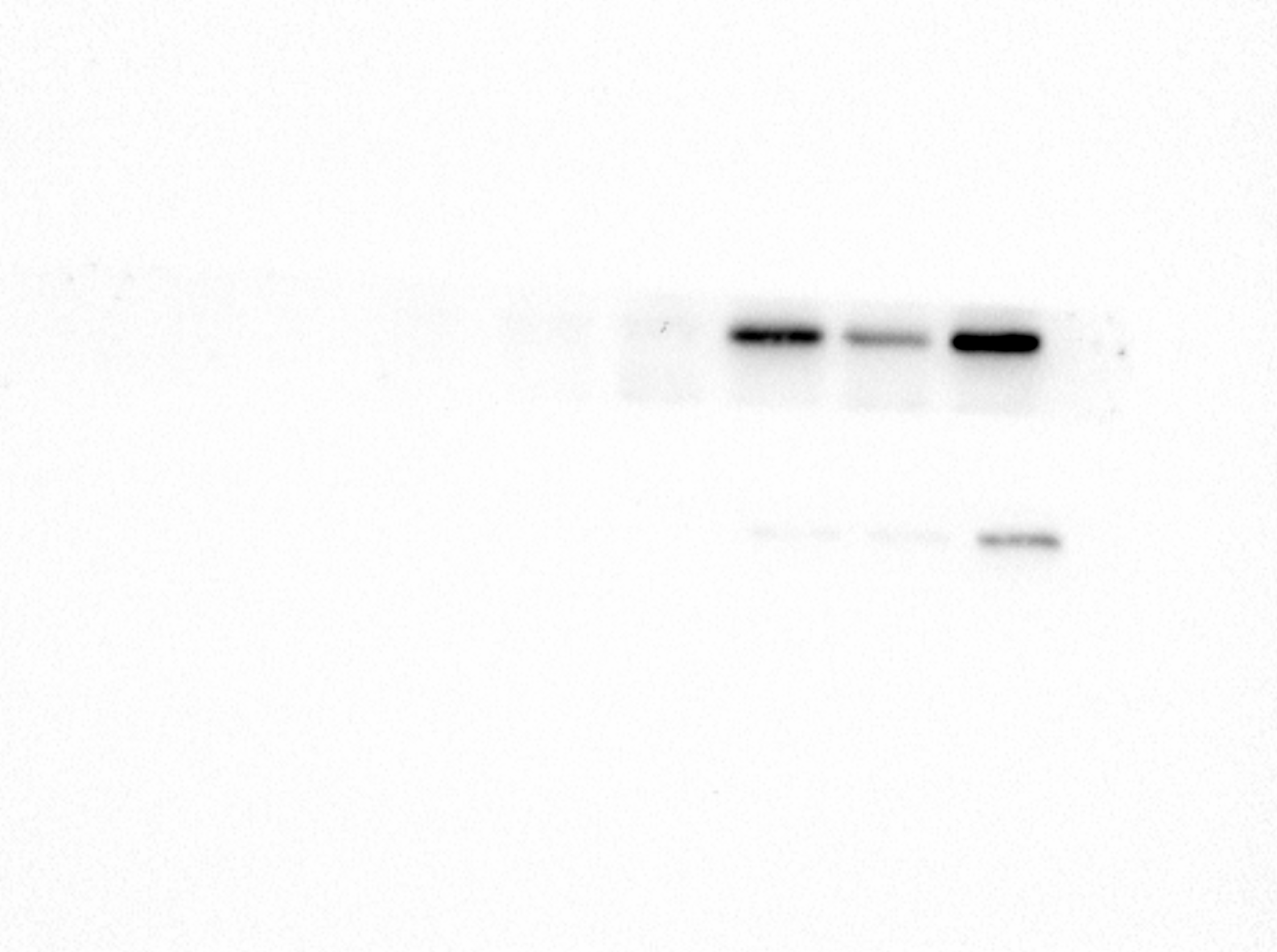

Supplement: Figure 1—source data 1. [file elife-83083-fig1-data1.zip › Figure 1-source data/Figure 1D IGFBP-1.tif]

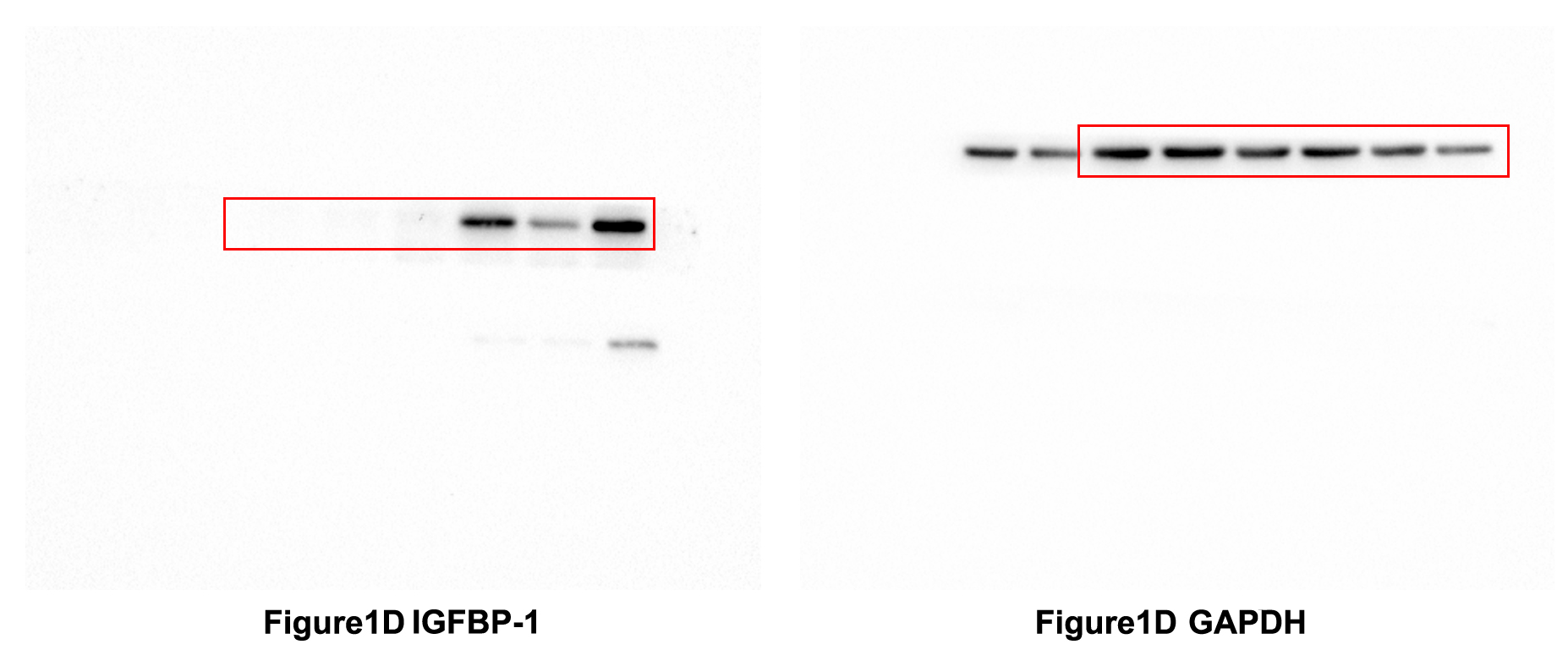

Supplement: Figure 1—source data 1. [file elife-83083-fig1-data1.zip › Figure 1-source data/Figure 1-source data.jpg]

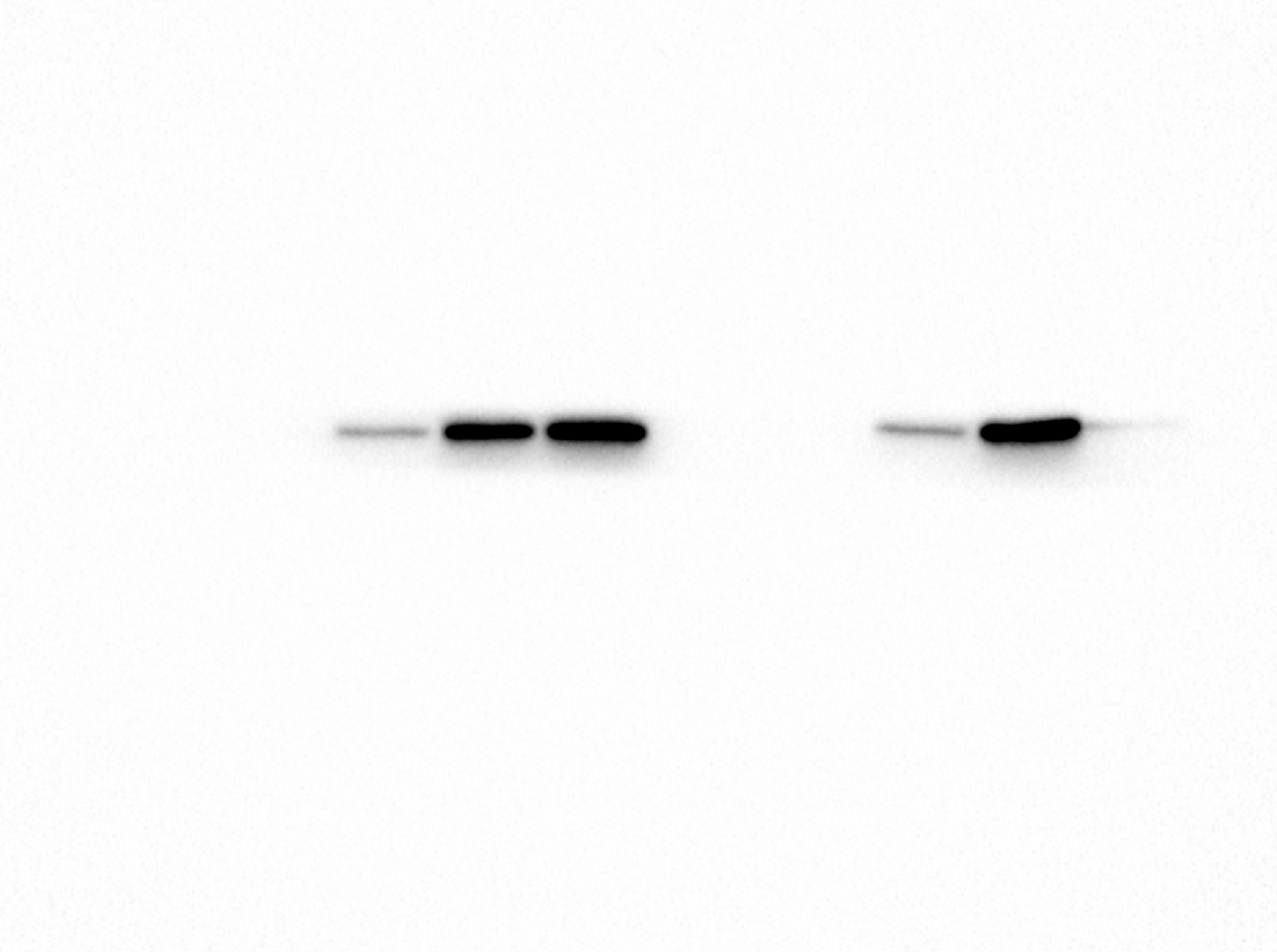

Supplement: Figure 1—figure supplement 1—source data 1. [file elife-83083-fig1-figsupp1-data1.zip › Figure 1-figure supplement 1-source data/Figure 1-figure supplement 1C IGFBP-1.tif]

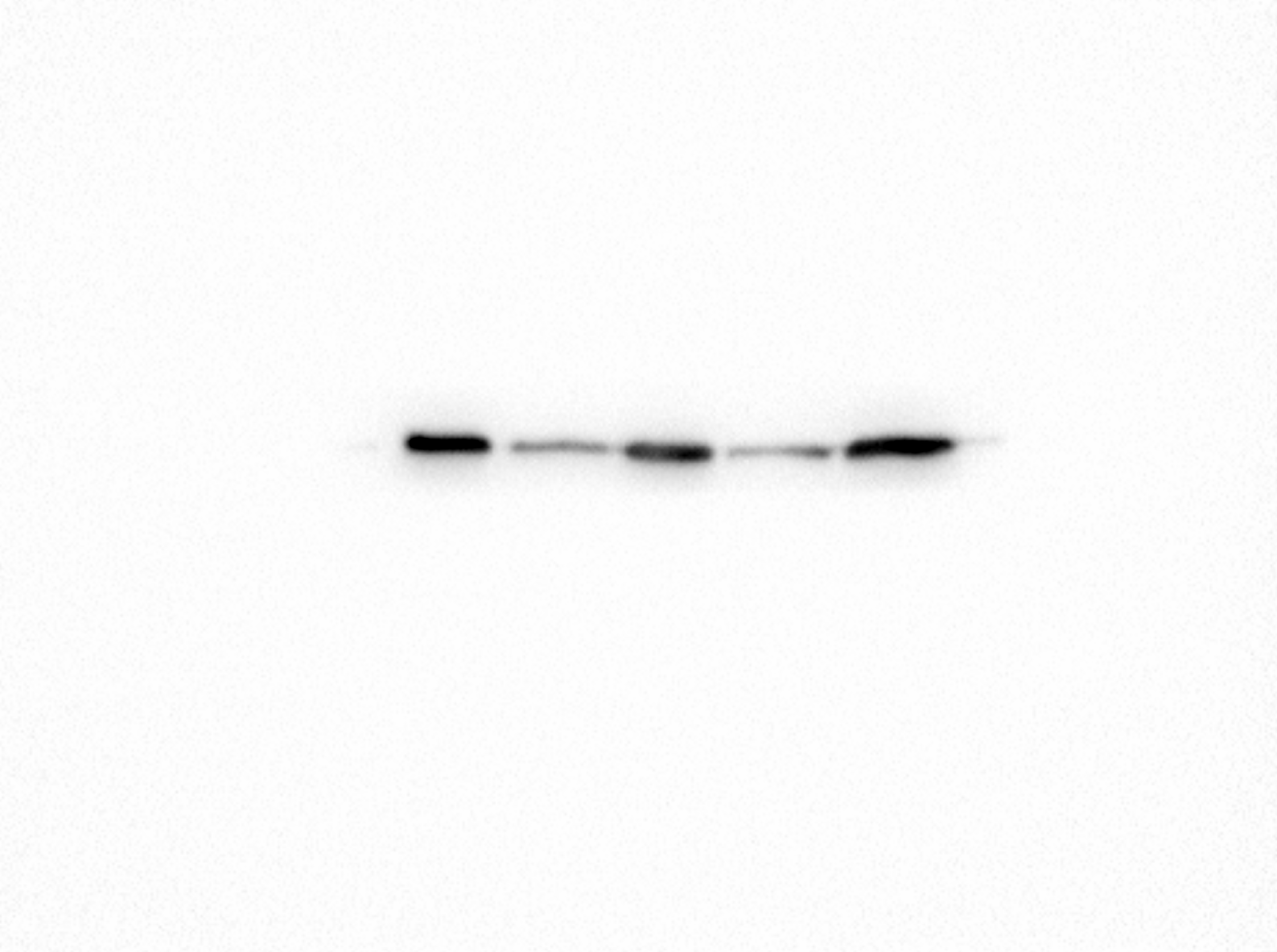

Supplement: Figure 1—figure supplement 1—source data 1. [file elife-83083-fig1-figsupp1-data1.zip › Figure 1-figure supplement 1-source data/Figure 1-figure supplement 1E GAPDH.tif]

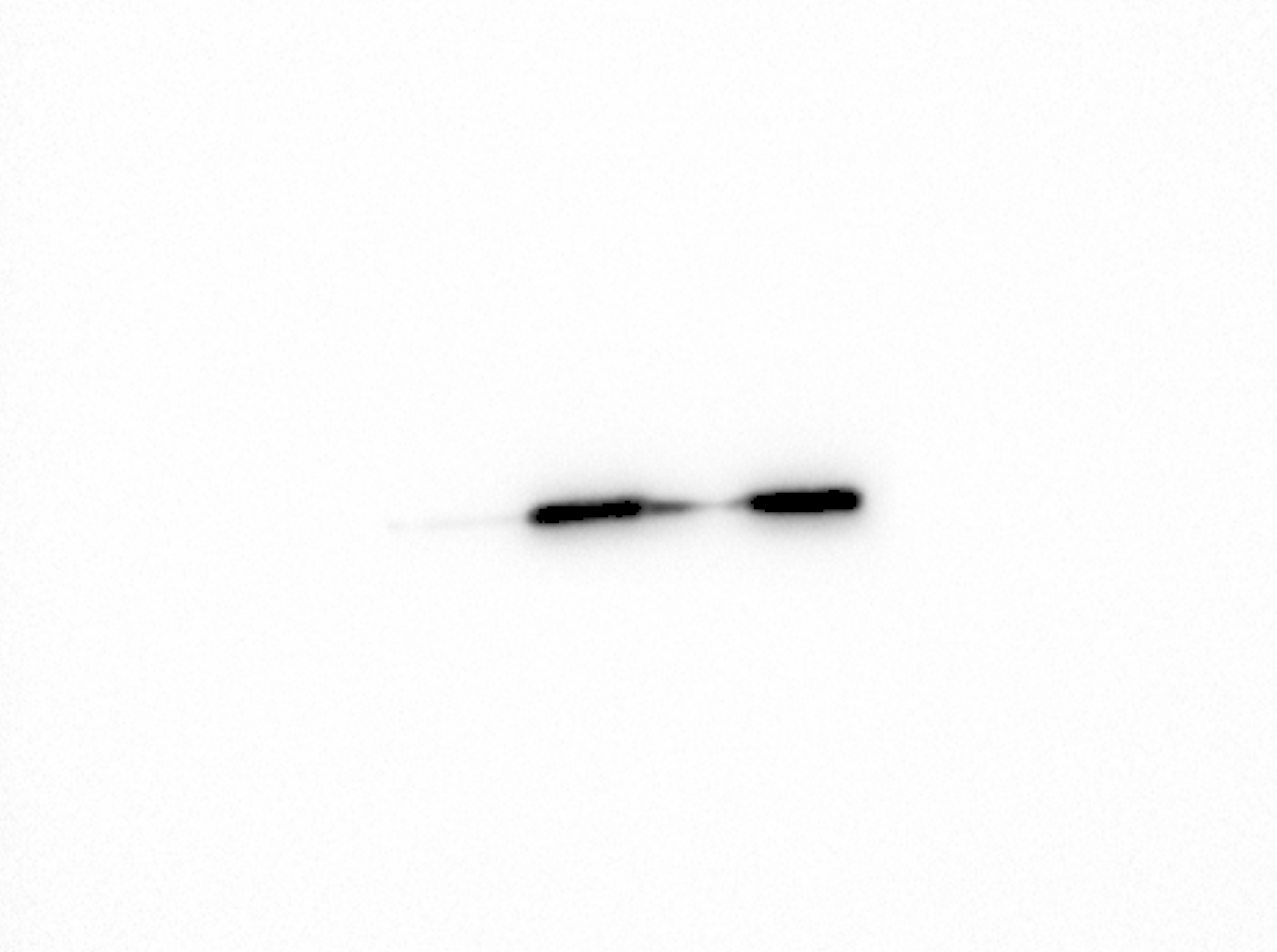

Supplement: Figure 1—figure supplement 1—source data 1. [file elife-83083-fig1-figsupp1-data1.zip › Figure 1-figure supplement 1-source data/Figure 1-figure supplement 1E H3.tif]

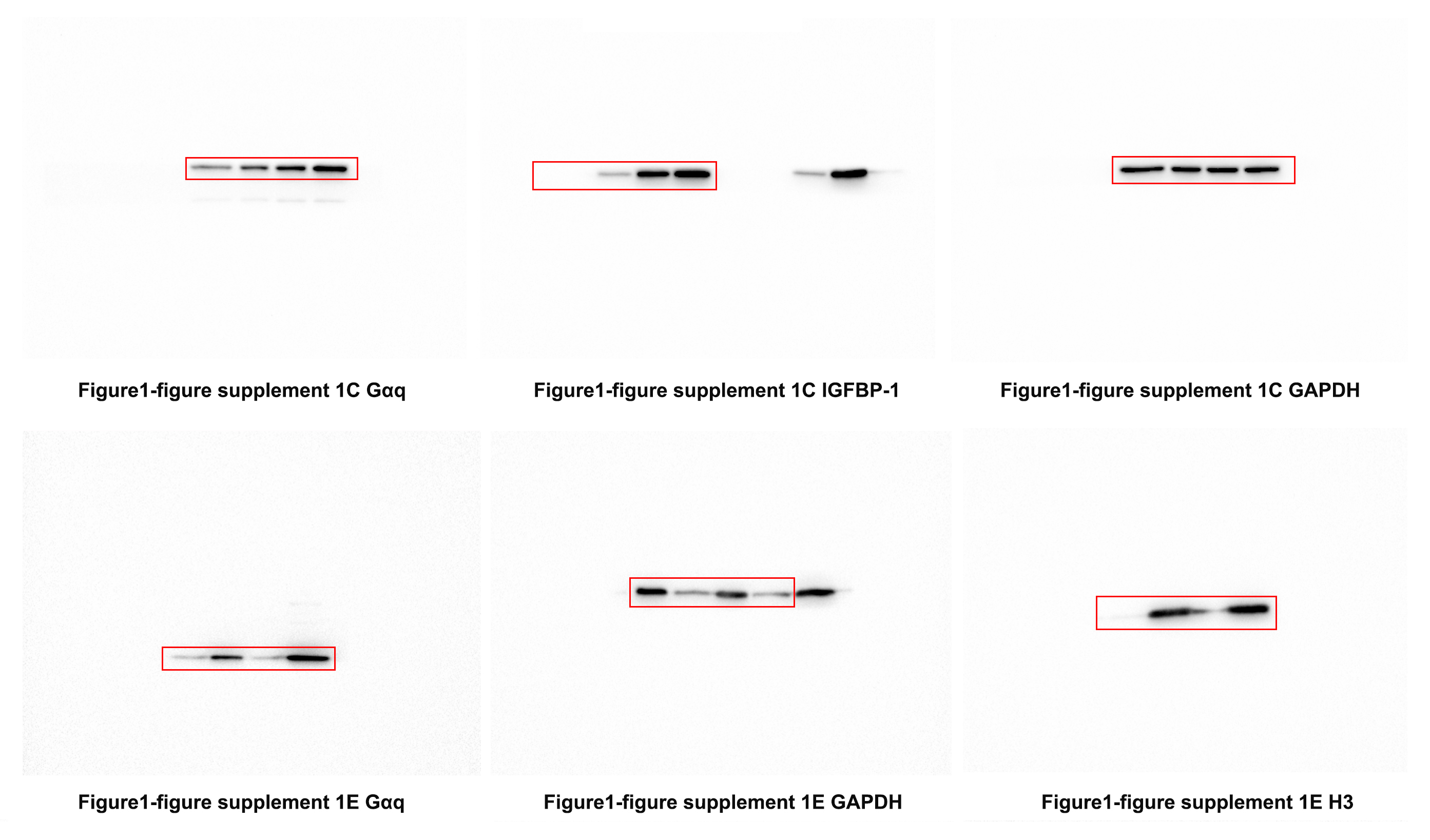

Supplement: Figure 1—figure supplement 1—source data 1. [file elife-83083-fig1-figsupp1-data1.zip › Figure 1-figure supplement 1-source data/Figure 1-figure supplement 1-source data.jpg]

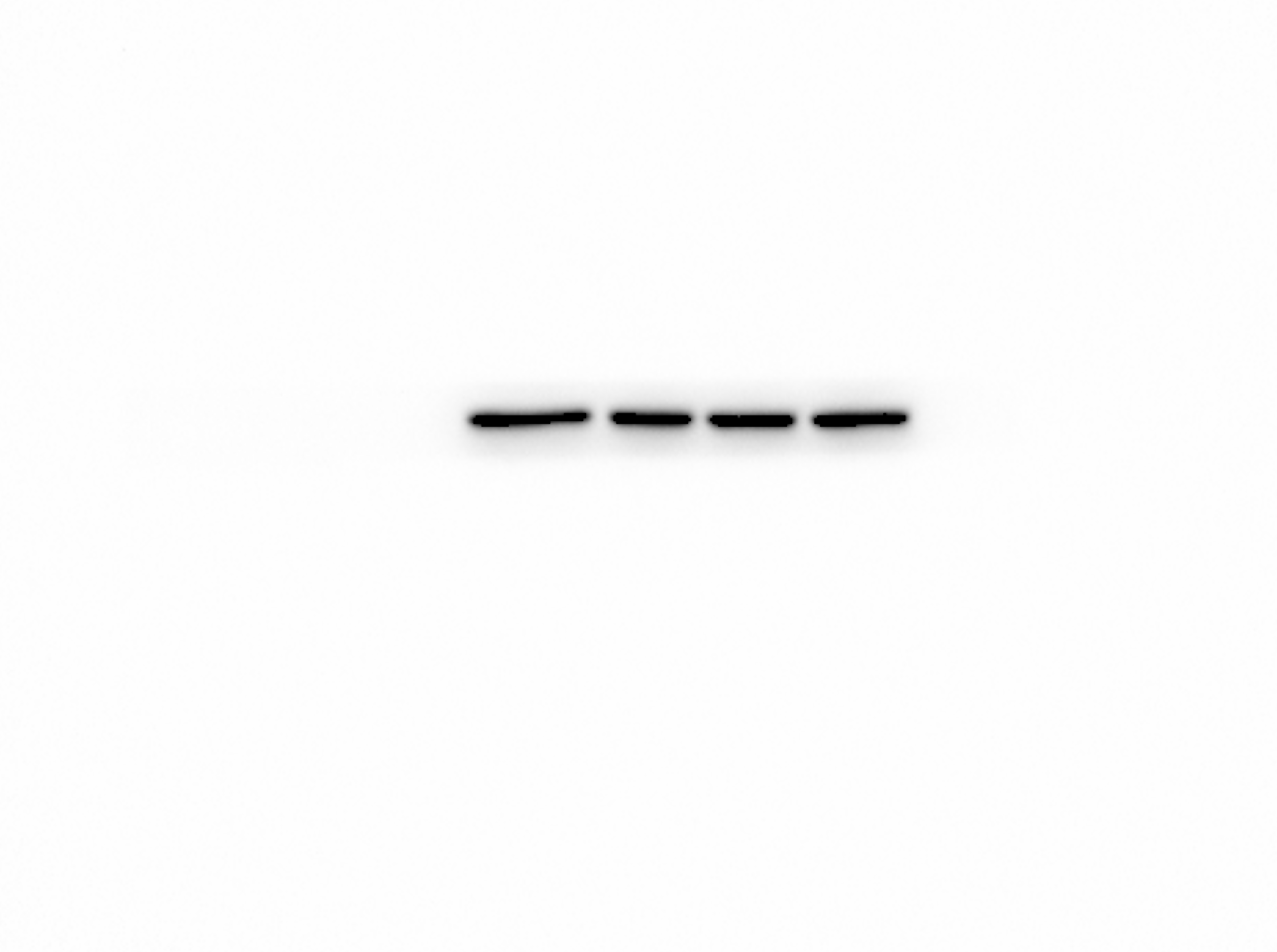

Supplement: Figure 1—figure supplement 1—source data 1. [file elife-83083-fig1-figsupp1-data1.zip › Figure 1-figure supplement 1-source data/Figure 1-figure supplement 1C GAPDH.tif]

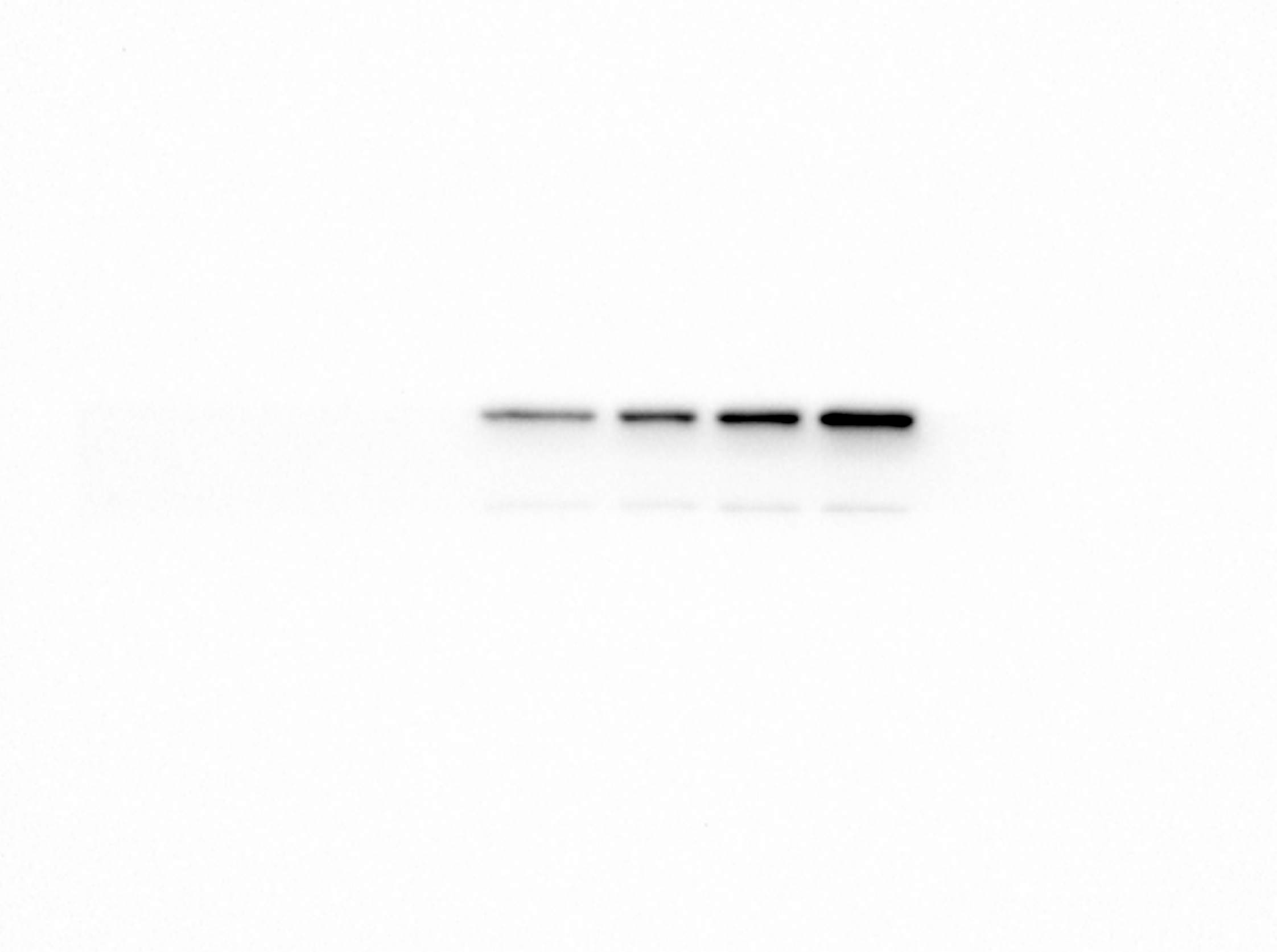

Supplement: Figure 1—figure supplement 1—source data 1. [file elife-83083-fig1-figsupp1-data1.zip › Figure 1-figure supplement 1-source data/Figure 1-figure supplement 1C Ga┴q.tif]

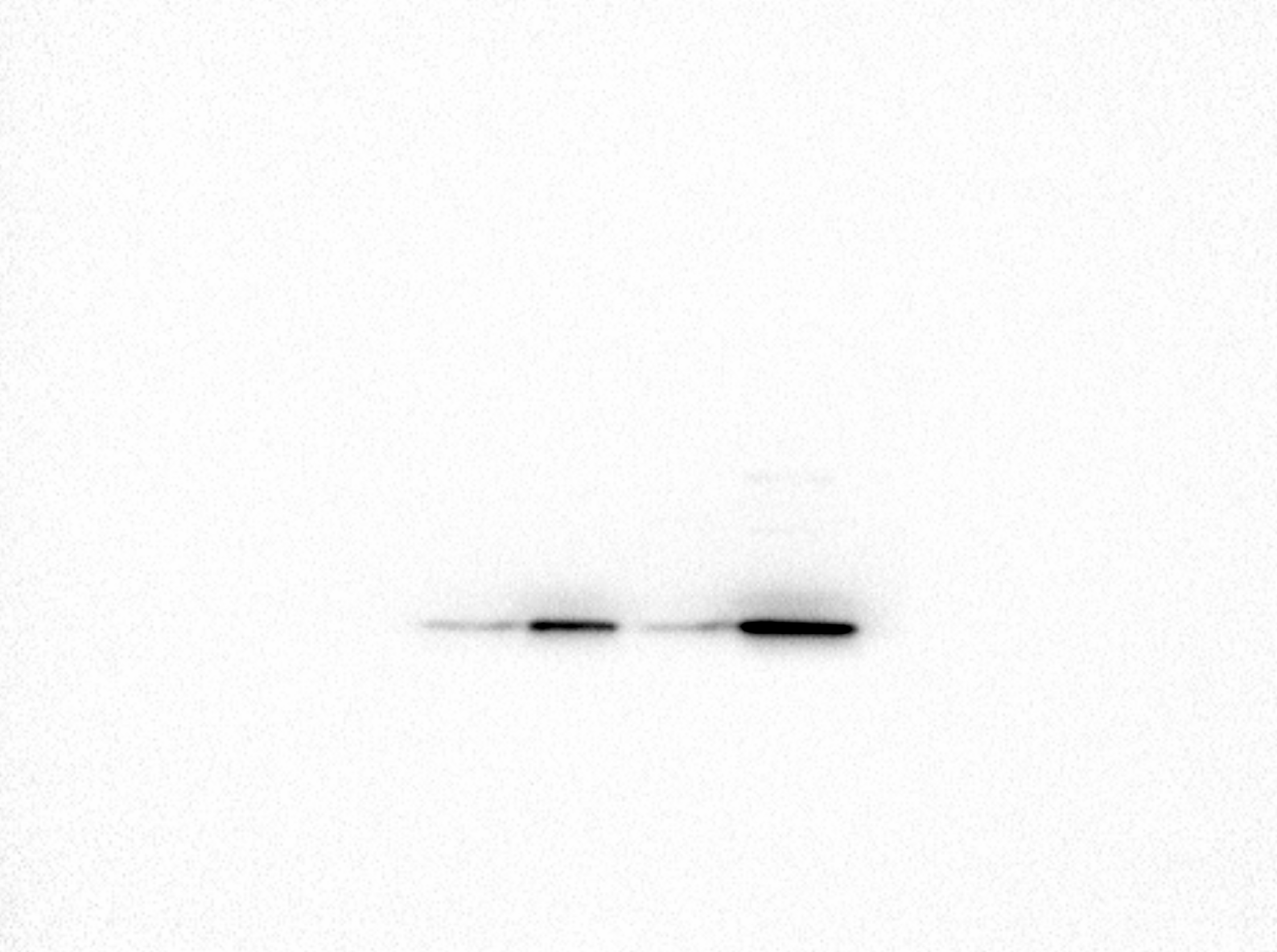

Supplement: Figure 1—figure supplement 1—source data 1. [file elife-83083-fig1-figsupp1-data1.zip › Figure 1-figure supplement 1-source data/Figure 1-figure supplement 1E Ga┴q.tif]

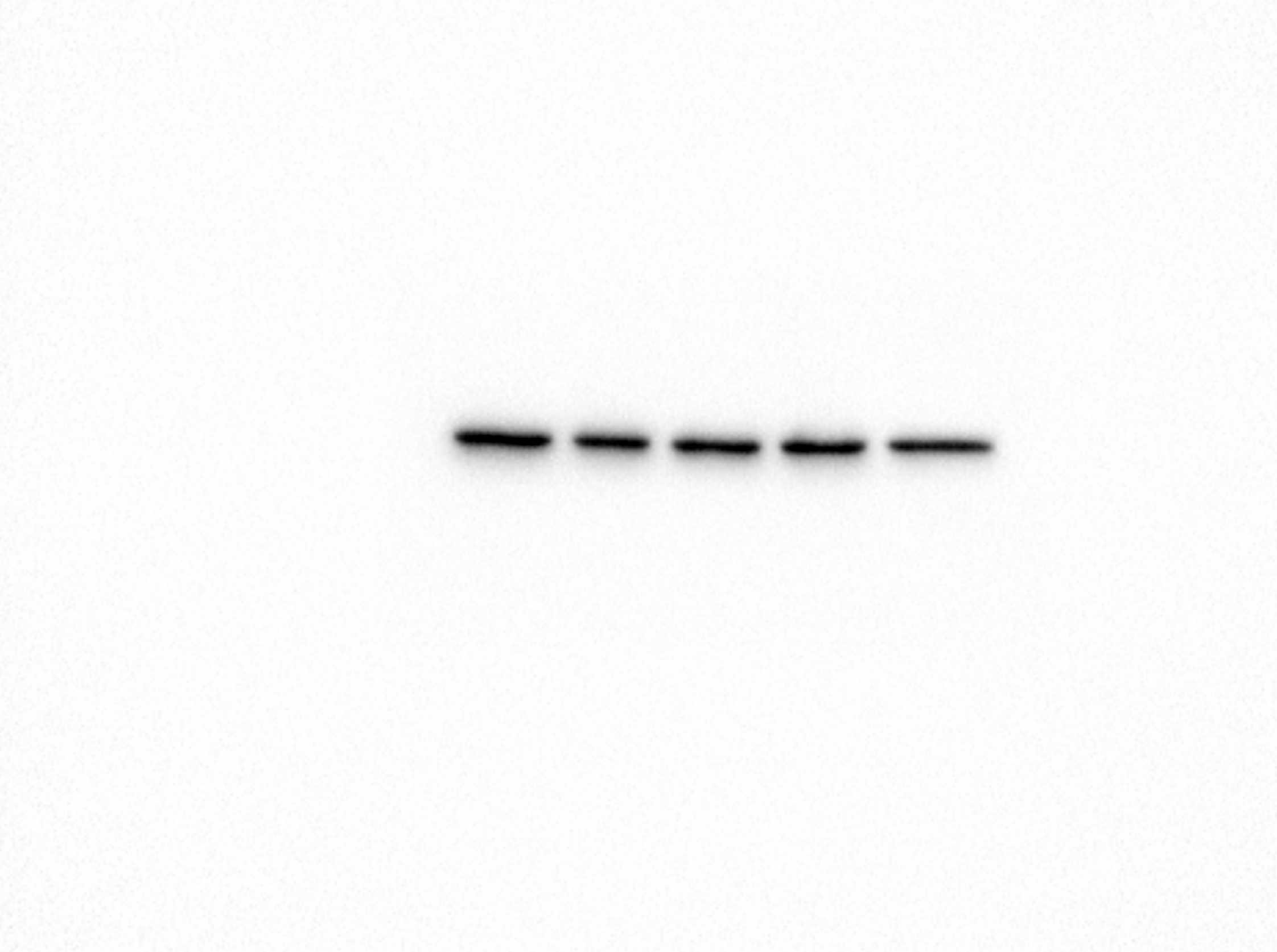

Supplement: Figure 1—figure supplement 2—source data 1. [file elife-83083-fig1-figsupp2-data1.zip › Figure 1-figure supplement 2-source data/Figure 1-Figure supplement 2B GAPDH.tif]

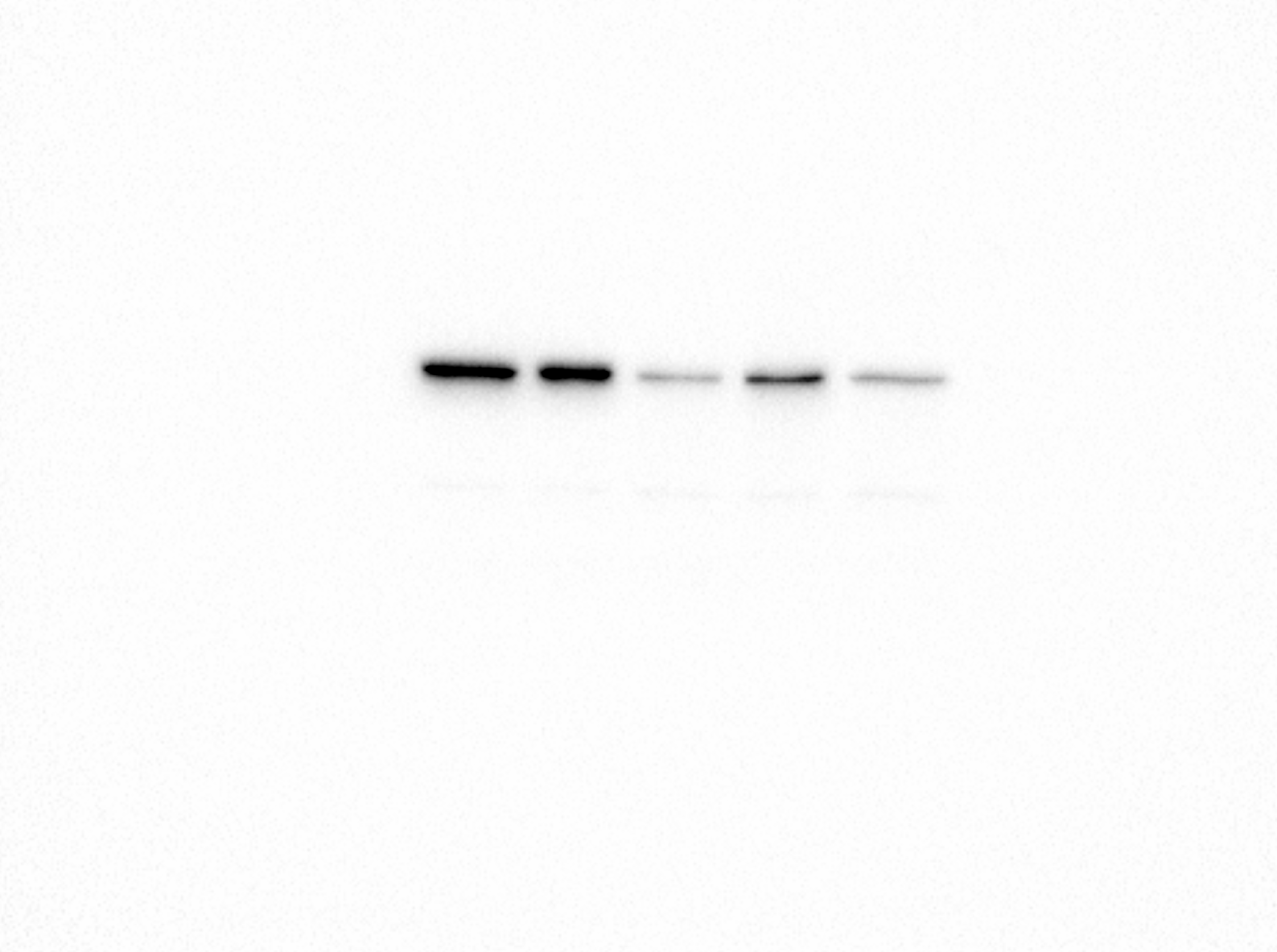

Supplement: Figure 1—figure supplement 2—source data 1. [file elife-83083-fig1-figsupp2-data1.zip › Figure 1-figure supplement 2-source data/Figure 1-Figure supplement 2B Ga┴q.tif]

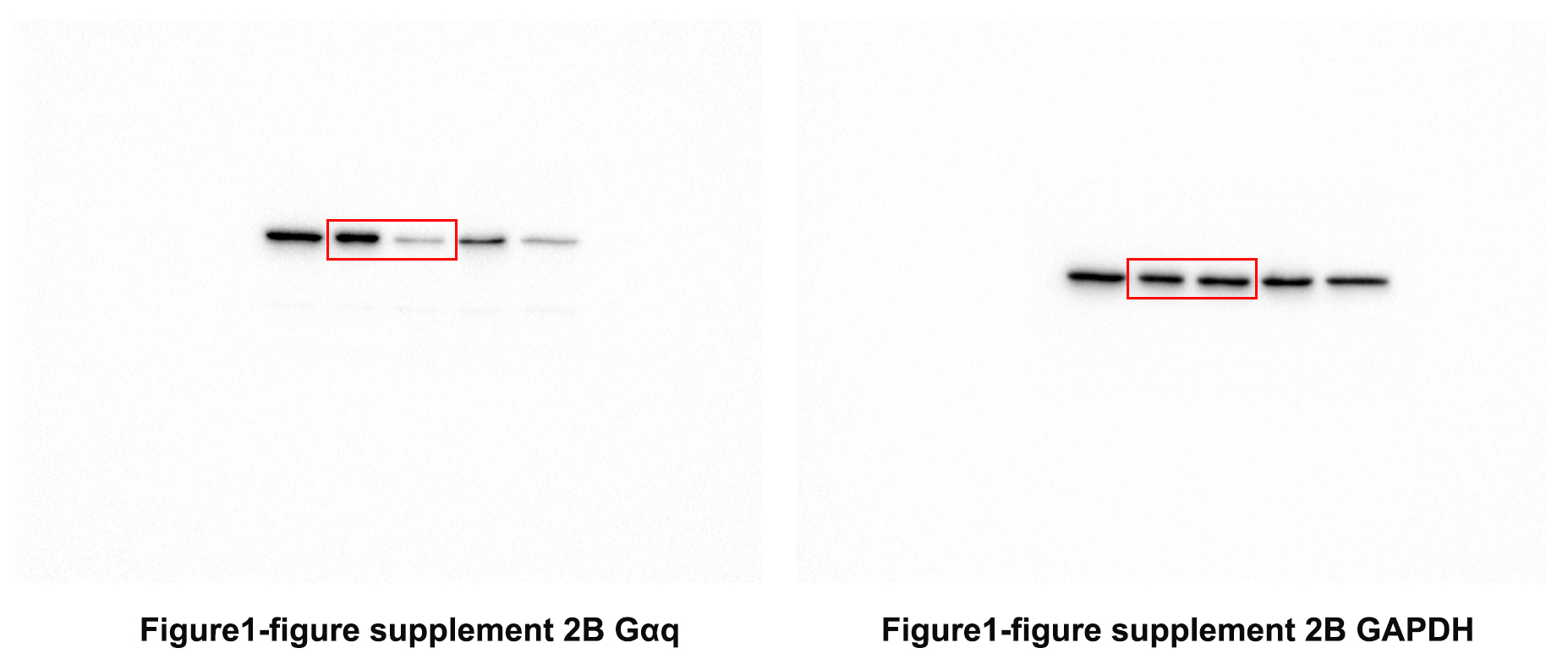

Supplement: Figure 1—figure supplement 2—source data 1. [file elife-83083-fig1-figsupp2-data1.zip › Figure 1-figure supplement 2-source data/Figure 1-figure supplement 2-source data.jpg]

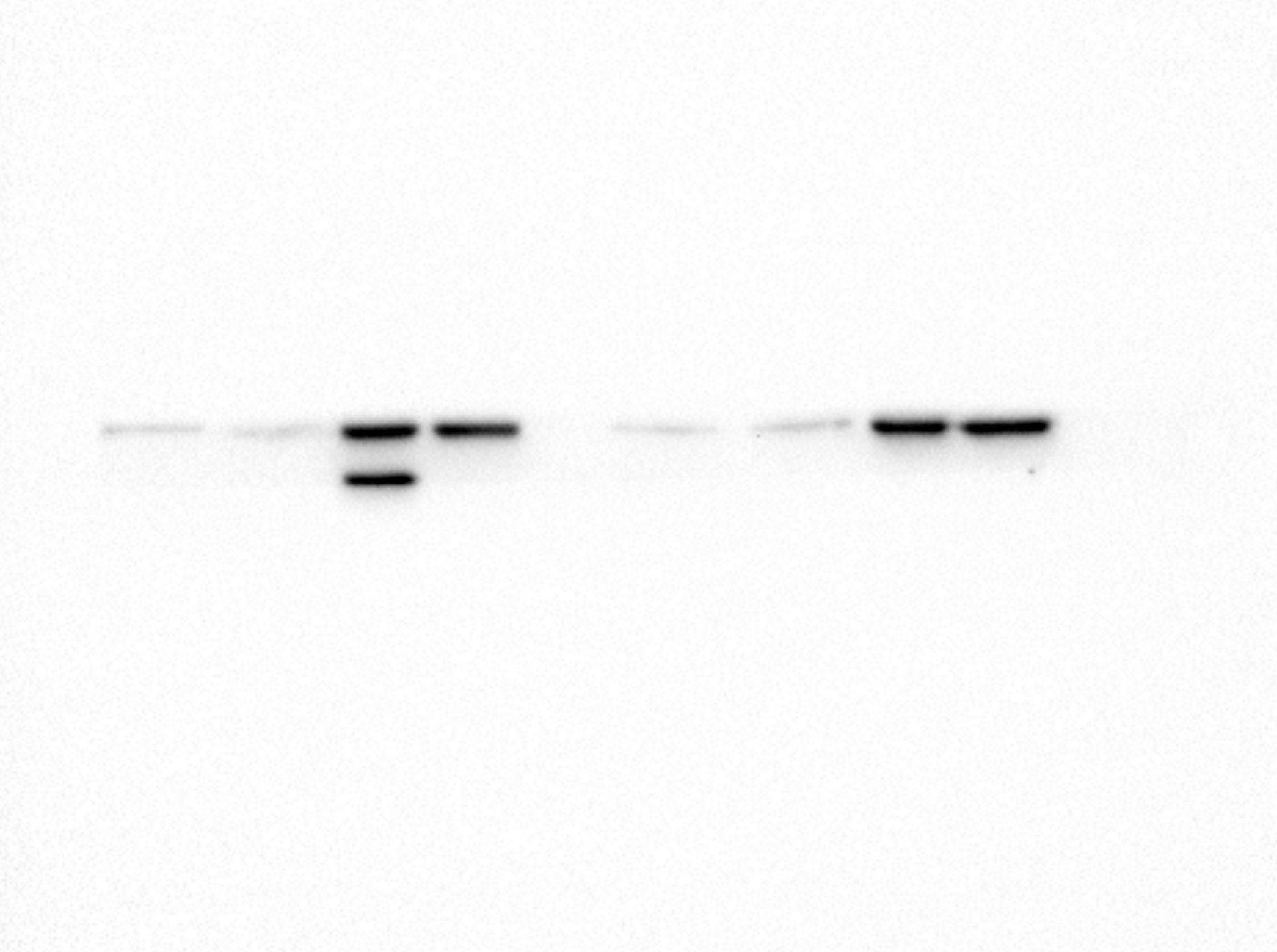

Supplement: Figure 2—source data 1. [file elife-83083-fig2-data1.zip › Figure 2-source data/Figure 2A GAPDH.tif]

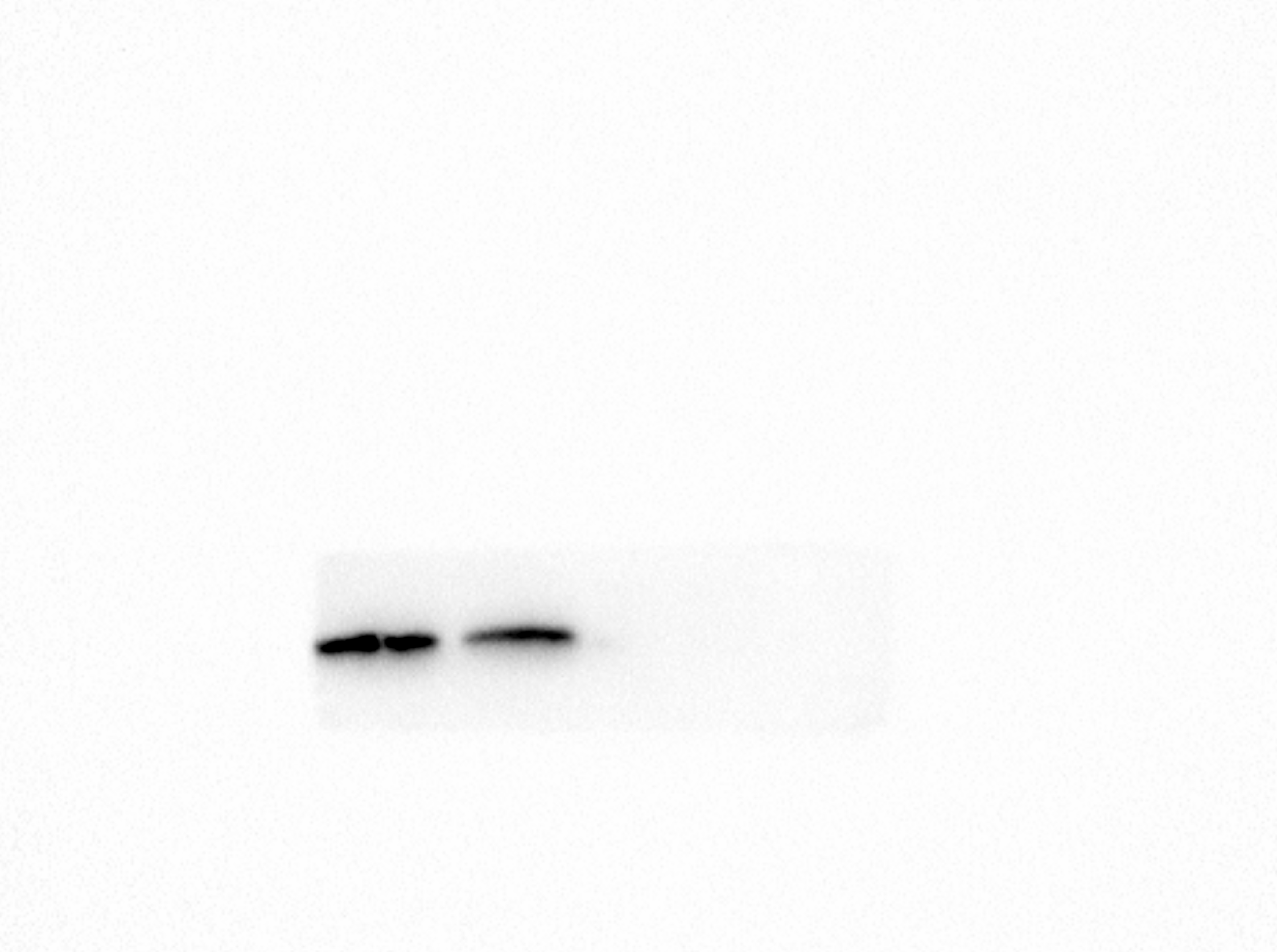

Supplement: Figure 2—source data 1. [file elife-83083-fig2-data1.zip › Figure 2-source data/Figure 2A H3.tif]

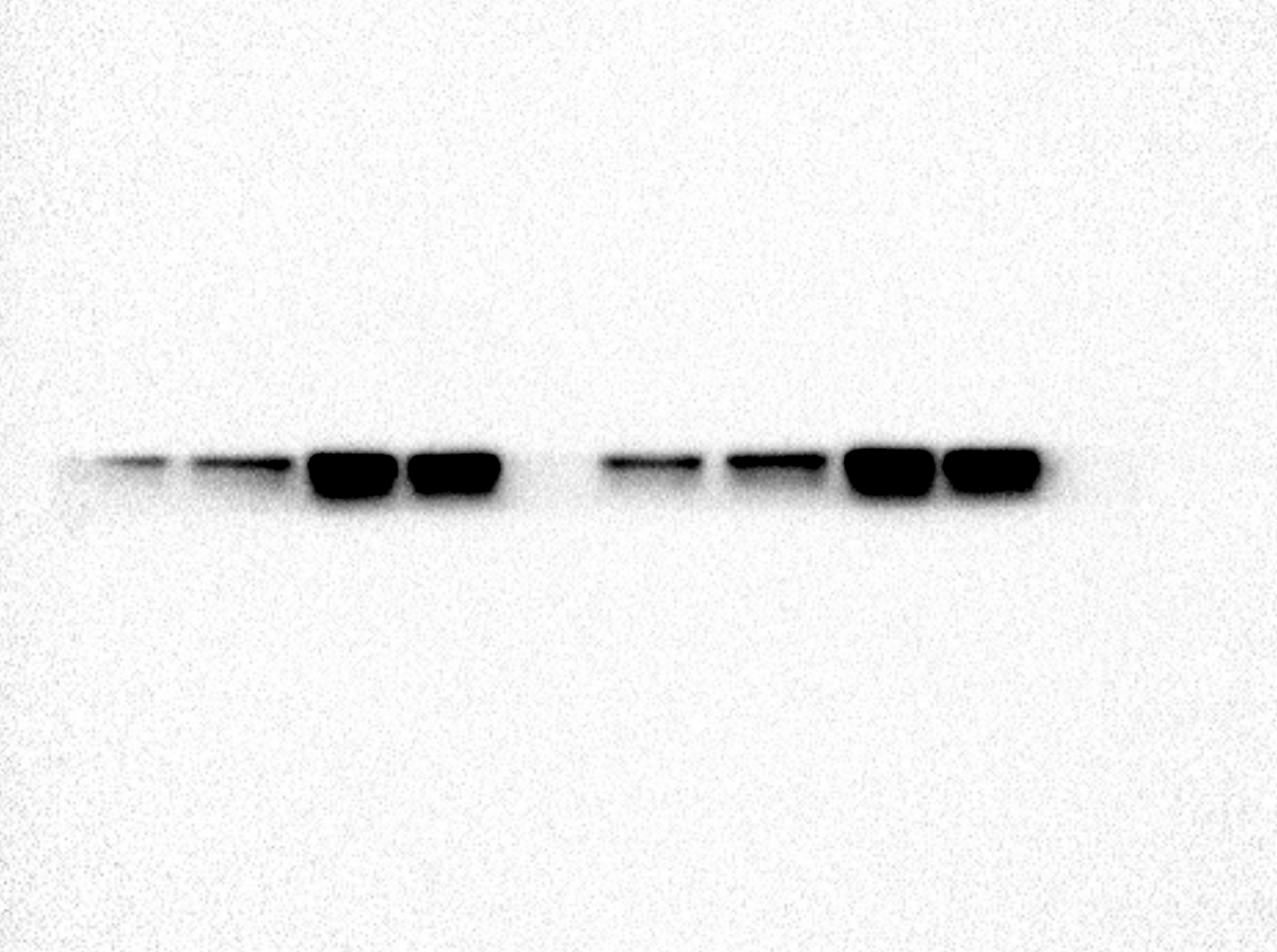

Supplement: Figure 2—source data 1. [file elife-83083-fig2-data1.zip › Figure 2-source data/Figure 2A p50.tif]

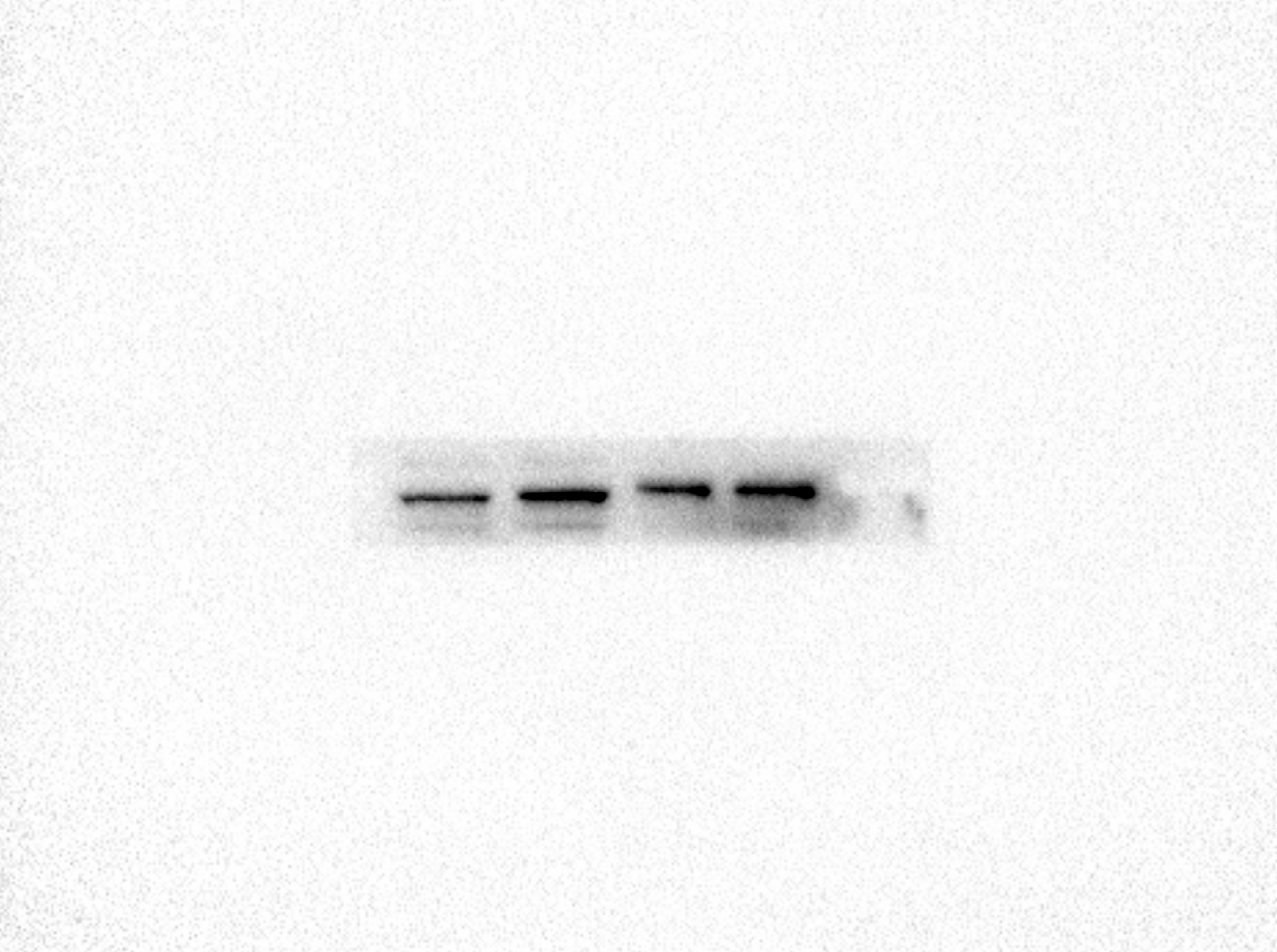

Supplement: Figure 2—source data 1. [file elife-83083-fig2-data1.zip › Figure 2-source data/Figure 2A p65.tif]

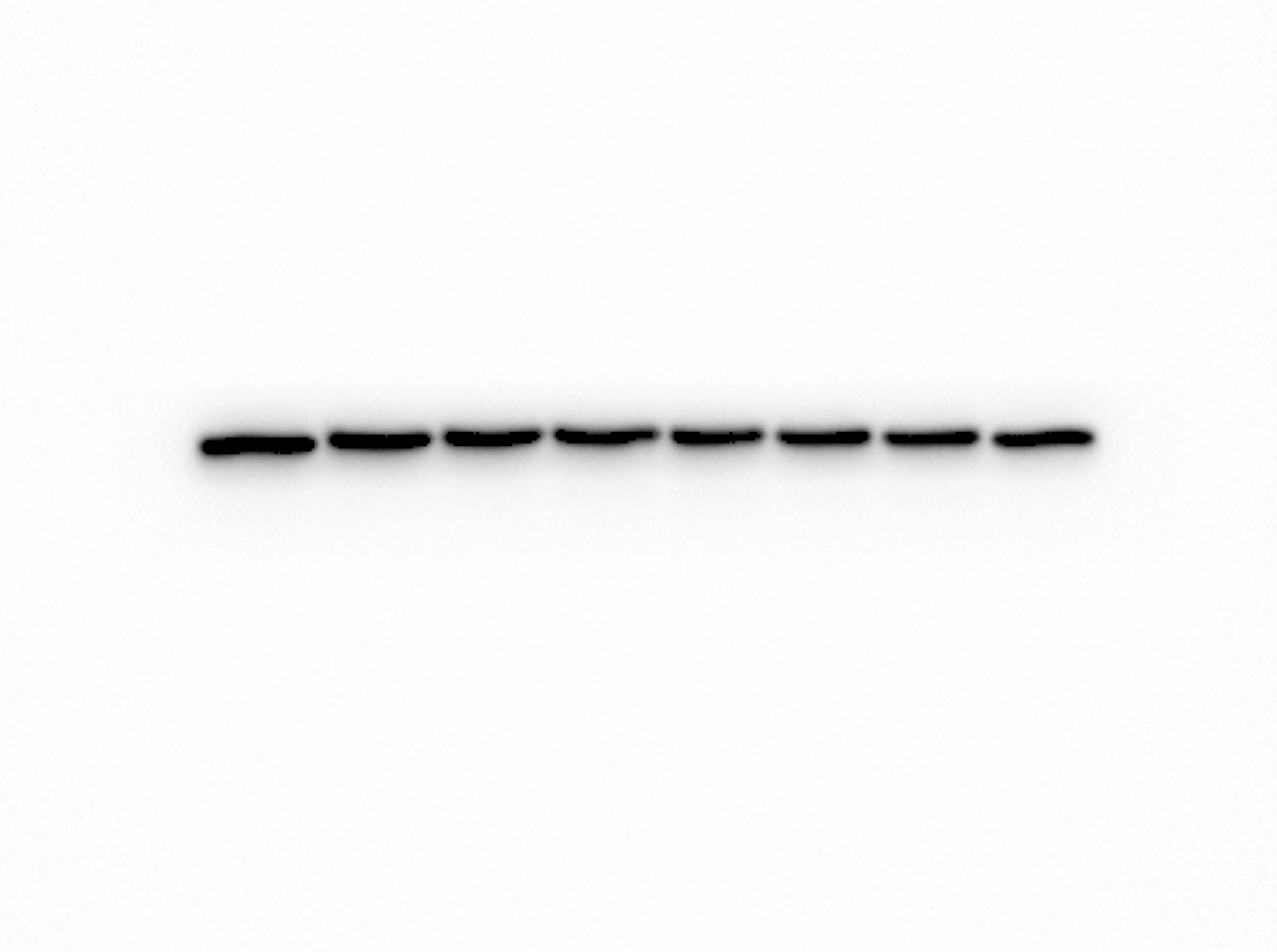

Supplement: Figure 2—source data 1. [file elife-83083-fig2-data1.zip › Figure 2-source data/Figure 2C GAPDH.tif]

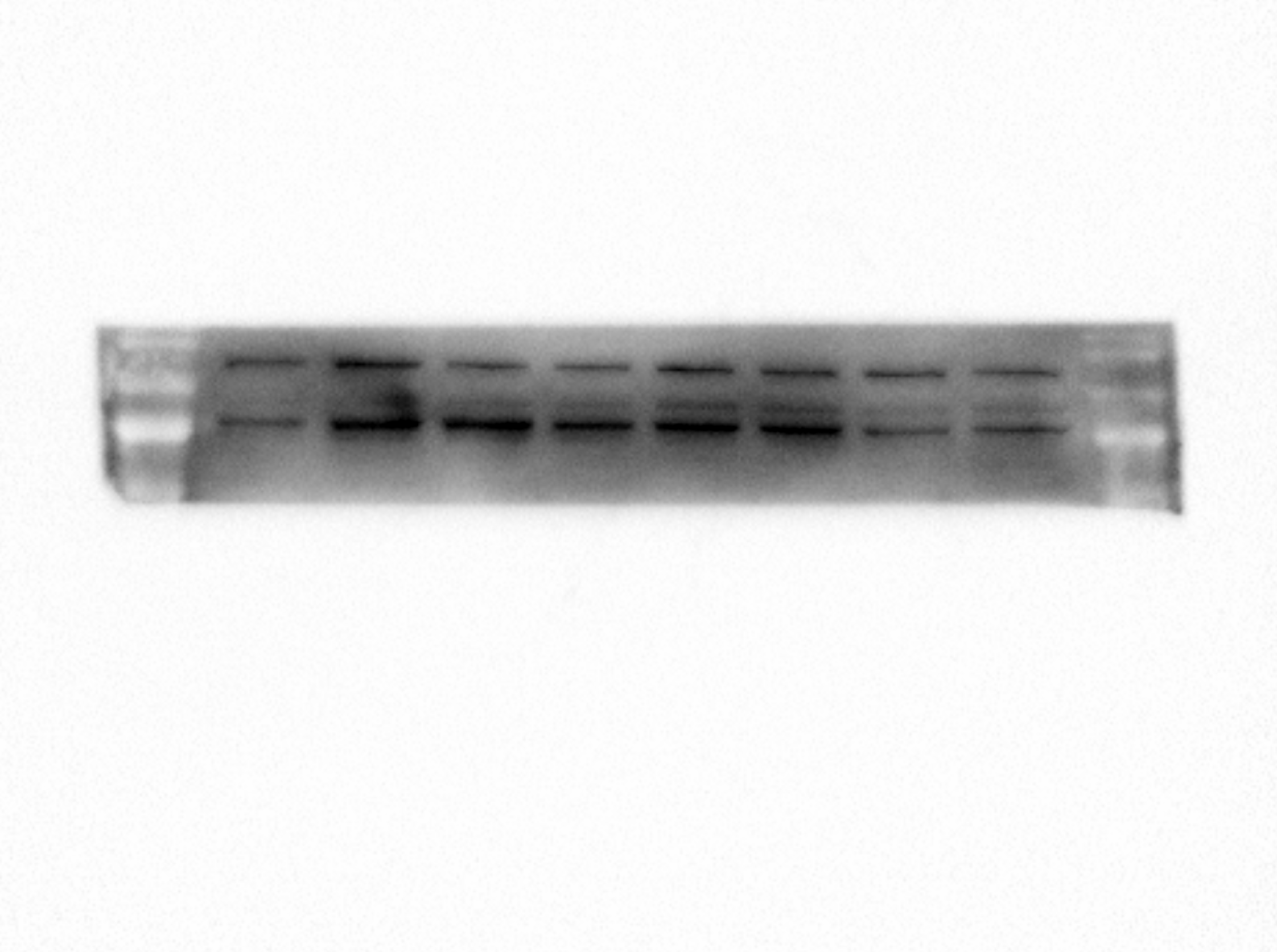

Supplement: Figure 2—source data 1. [file elife-83083-fig2-data1.zip › Figure 2-source data/Figure 2C p-p65.tif]

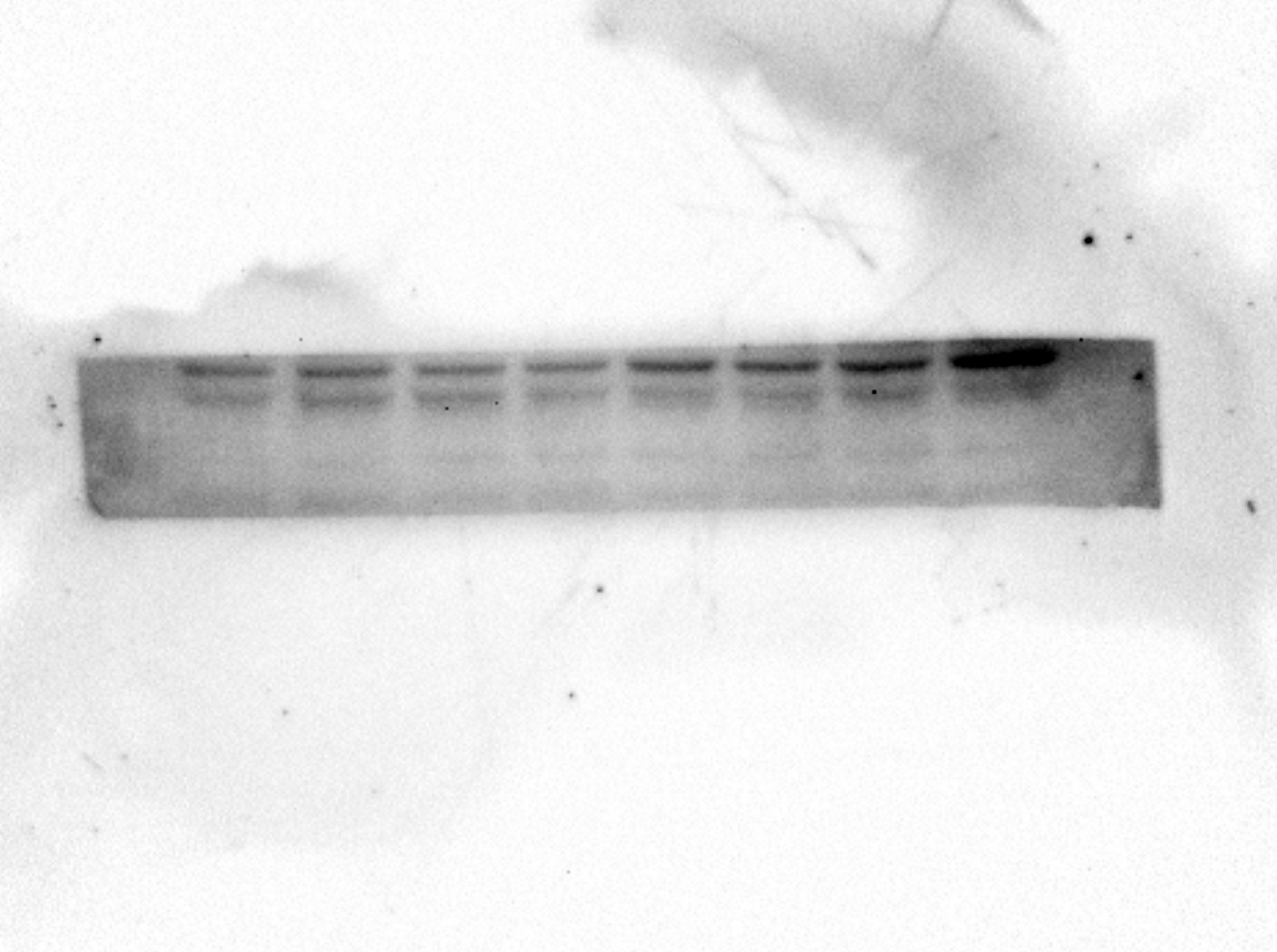

Supplement: Figure 2—source data 1. [file elife-83083-fig2-data1.zip › Figure 2-source data/Figure 2F IL-1a┬.tif]

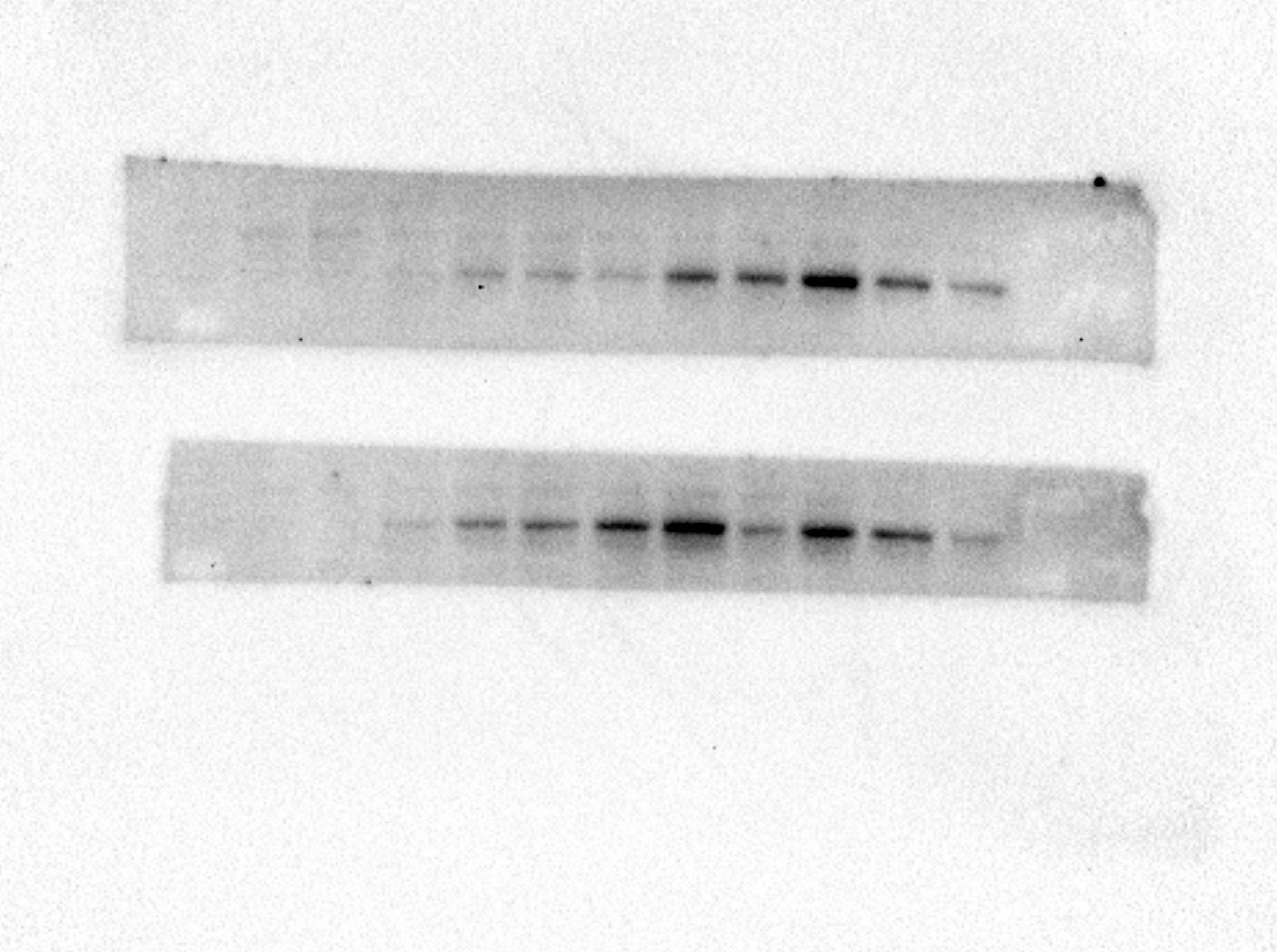

Supplement: Figure 2—source data 1. [file elife-83083-fig2-data1.zip › Figure 2-source data/Figure 2F p-STAT3.tif]

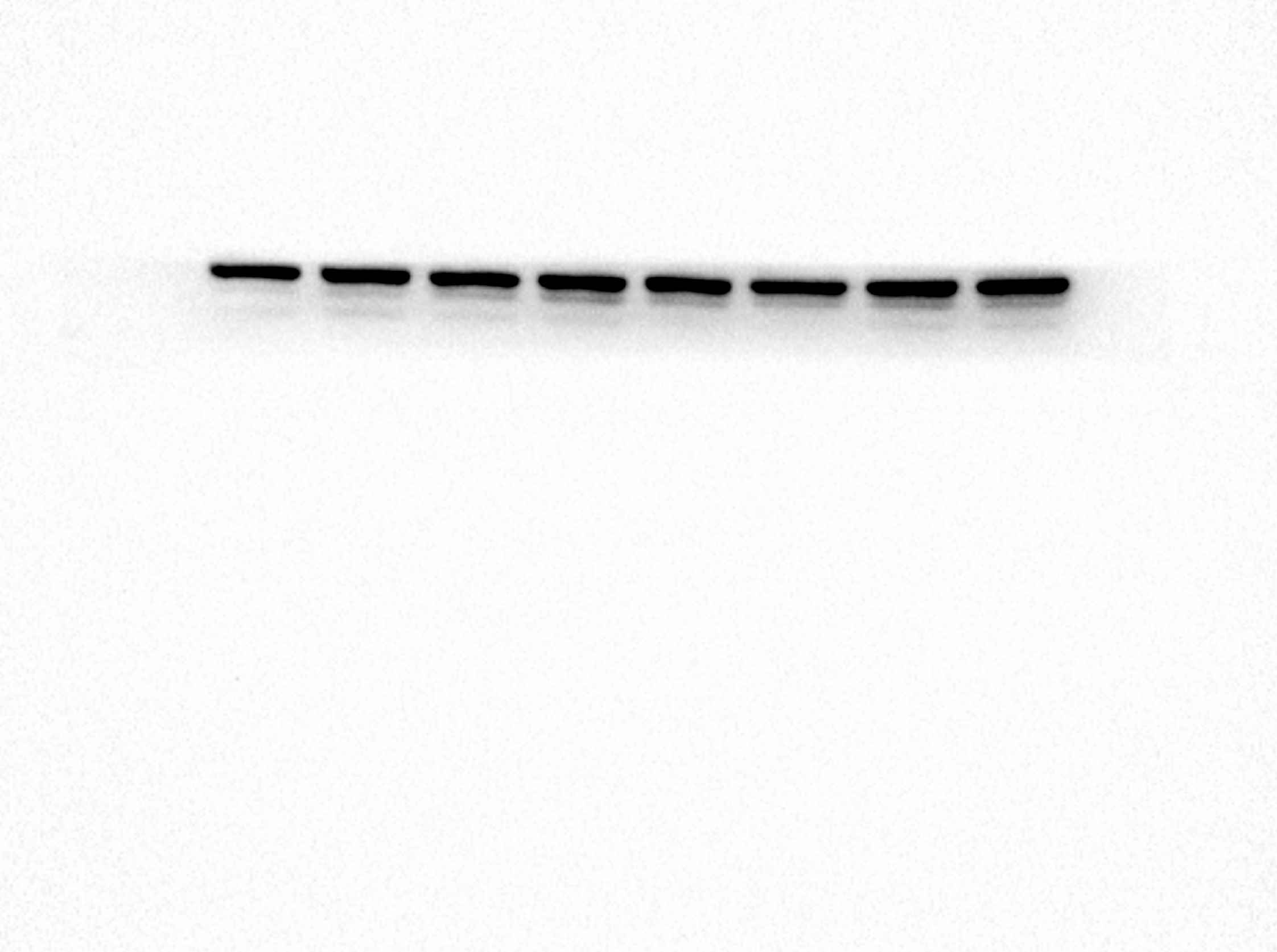

Supplement: Figure 2—source data 1. [file elife-83083-fig2-data1.zip › Figure 2-source data/Figure 2F STAT3.tif]

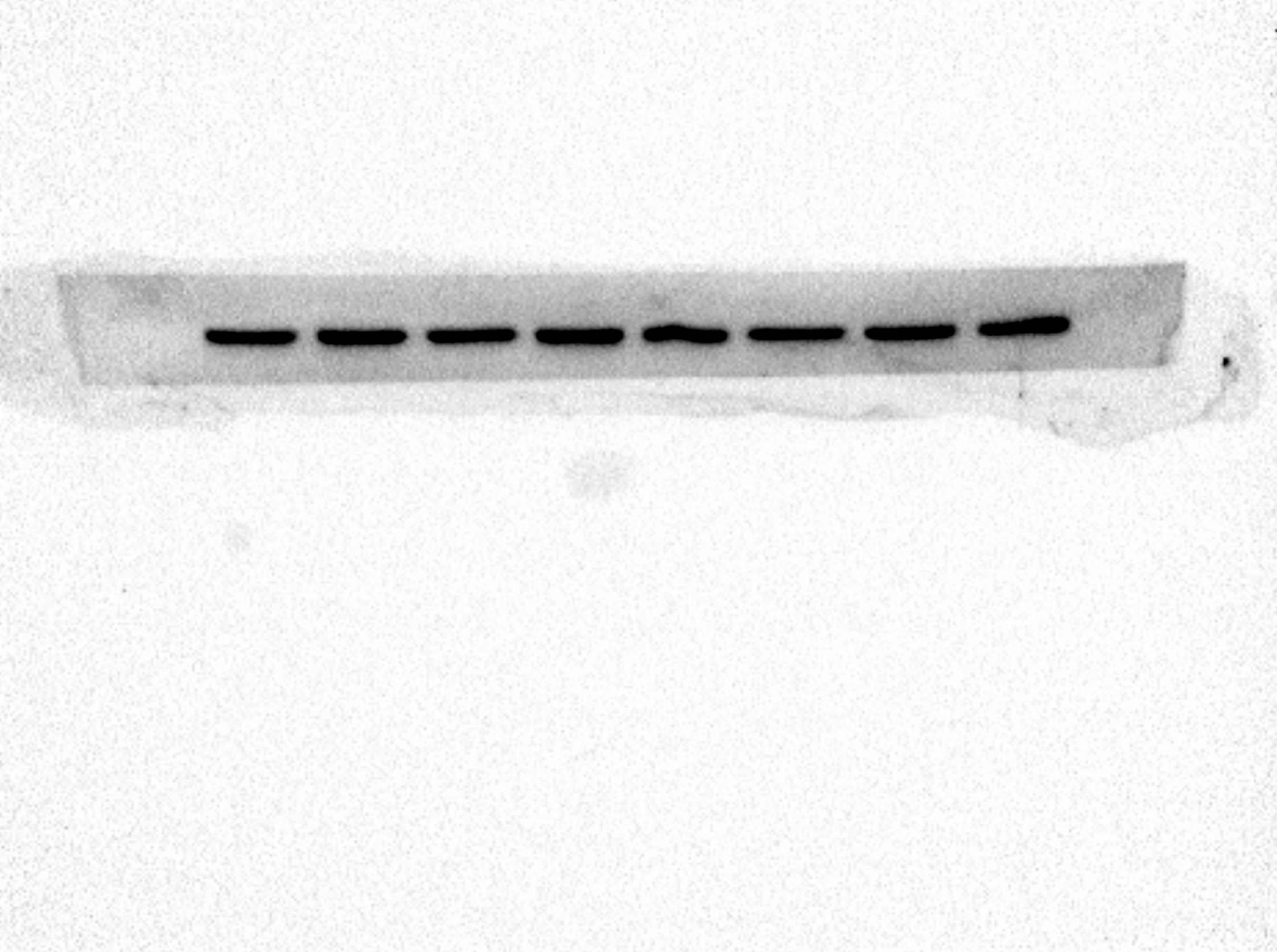

Supplement: Figure 2—source data 1. [file elife-83083-fig2-data1.zip › Figure 2-source data/Figure 2F a┬-Tubulin.tif]

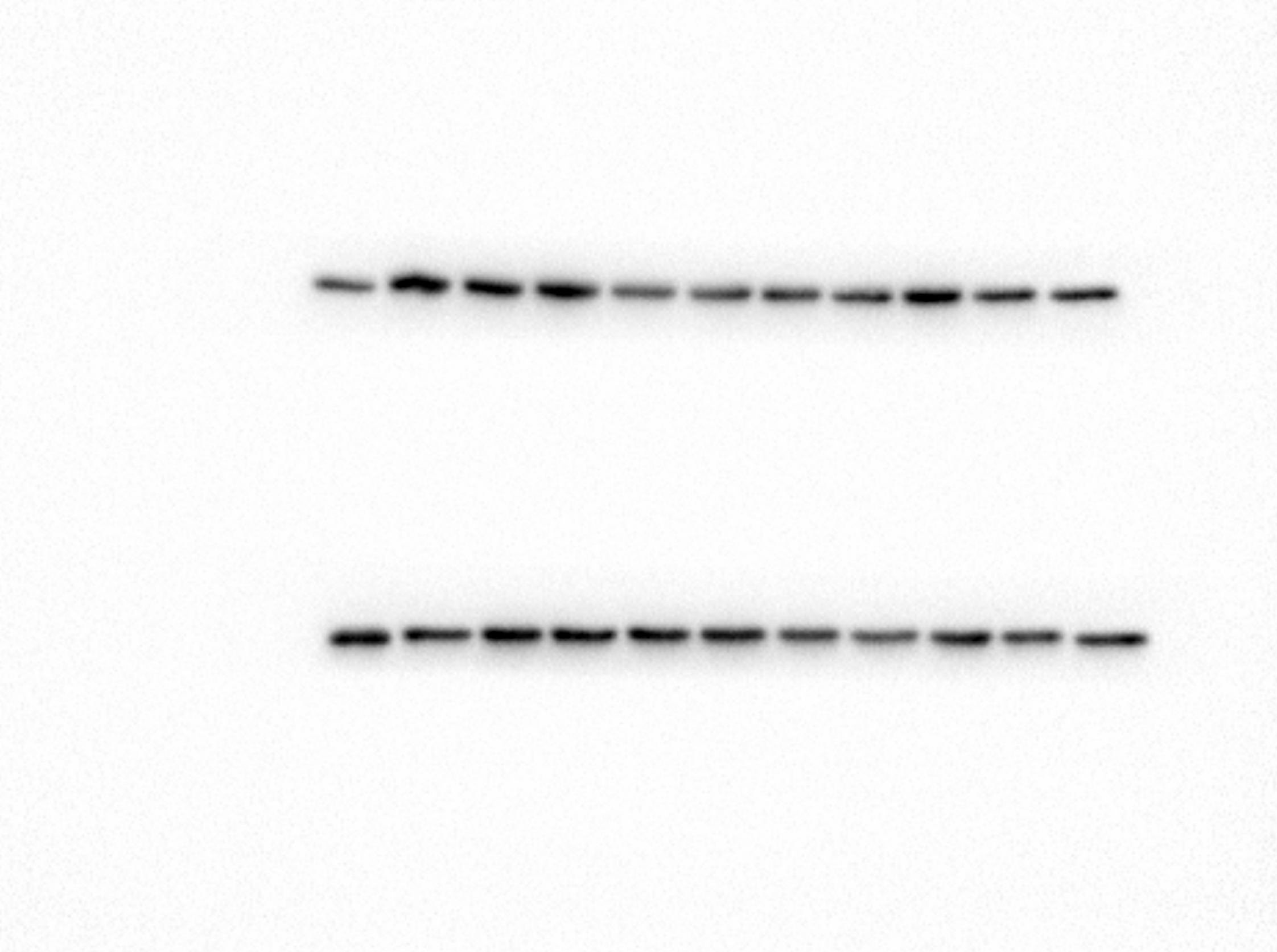

Supplement: Figure 2—source data 1. [file elife-83083-fig2-data1.zip › Figure 2-source data/Figure 2I GAPDH.tif]

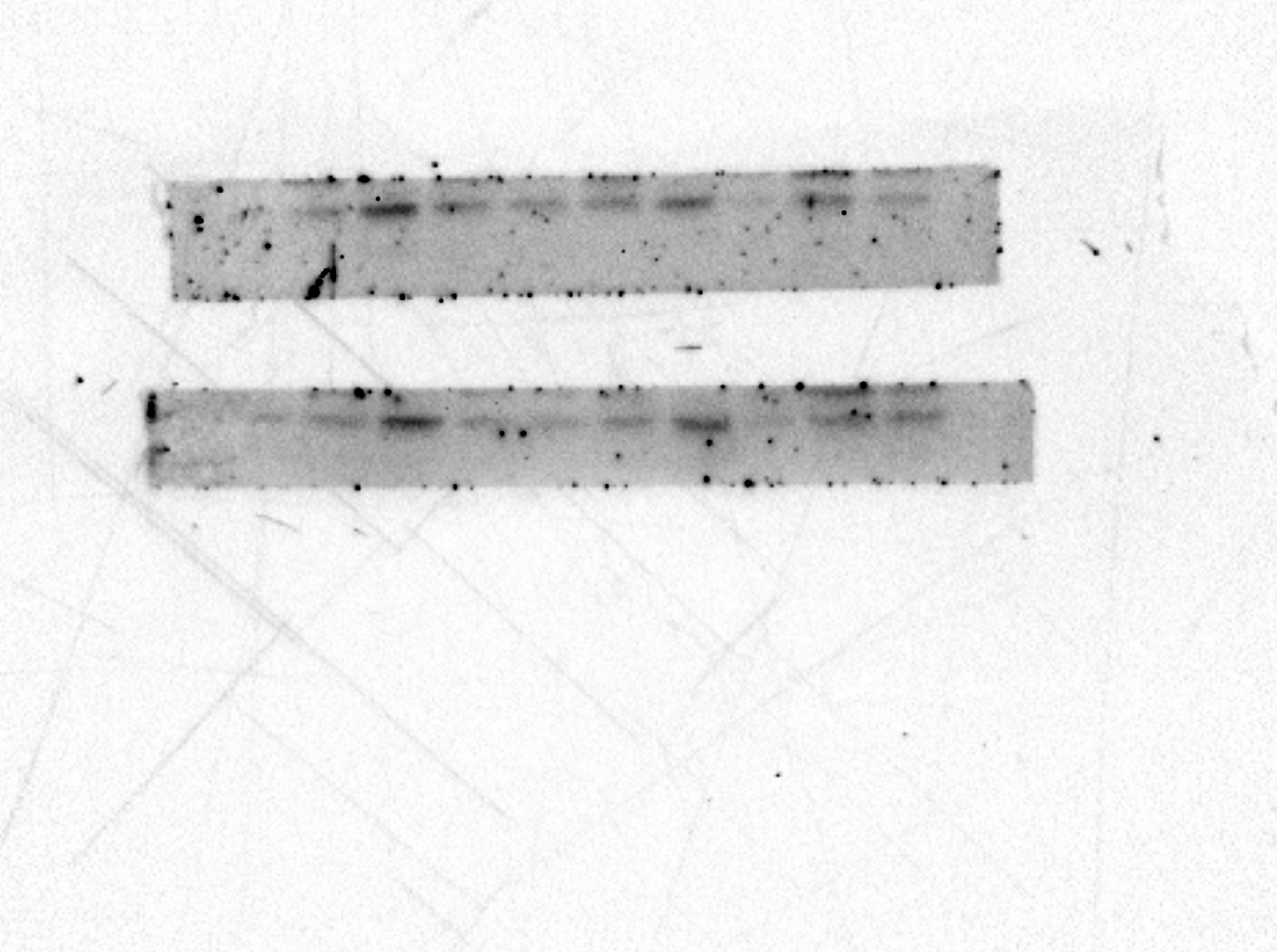

Supplement: Figure 2—source data 1. [file elife-83083-fig2-data1.zip › Figure 2-source data/Figure 2I IL-1a┬.tif]

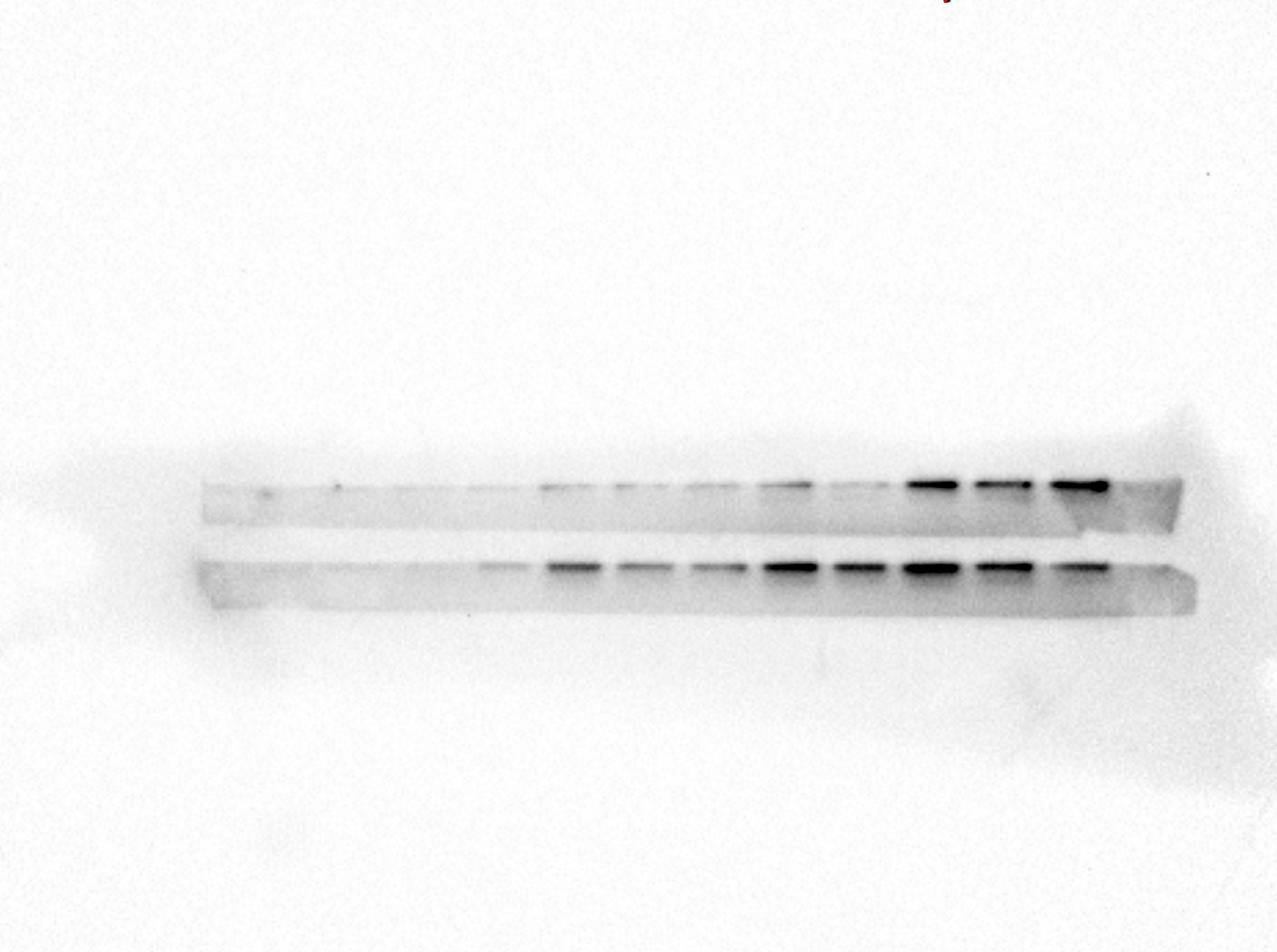

Supplement: Figure 2—source data 1. [file elife-83083-fig2-data1.zip › Figure 2-source data/Figure 2I p-STAT3.tif]

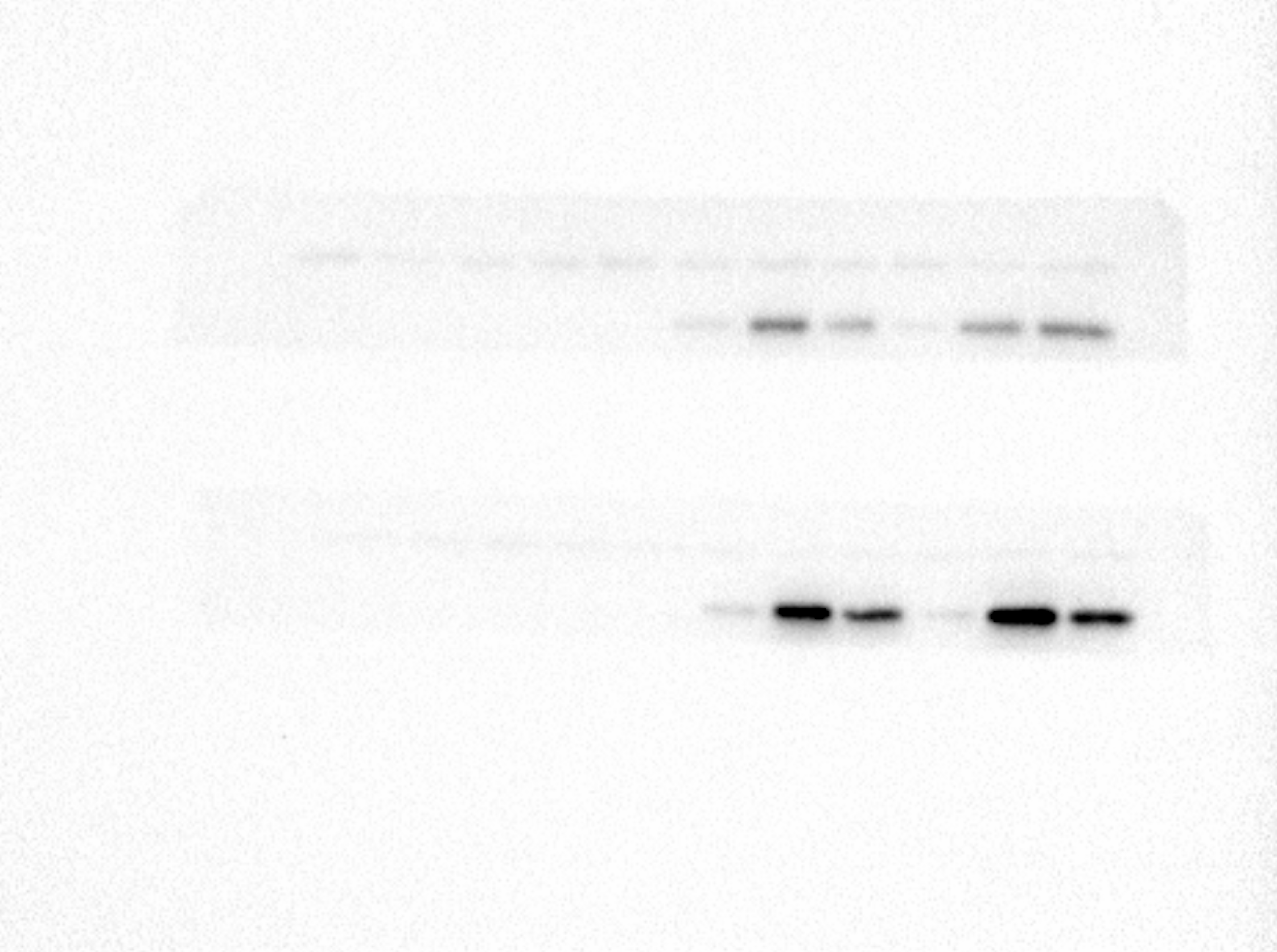

Supplement: Figure 2—source data 1. [file elife-83083-fig2-data1.zip › Figure 2-source data/Figure 2L IGFBP-1.tif]

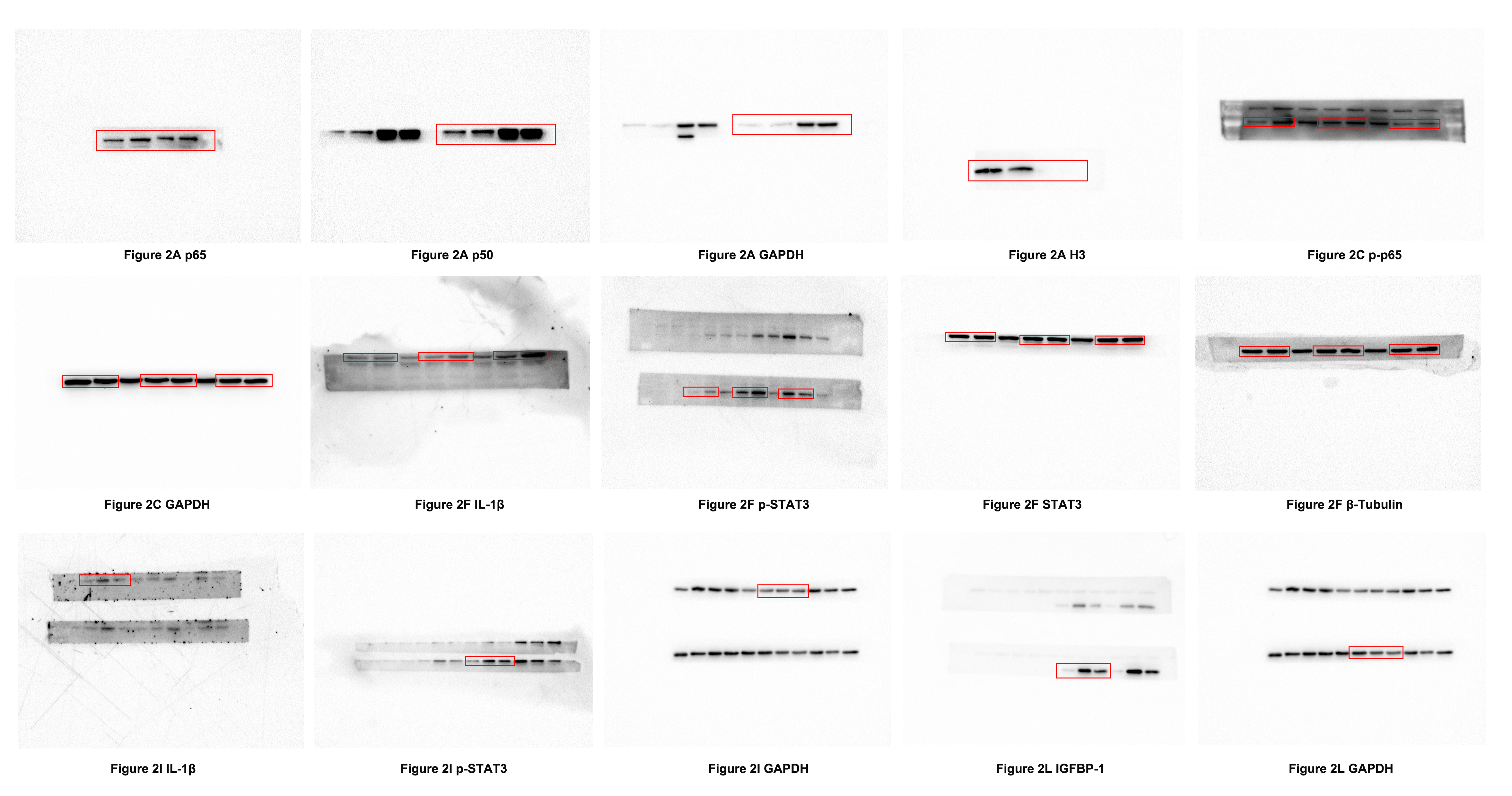

Supplement: Figure 2—source data 1. [file elife-83083-fig2-data1.zip › Figure 2-source data/Figure 2-source data.jpg]

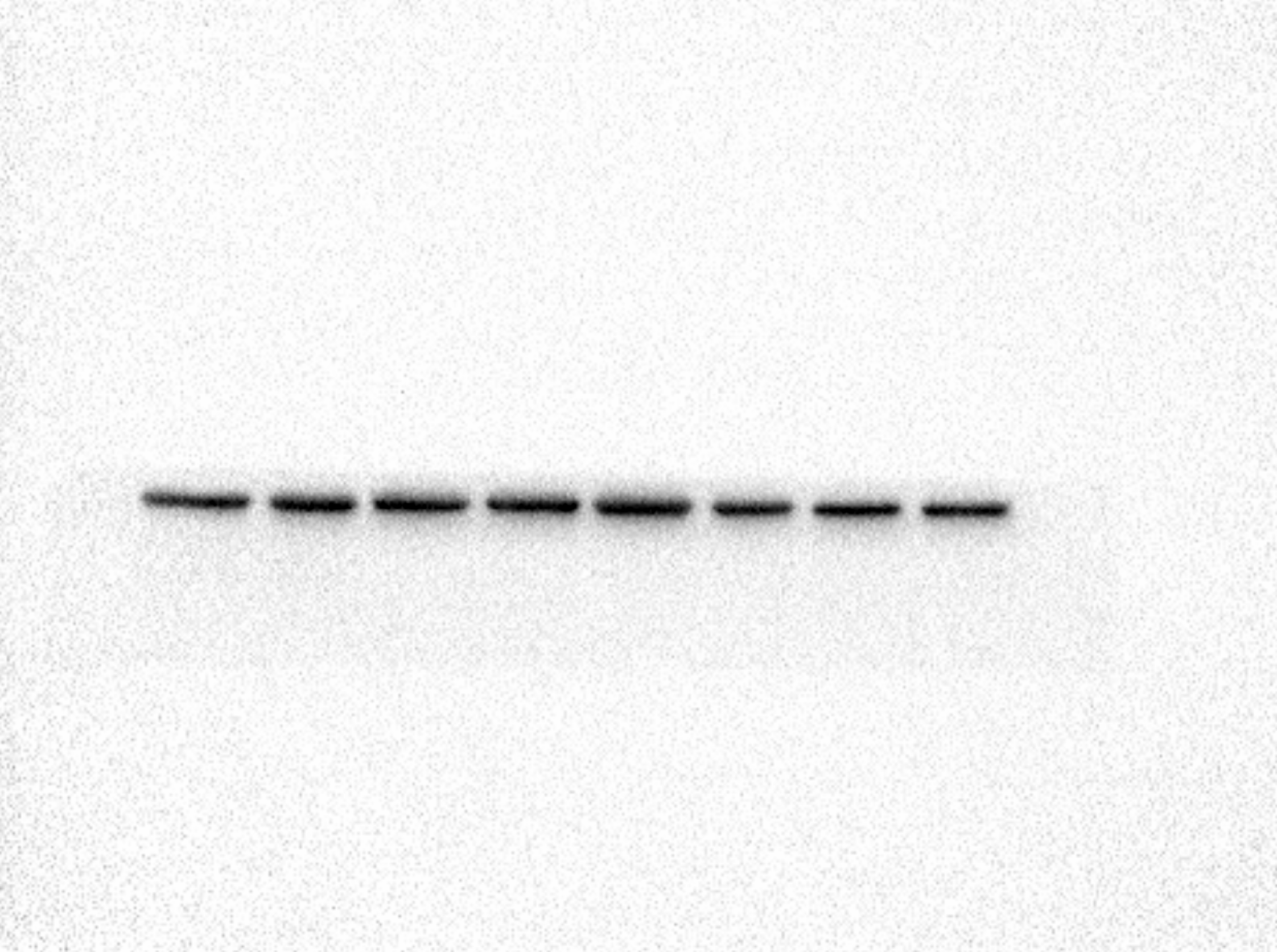

Supplement: Figure 3—source data 1. [file elife-83083-fig3-data1.zip › Figure 3-source data/Figure 3B GAPDH.tif]

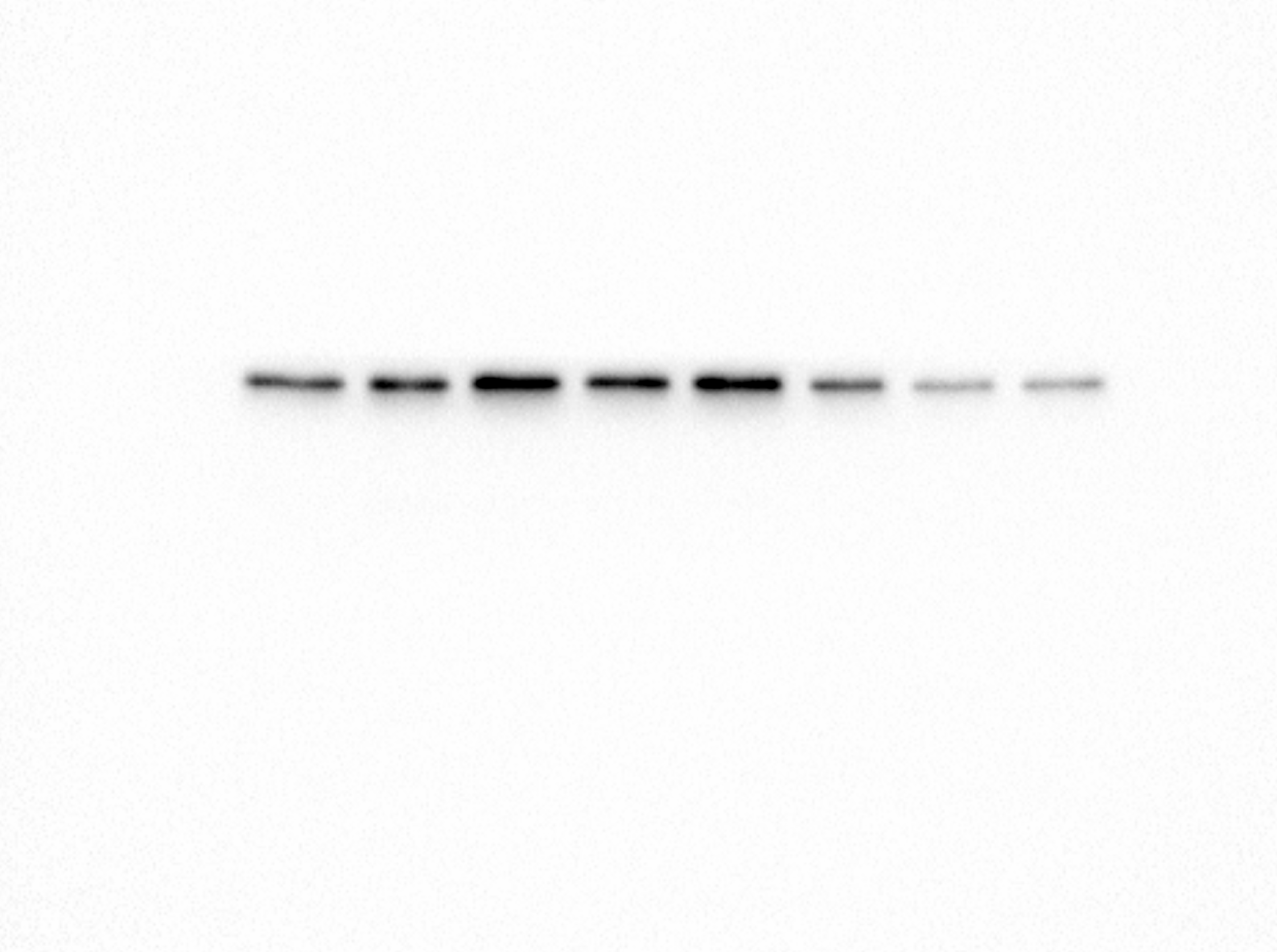

Supplement: Figure 3—source data 1. [file elife-83083-fig3-data1.zip › Figure 3-source data/Figure 3B Ia╩Ba┴.tif]

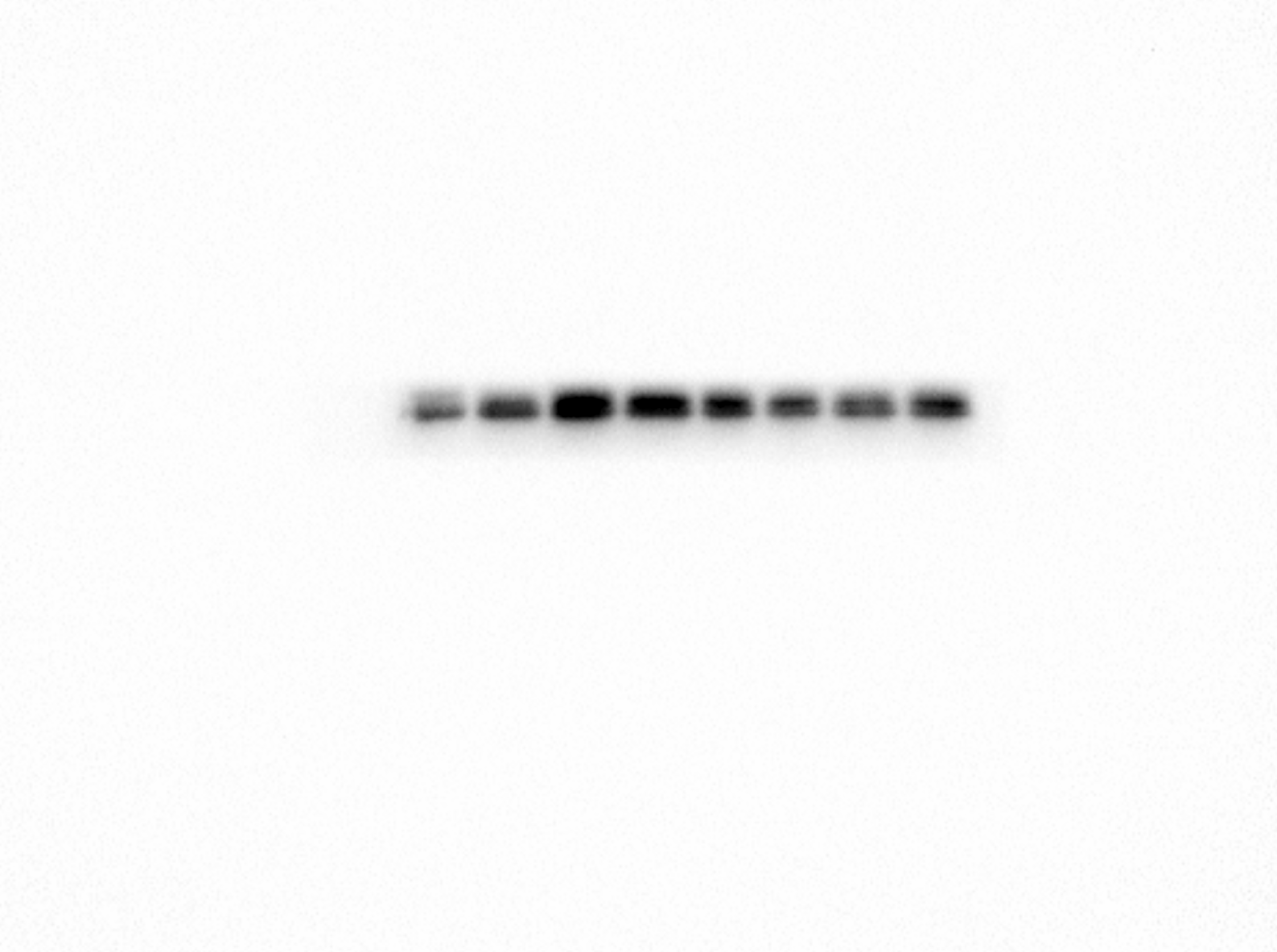

Supplement: Figure 3—source data 1. [file elife-83083-fig3-data1.zip › Figure 3-source data/Figure 3B p-Ia╩Ba┴.tif]

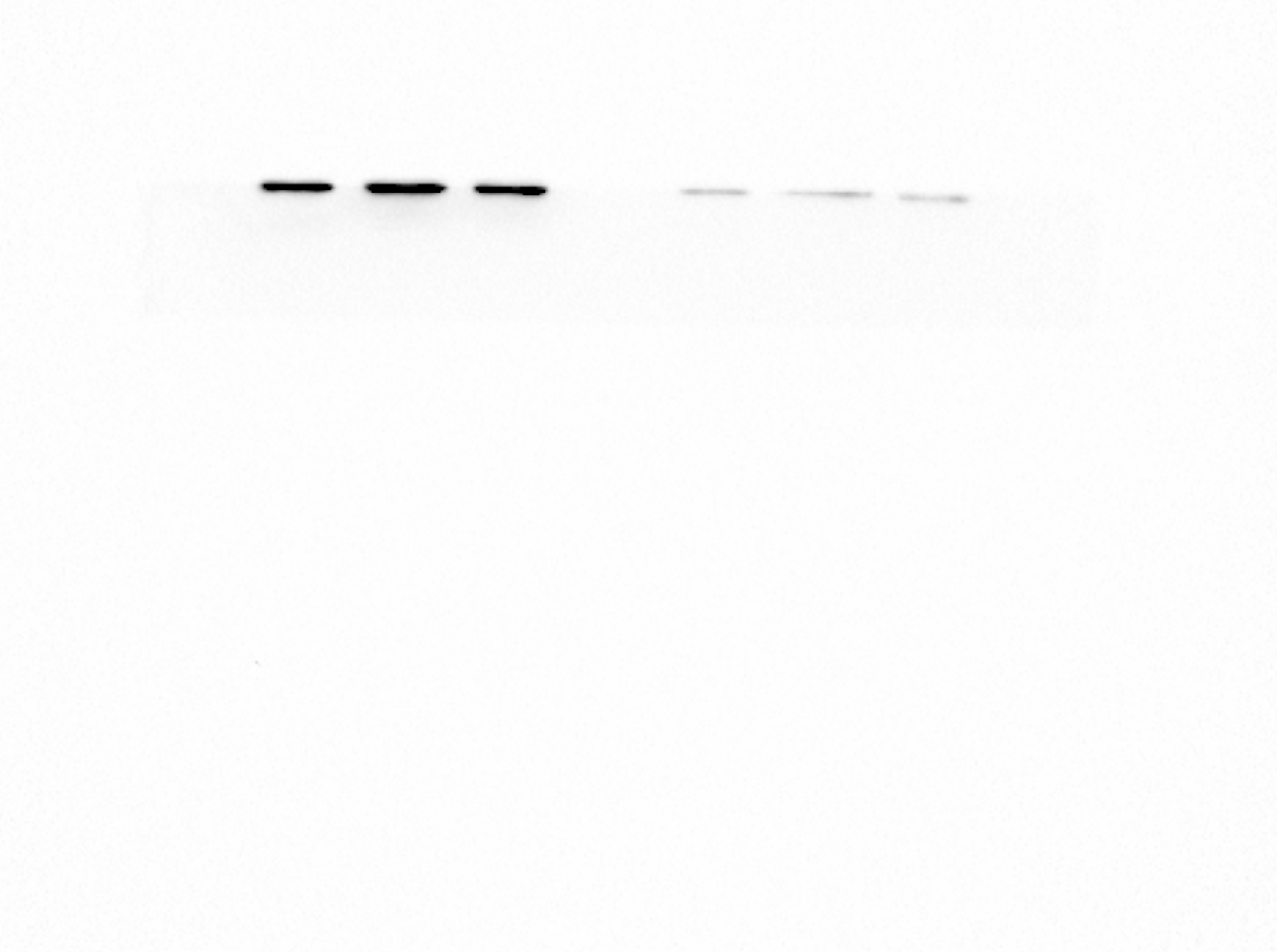

Supplement: Figure 3—source data 1. [file elife-83083-fig3-data1.zip › Figure 3-source data/Figure 3D GAPDH.tif]

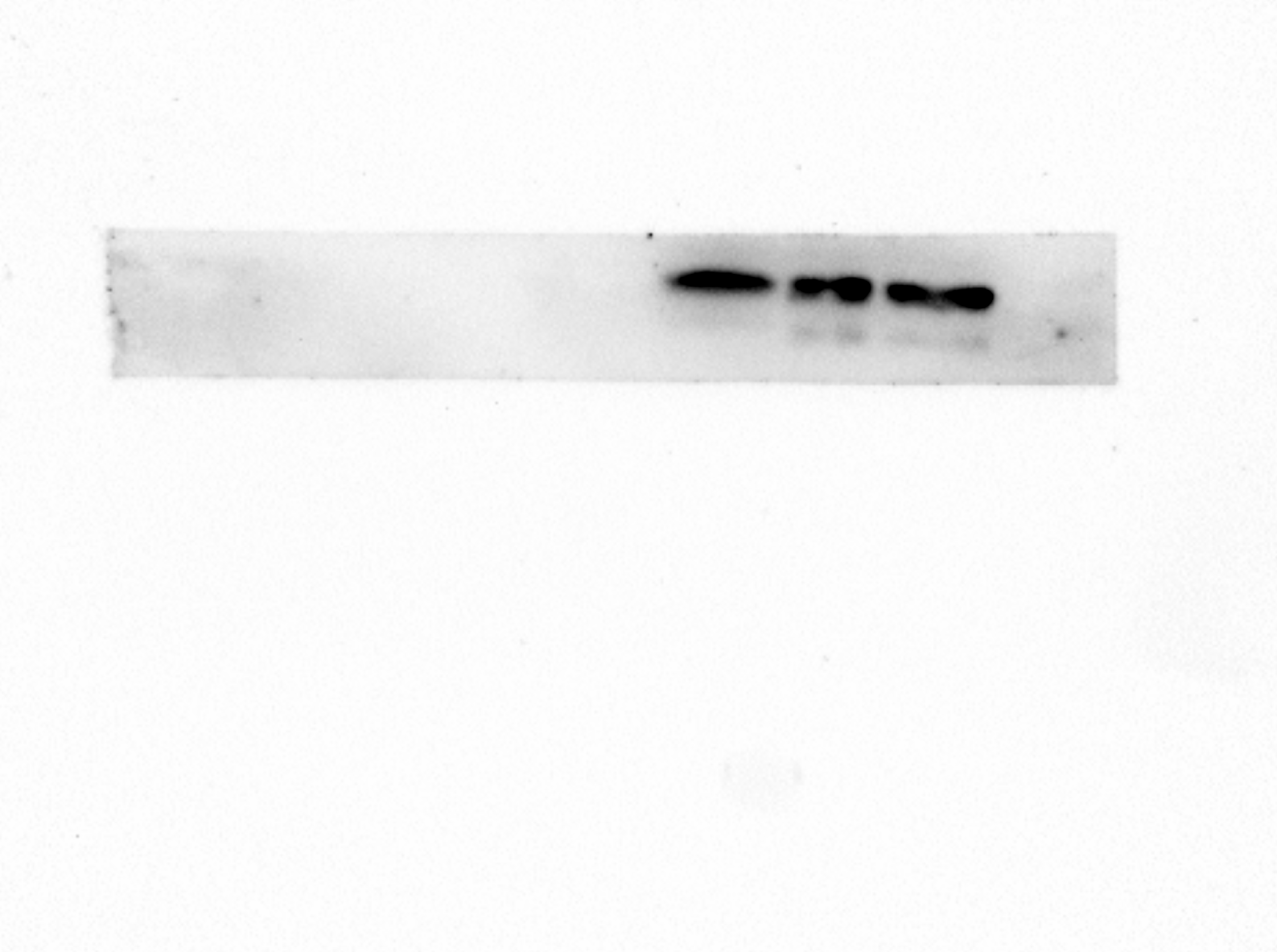

Supplement: Figure 3—source data 1. [file elife-83083-fig3-data1.zip › Figure 3-source data/Figure 3D H3.tif]

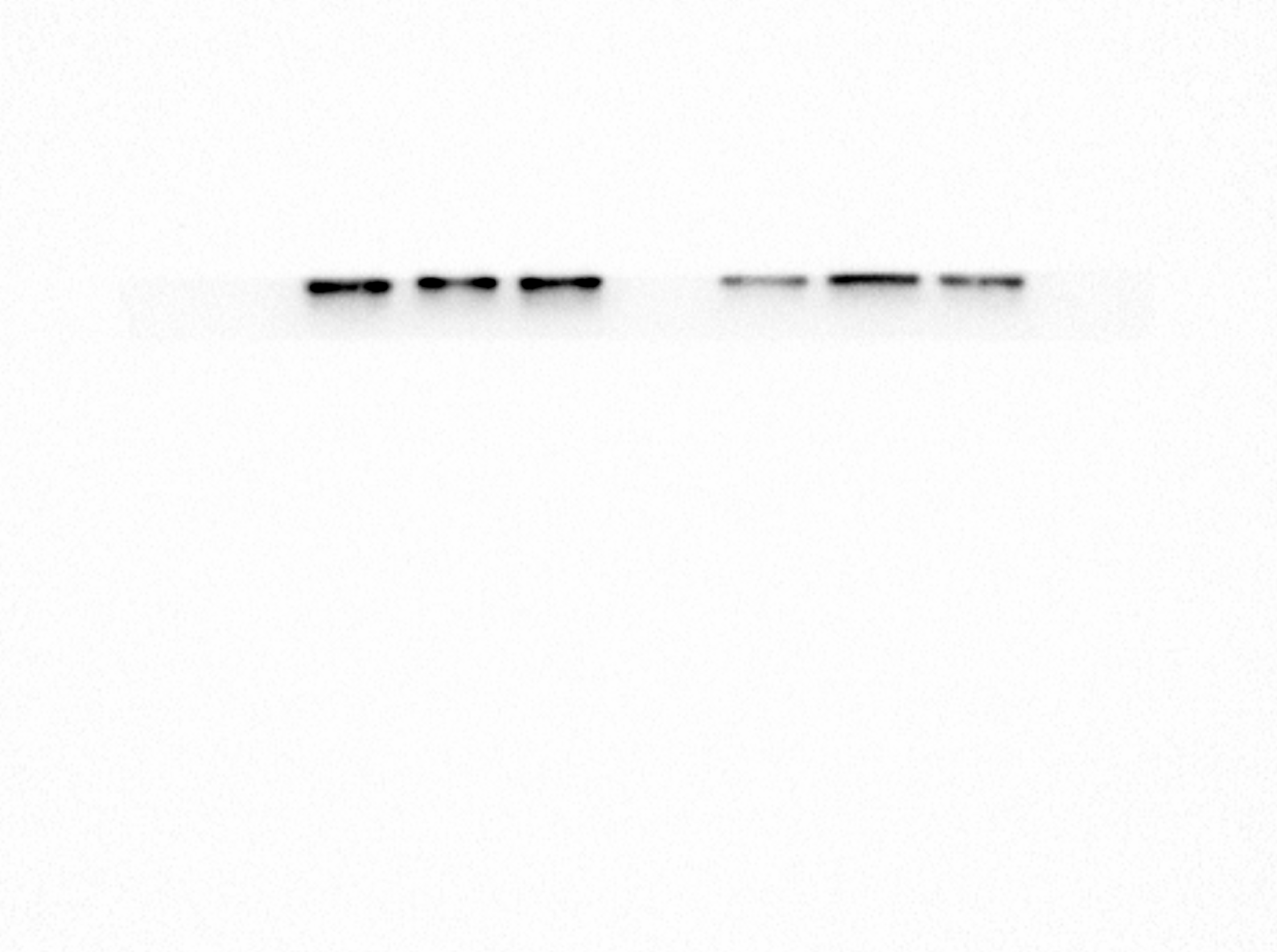

Supplement: Figure 3—source data 1. [file elife-83083-fig3-data1.zip › Figure 3-source data/Figure 3D p50.tif]

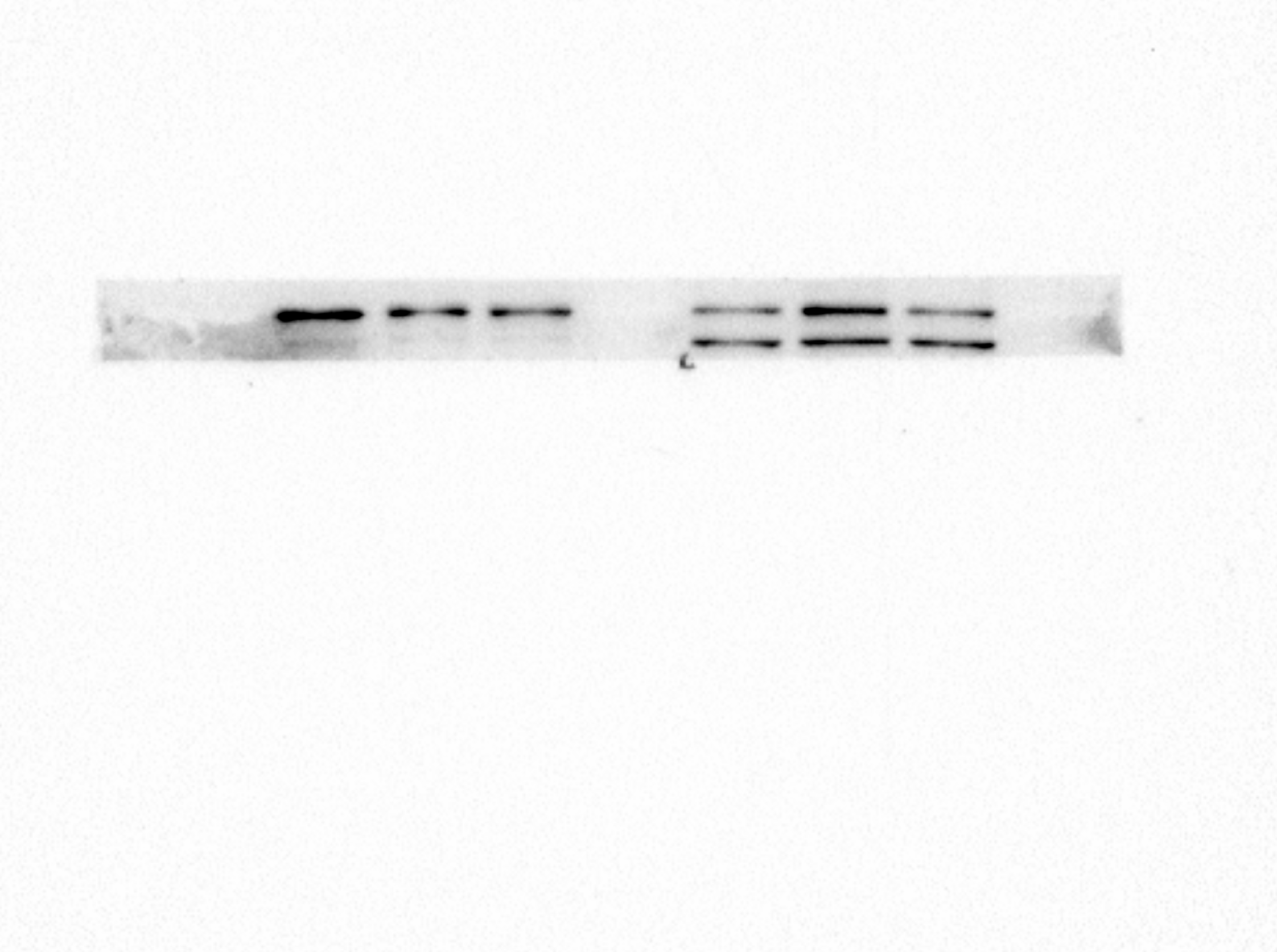

Supplement: Figure 3—source data 1. [file elife-83083-fig3-data1.zip › Figure 3-source data/Figure 3D p65.tif]

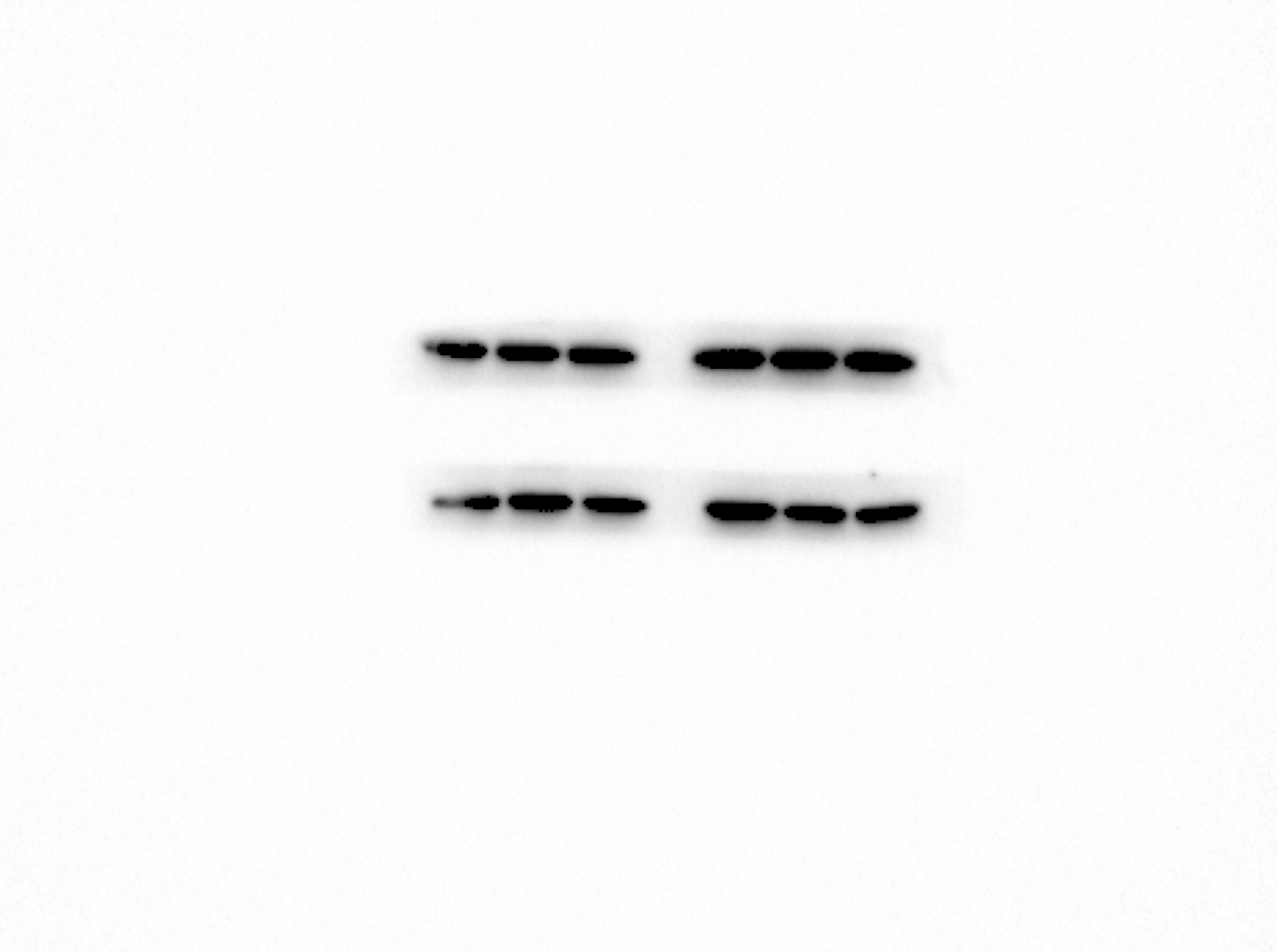

Supplement: Figure 3—source data 1. [file elife-83083-fig3-data1.zip › Figure 3-source data/Figure 3F GAPDH.tif]

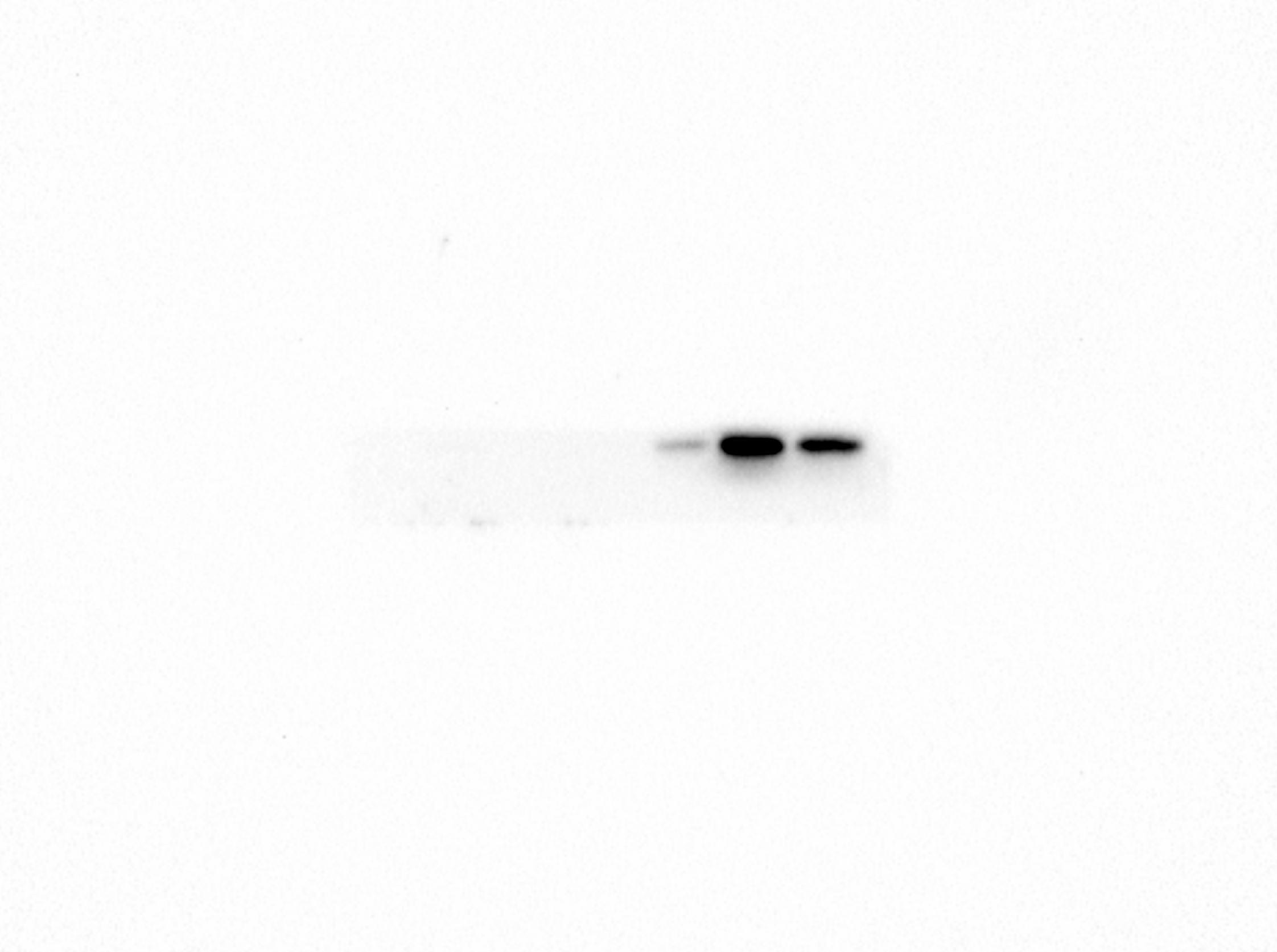

Supplement: Figure 3—source data 1. [file elife-83083-fig3-data1.zip › Figure 3-source data/Figure 3F IGFBP-1.tif]

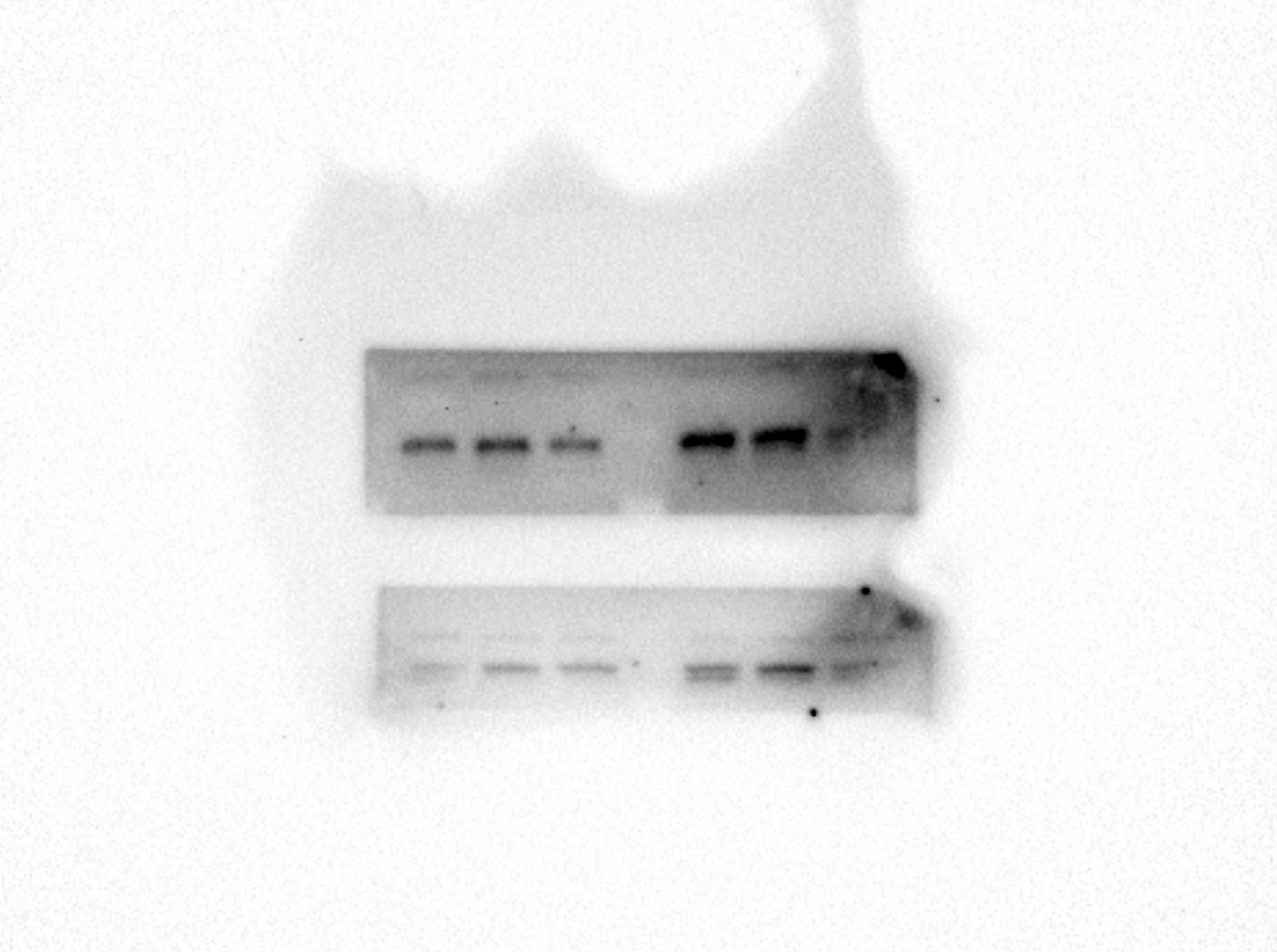

Supplement: Figure 3—source data 1. [file elife-83083-fig3-data1.zip › Figure 3-source data/Figure 3F p-STAT3.tif]

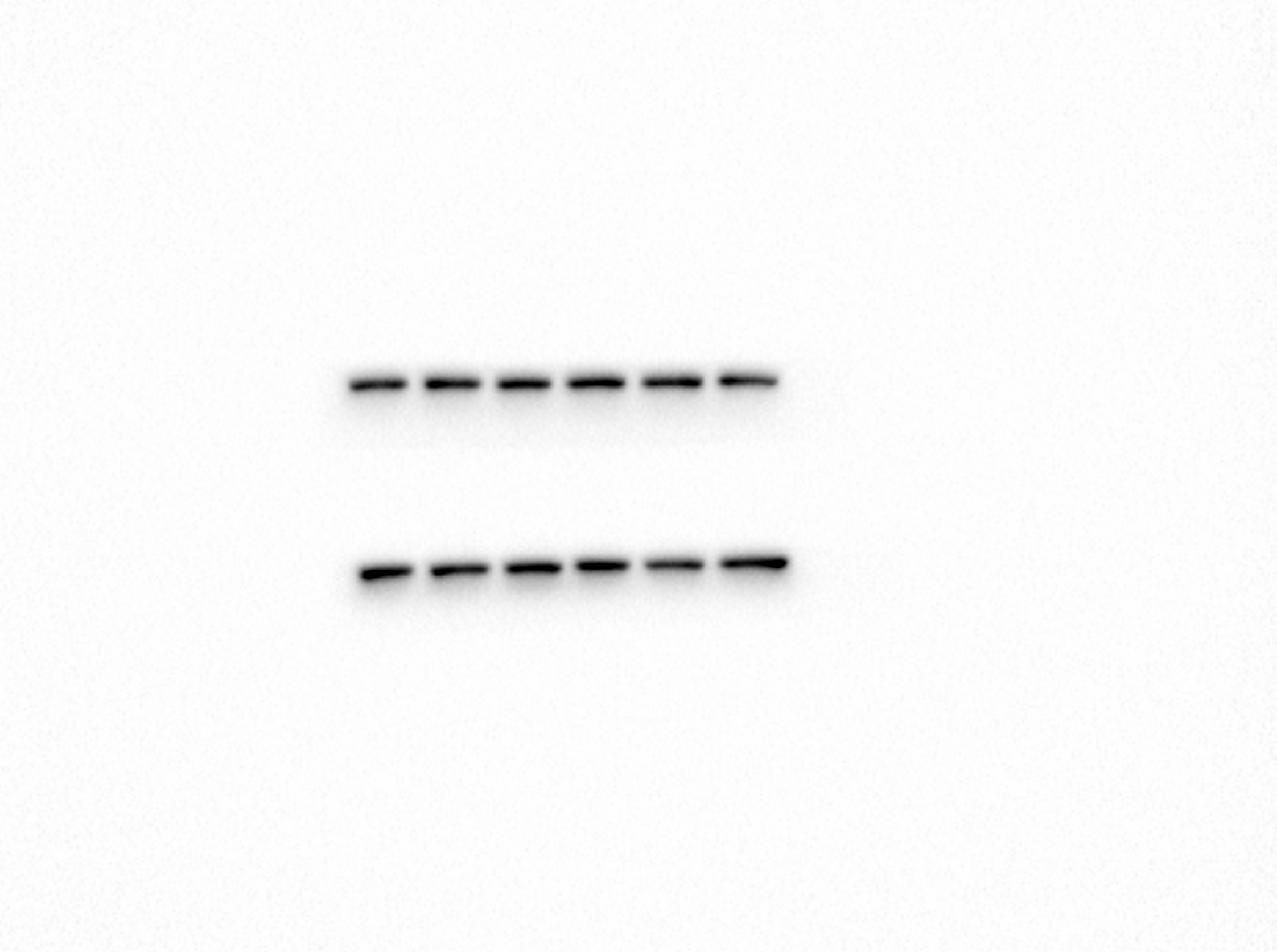

Supplement: Figure 3—source data 1. [file elife-83083-fig3-data1.zip › Figure 3-source data/Figure 3F STAT3.tif]

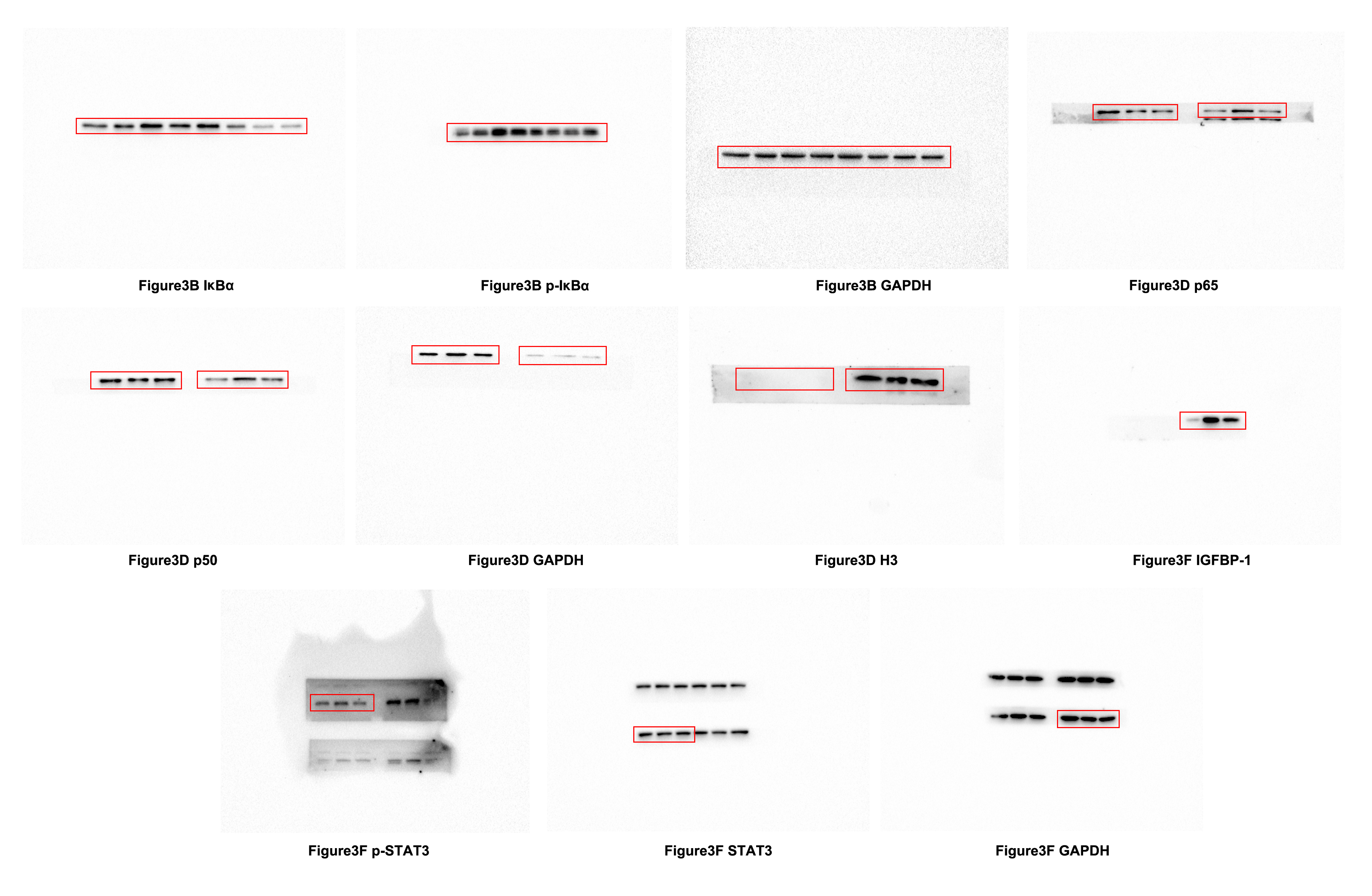

Supplement: Figure 3—source data 1. [file elife-83083-fig3-data1.zip › Figure 3-source data/Figure 3-source data.jpg]

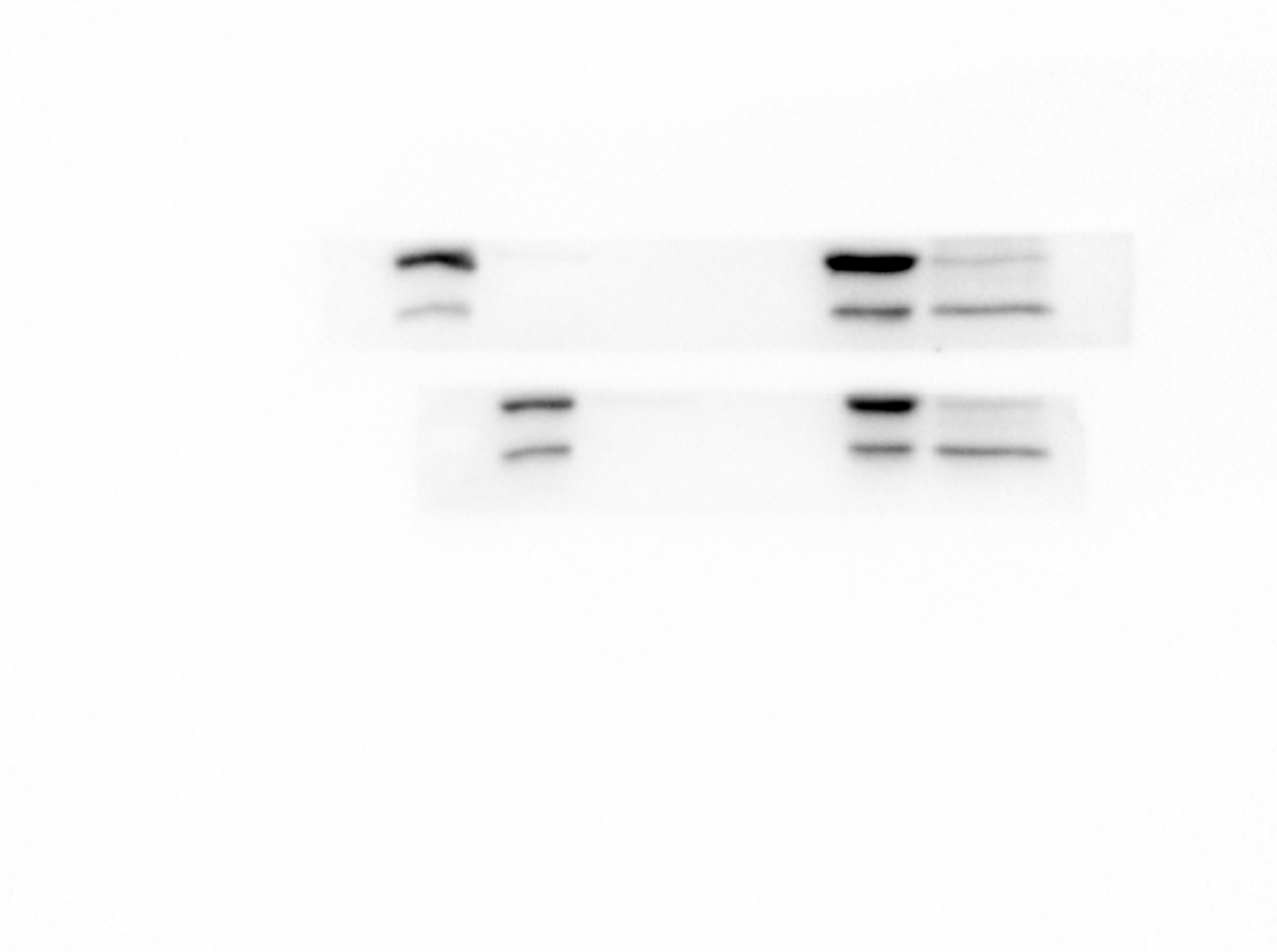

Supplement: Figure 4—source data 1. [file elife-83083-fig4-data1.zip › Figure 4-source data/Figure 4A GAPDH.tif]

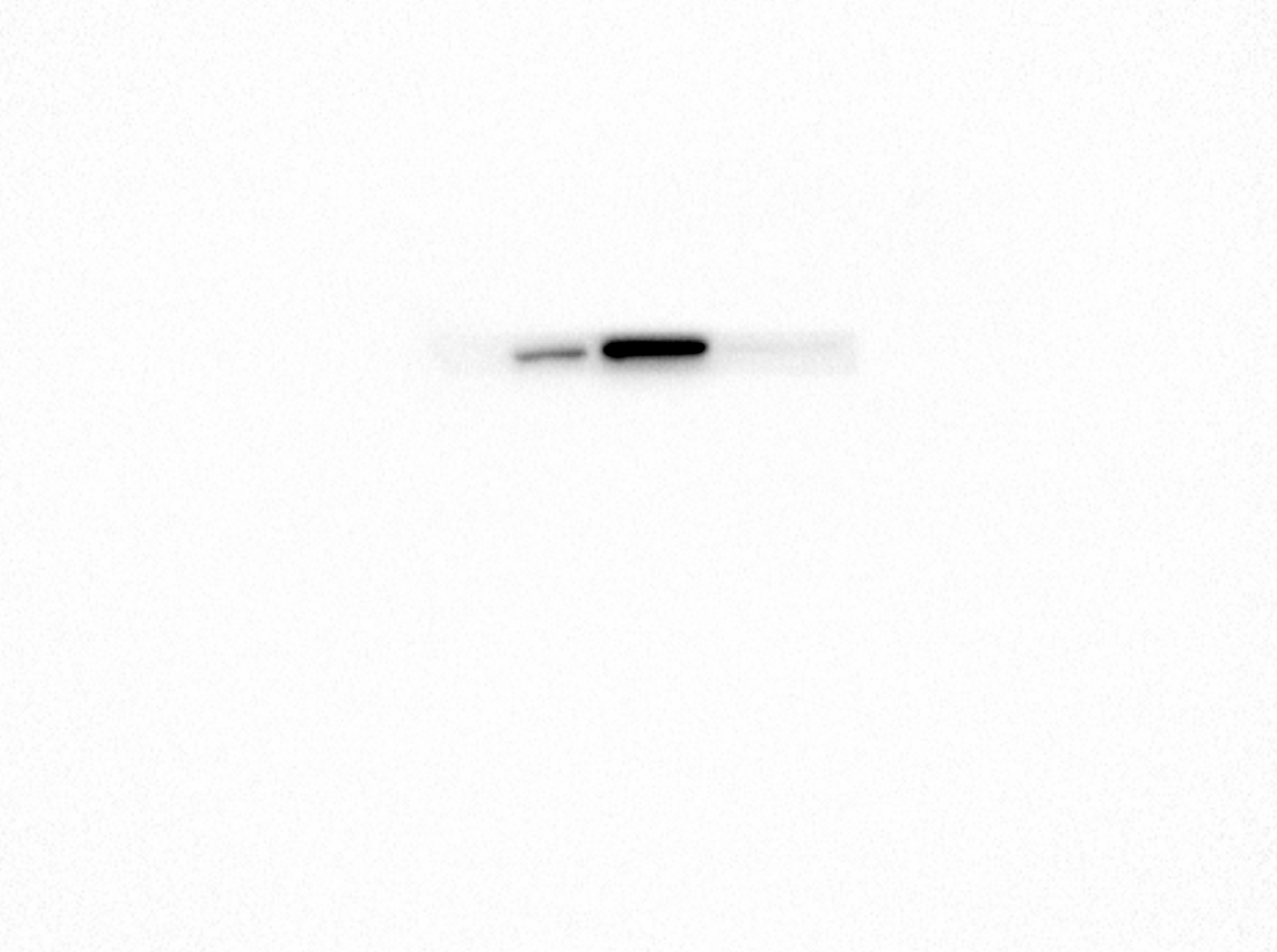

Supplement: Figure 4—source data 1. [file elife-83083-fig4-data1.zip › Figure 4-source data/Figure 4A Ga┴q.tif]

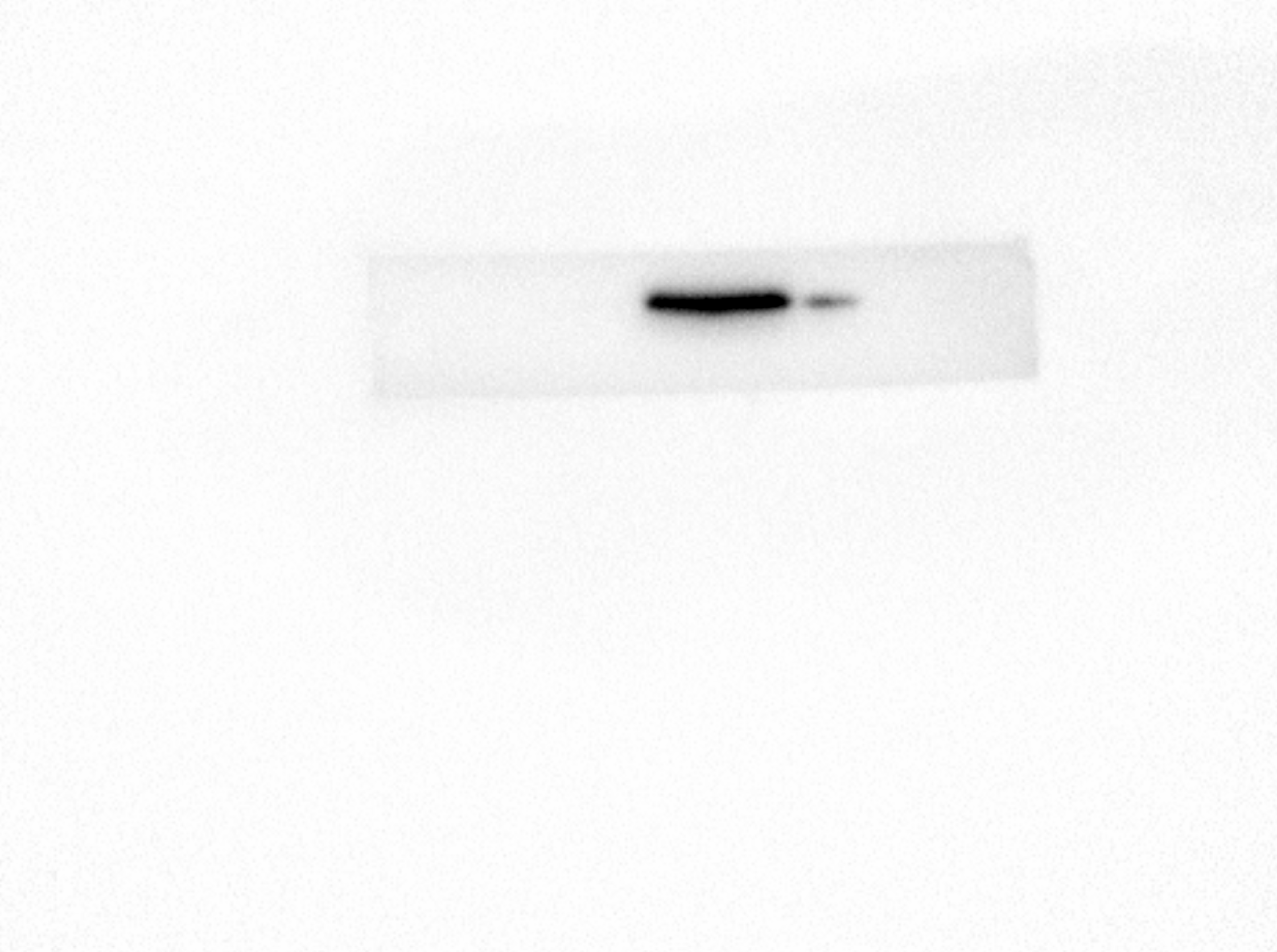

Supplement: Figure 4—source data 1. [file elife-83083-fig4-data1.zip › Figure 4-source data/Figure 4A H3.tif]

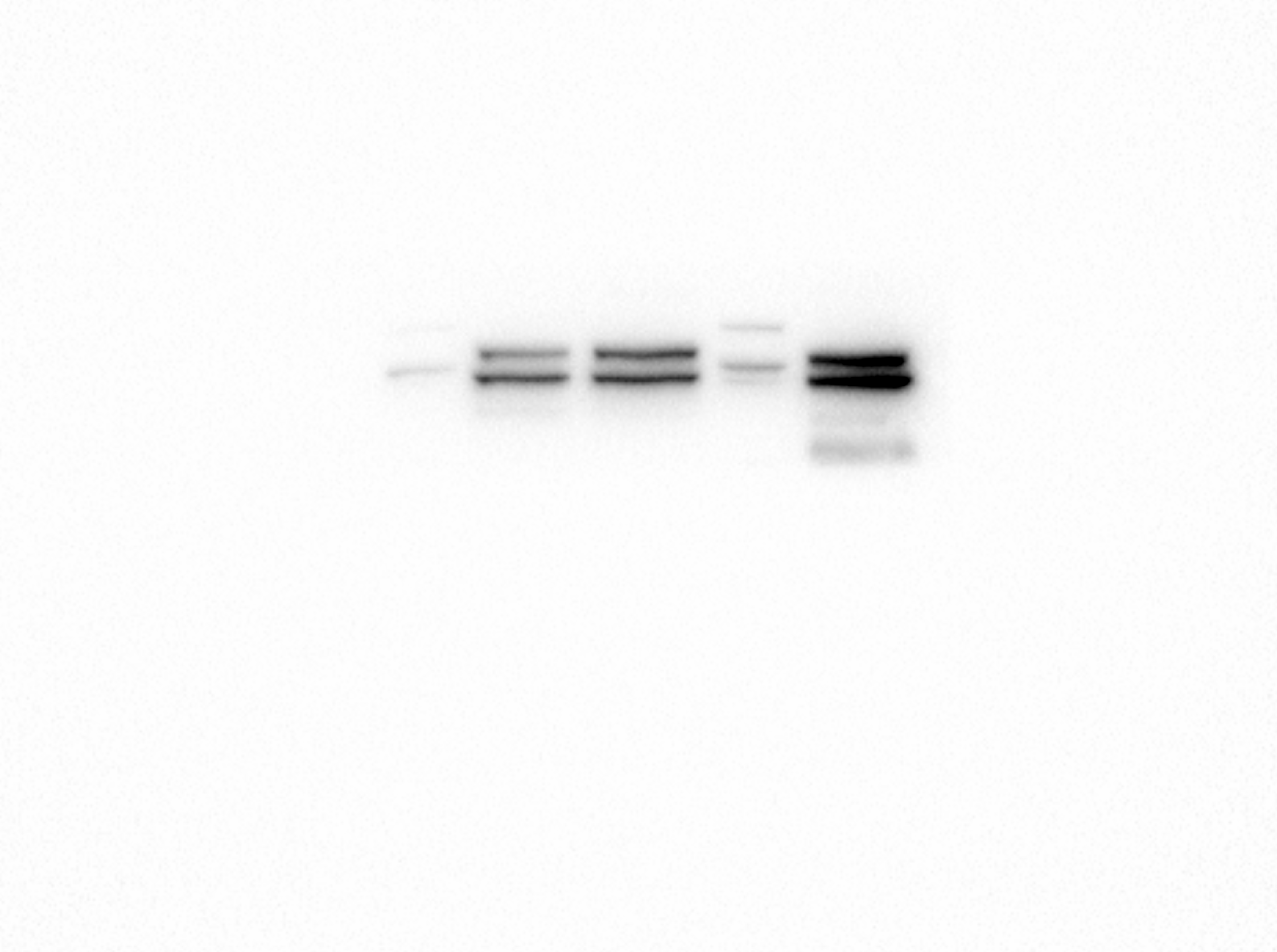

Supplement: Figure 4—source data 1. [file elife-83083-fig4-data1.zip › Figure 4-source data/Figure 4A LaminAC.tif]

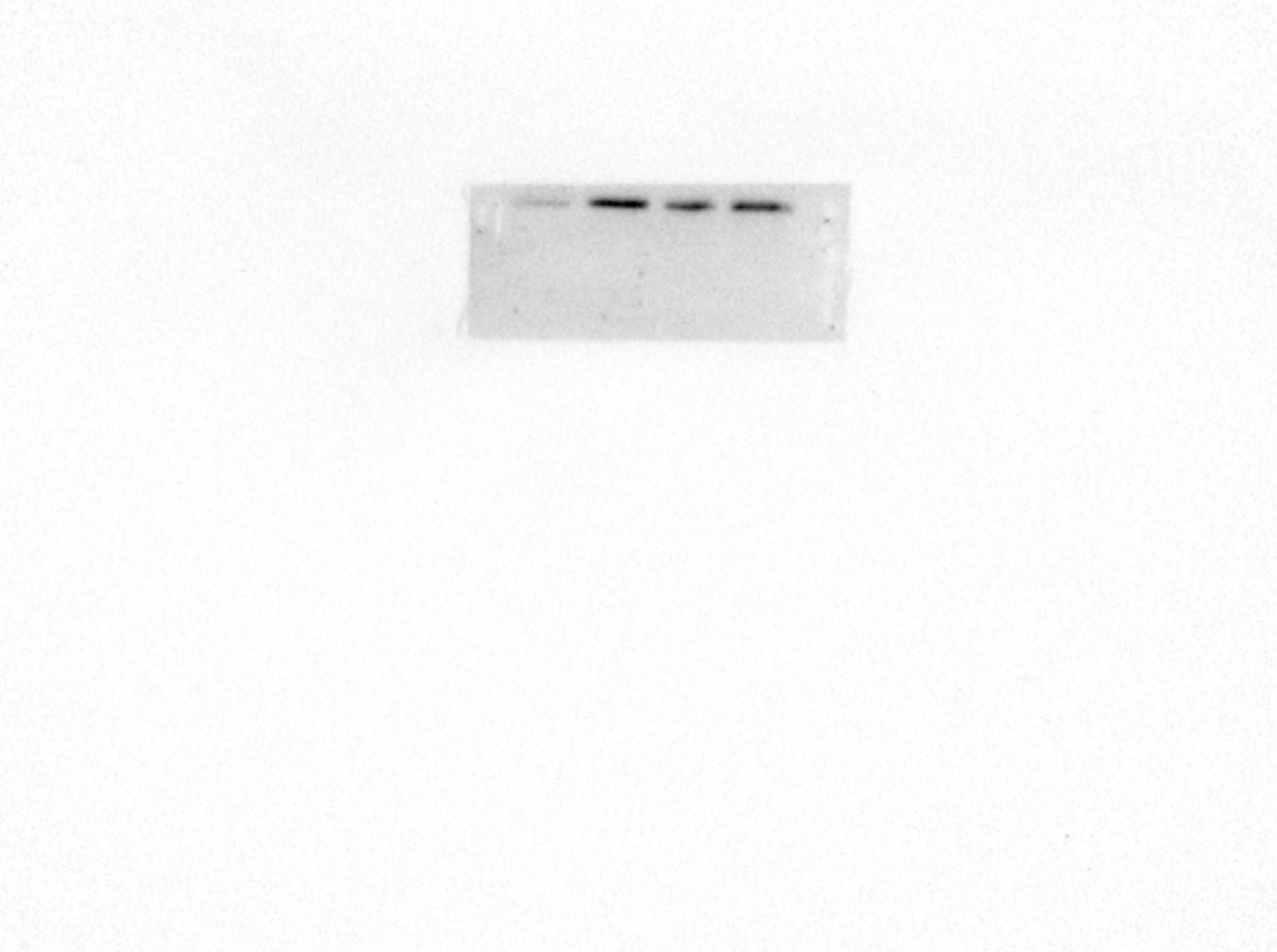

Supplement: Figure 4—source data 1. [file elife-83083-fig4-data1.zip › Figure 4-source data/Figure 4F IGFBP-1.tif]

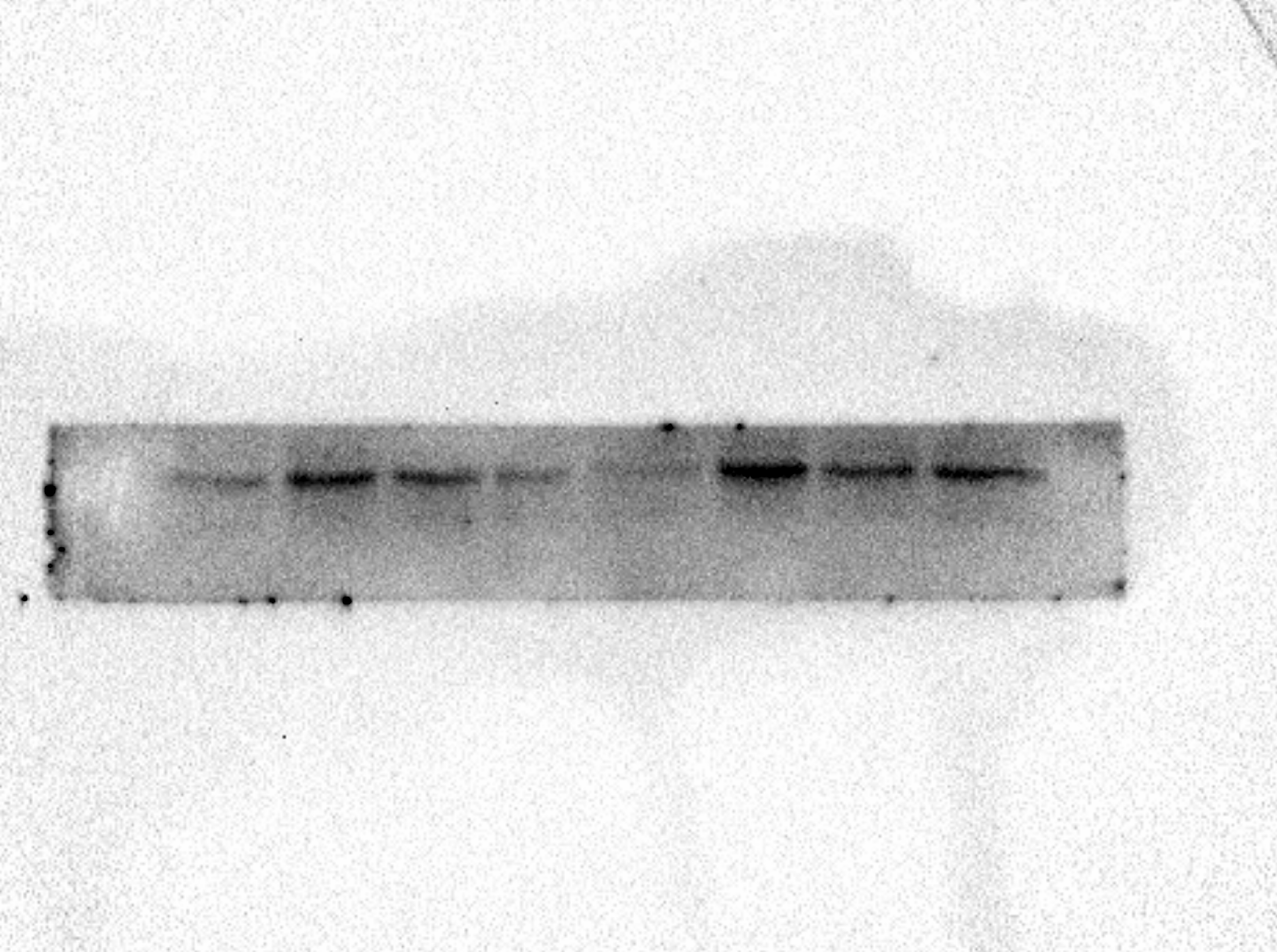

Supplement: Figure 4—source data 1. [file elife-83083-fig4-data1.zip › Figure 4-source data/Figure 4F IL-1a┬.tif]

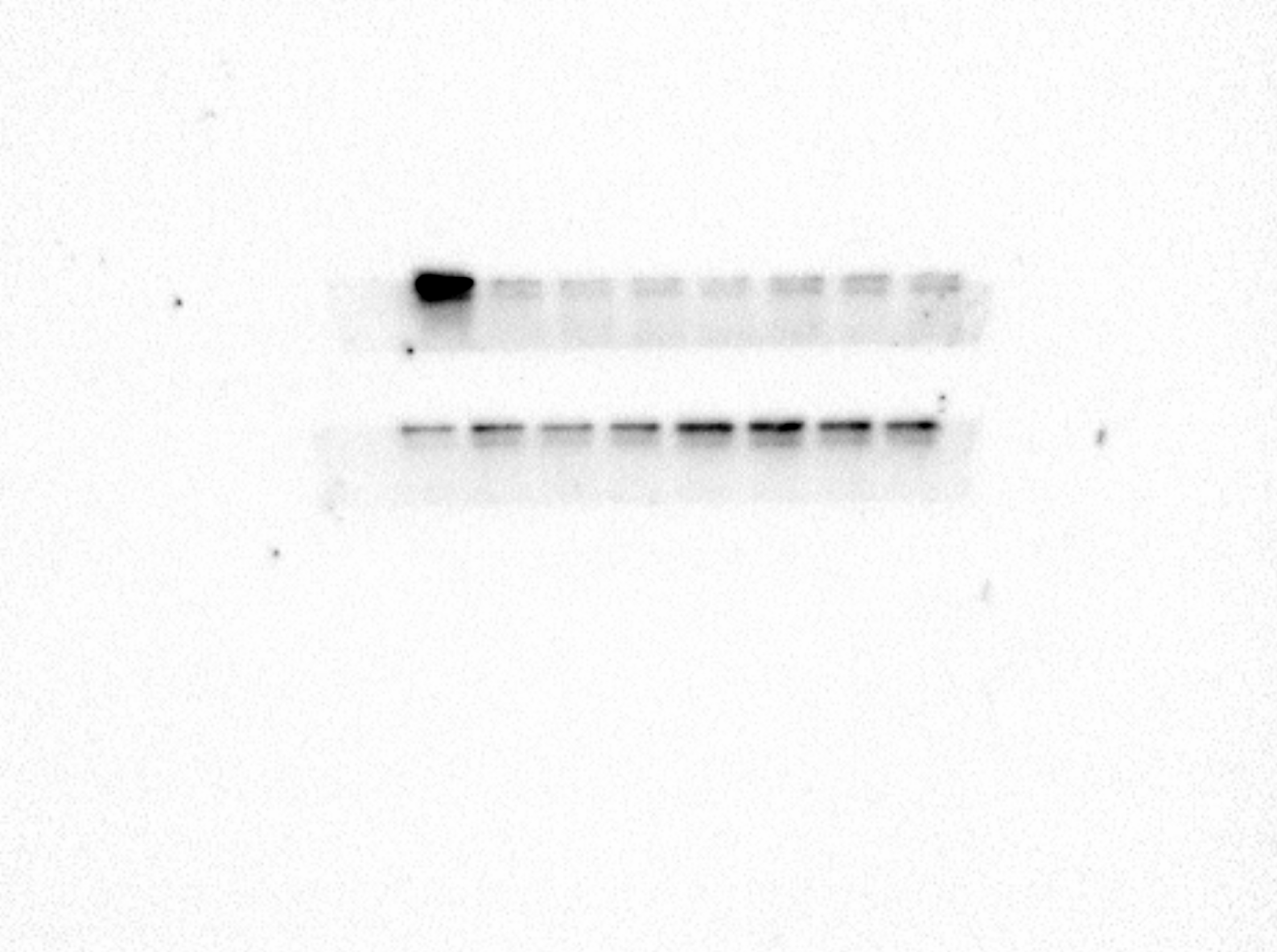

Supplement: Figure 4—source data 1. [file elife-83083-fig4-data1.zip › Figure 4-source data/Figure 4F p-STAT3.tif]

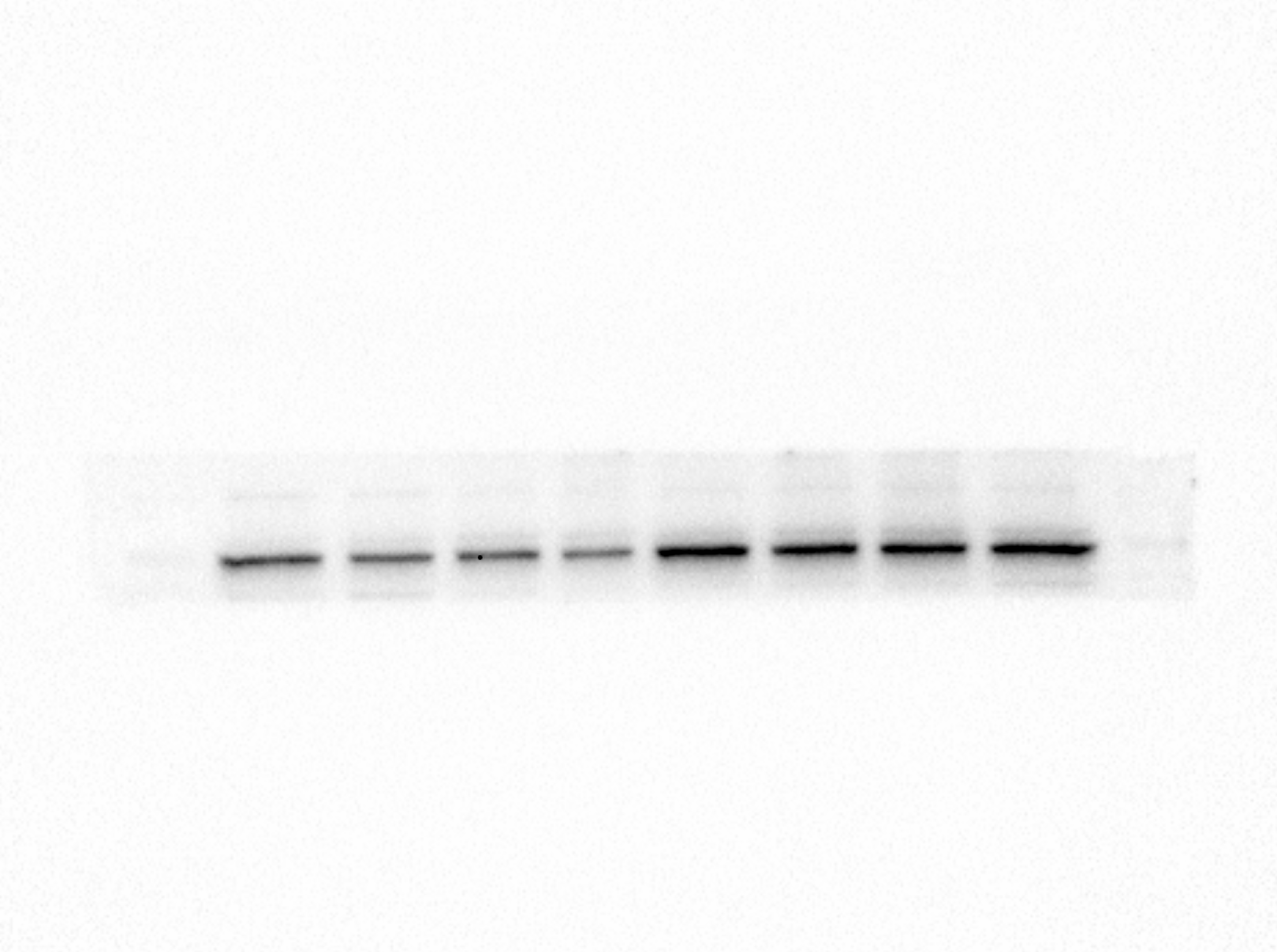

Supplement: Figure 4—source data 1. [file elife-83083-fig4-data1.zip › Figure 4-source data/Figure 4F STAT3.tif]

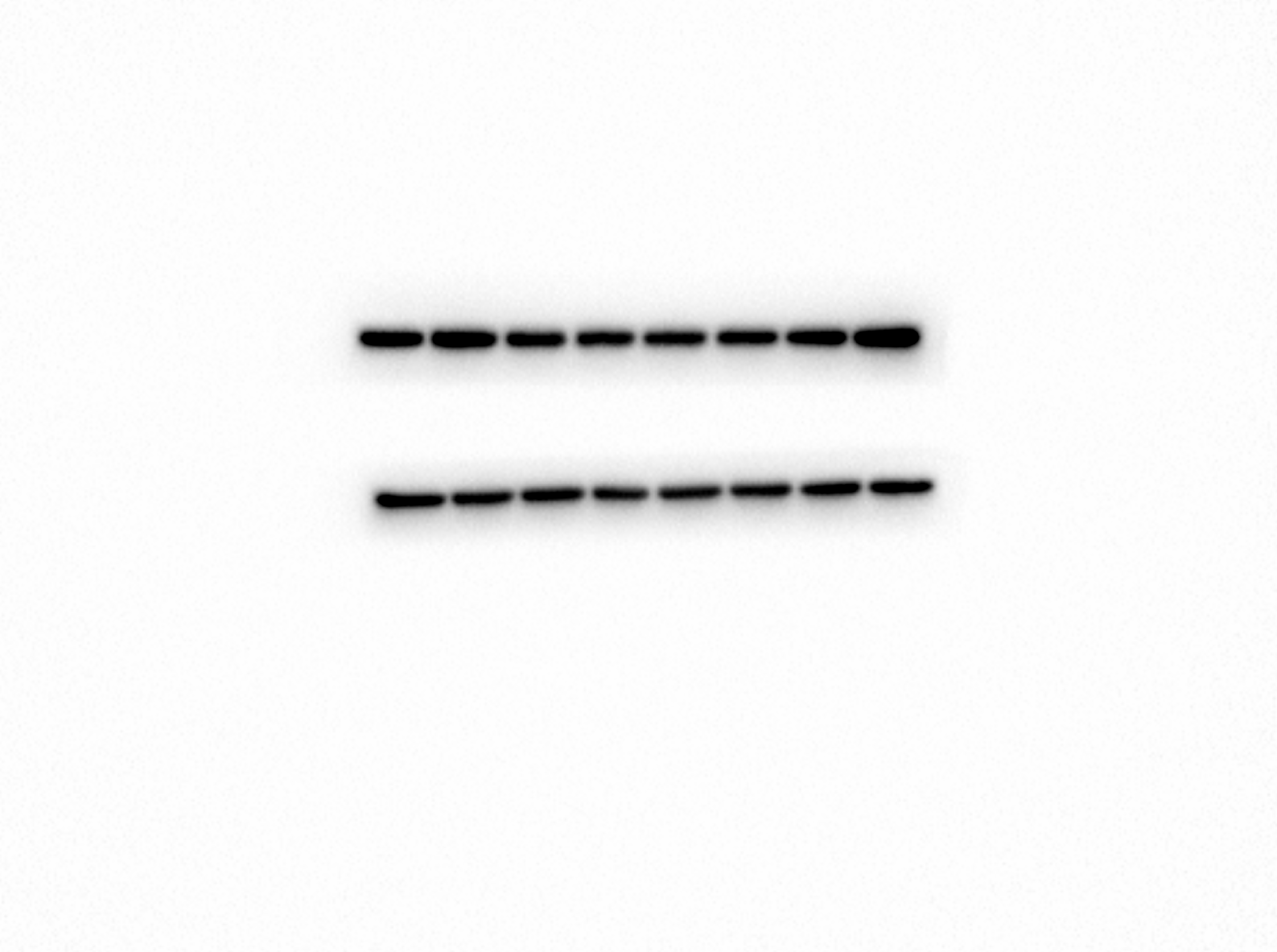

Supplement: Figure 4—source data 1. [file elife-83083-fig4-data1.zip › Figure 4-source data/Figure 4F a┬-Tubulin.tif]

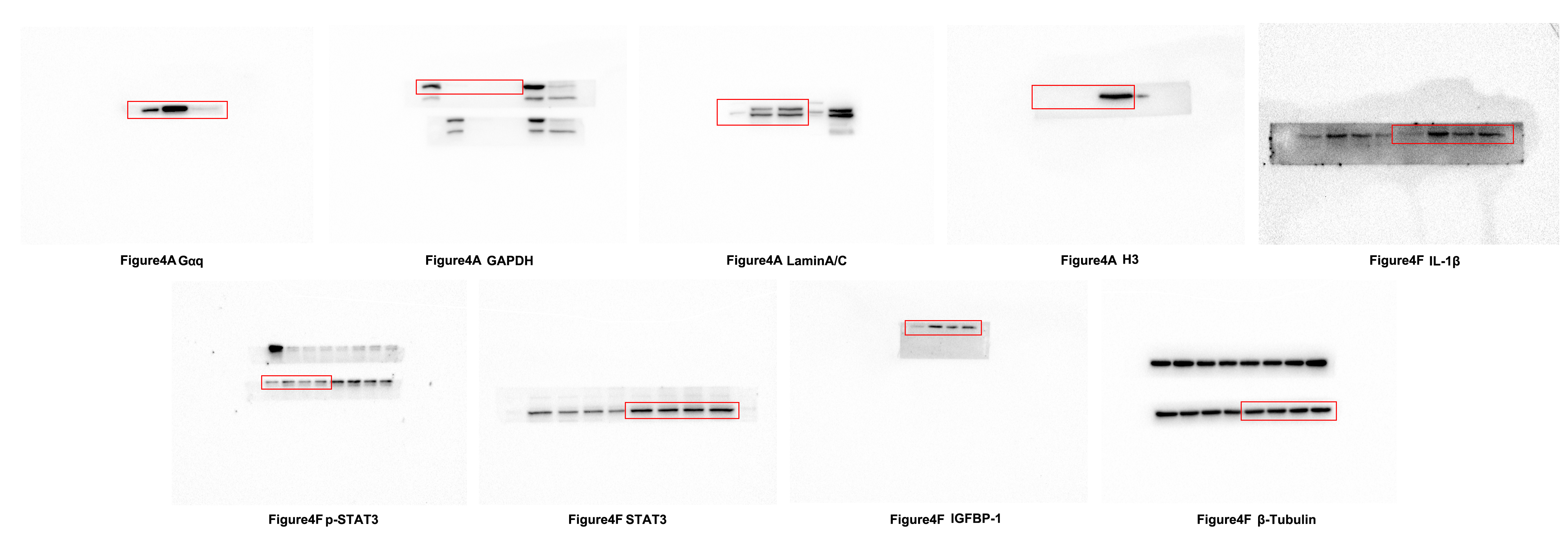

Supplement: Figure 4—source data 1. [file elife-83083-fig4-data1.zip › Figure 4-source data/Figure 4-source data.jpg]

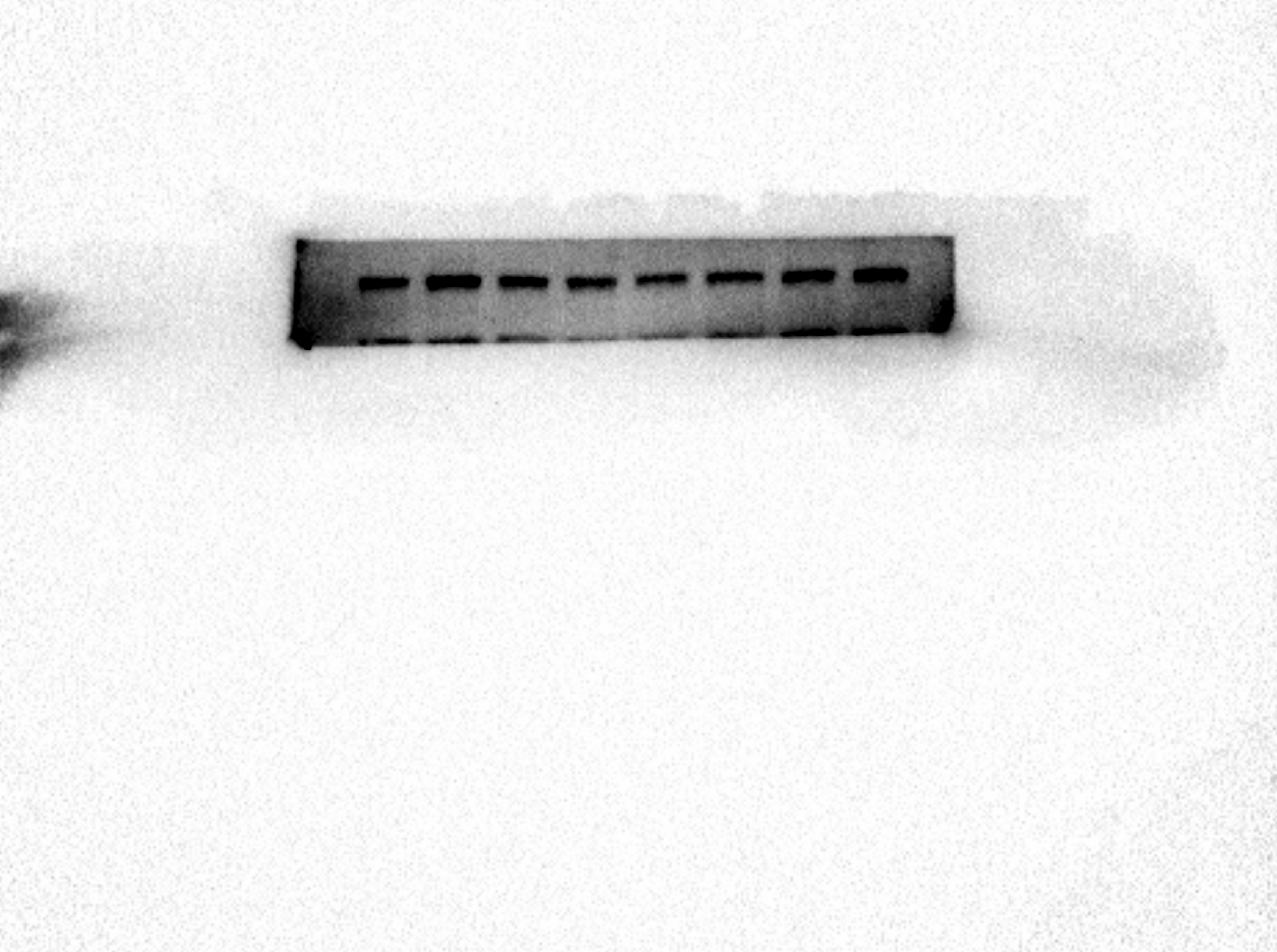

Supplement: Figure 5—source data 1. [file elife-83083-fig5-data1.zip › Figure 5-source data/Figure 5A HDAC5.tif]

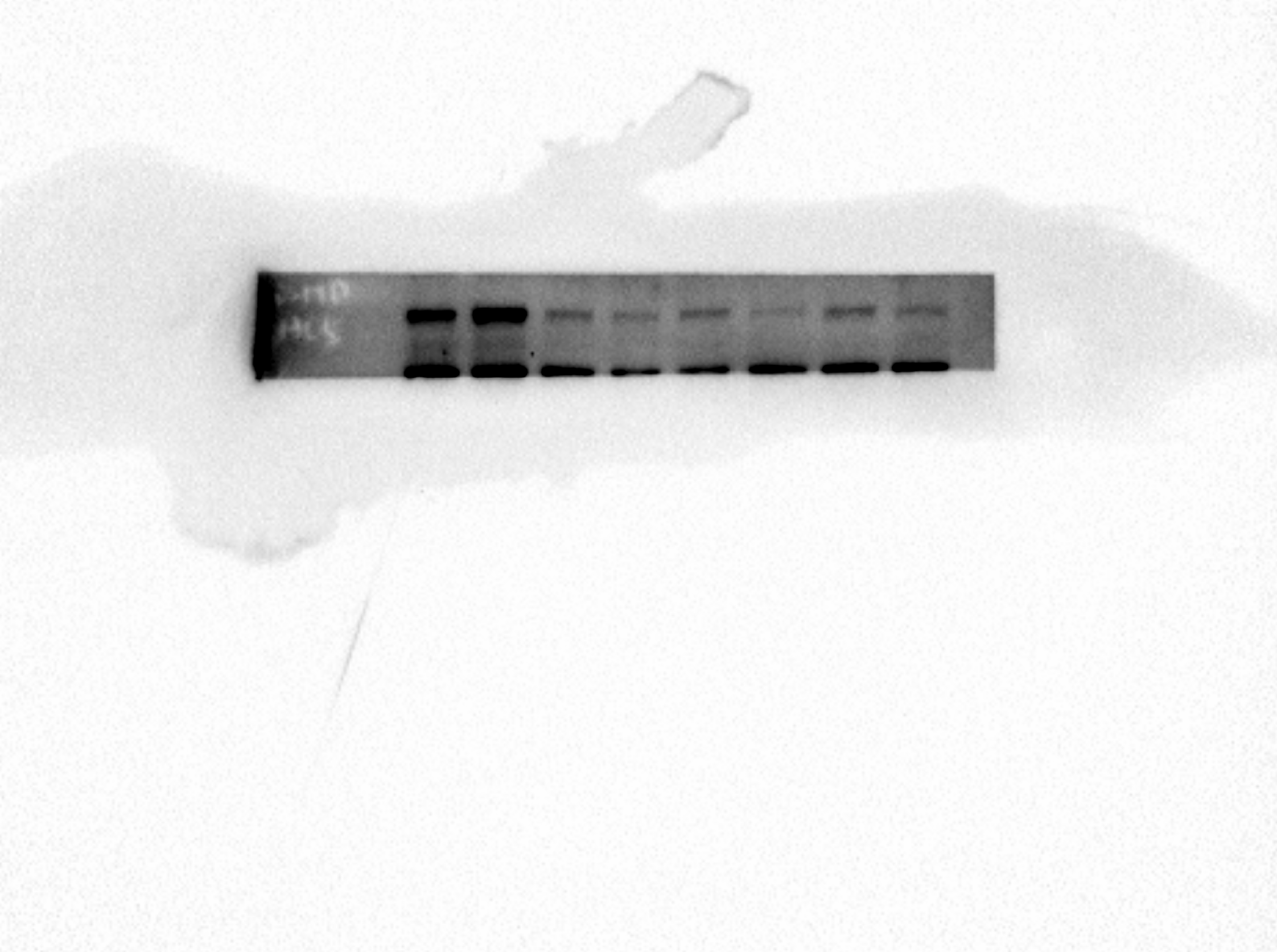

Supplement: Figure 5—source data 1. [file elife-83083-fig5-data1.zip › Figure 5-source data/Figure 5A p-HDAC5.tif]

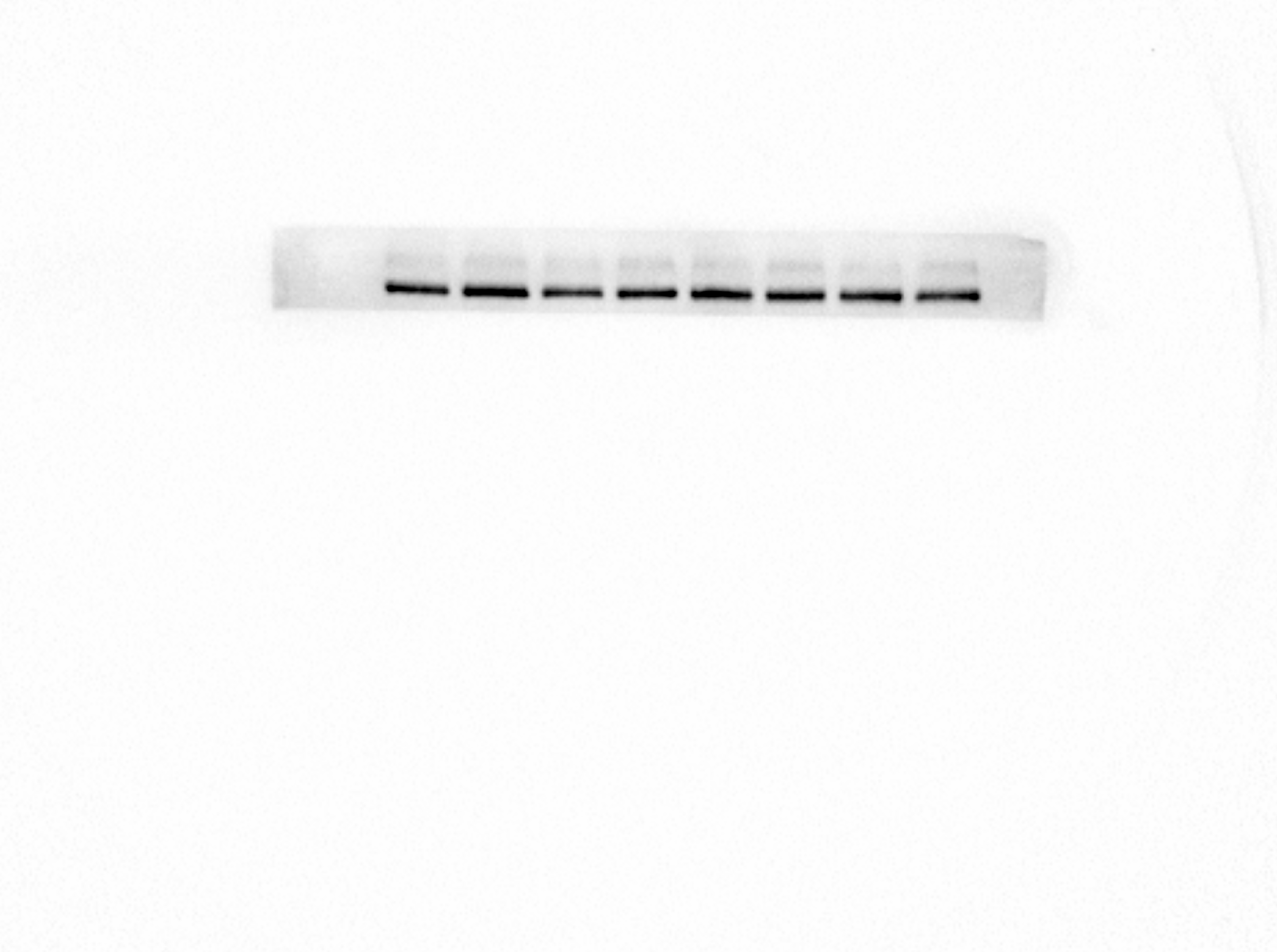

Supplement: Figure 5—source data 1. [file elife-83083-fig5-data1.zip › Figure 5-source data/Figure 5A PKDPKCu.tif]

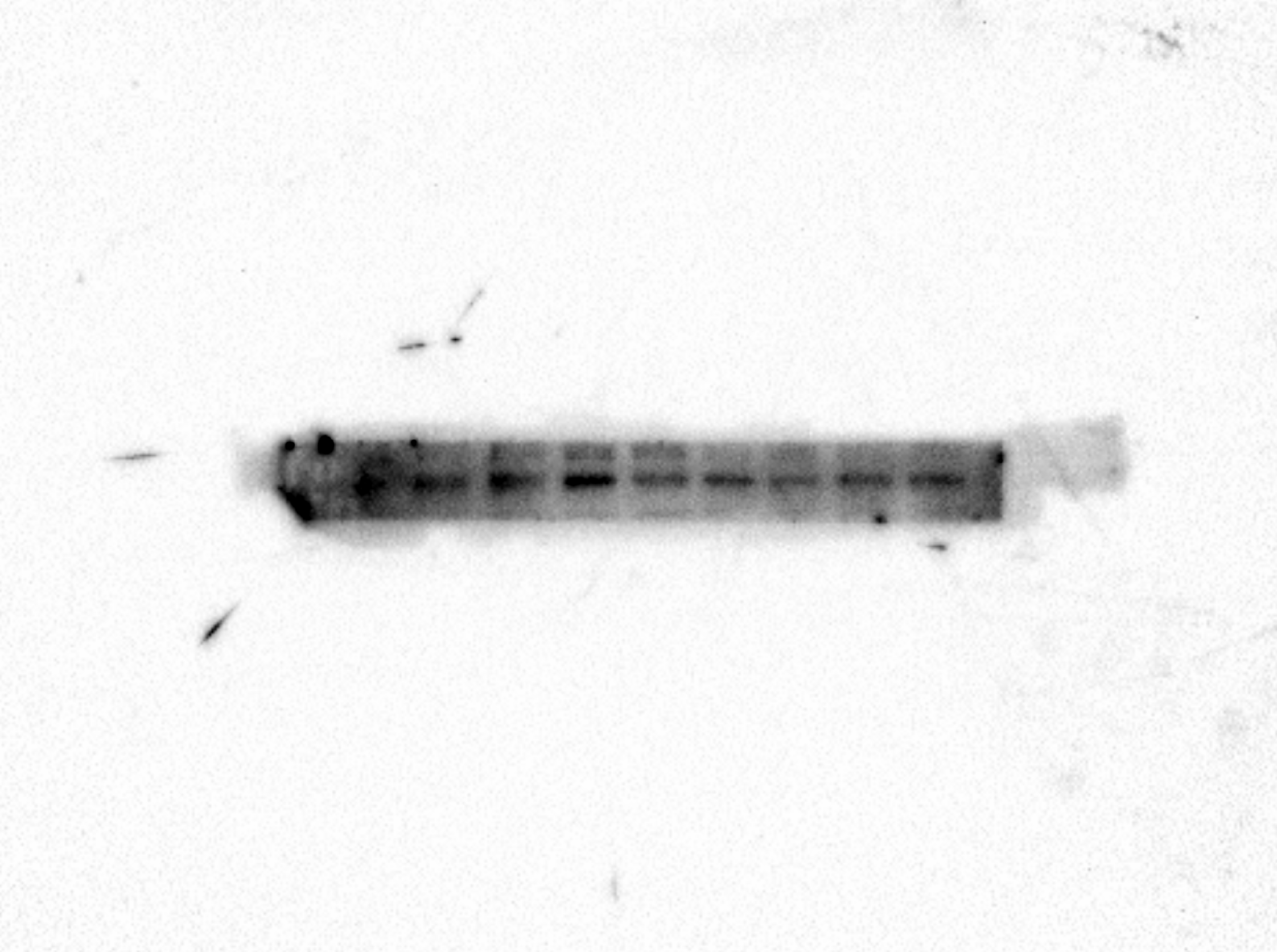

Supplement: Figure 5—source data 1. [file elife-83083-fig5-data1.zip › Figure 5-source data/Figure 5A p-PKDPKCa╠.tif]

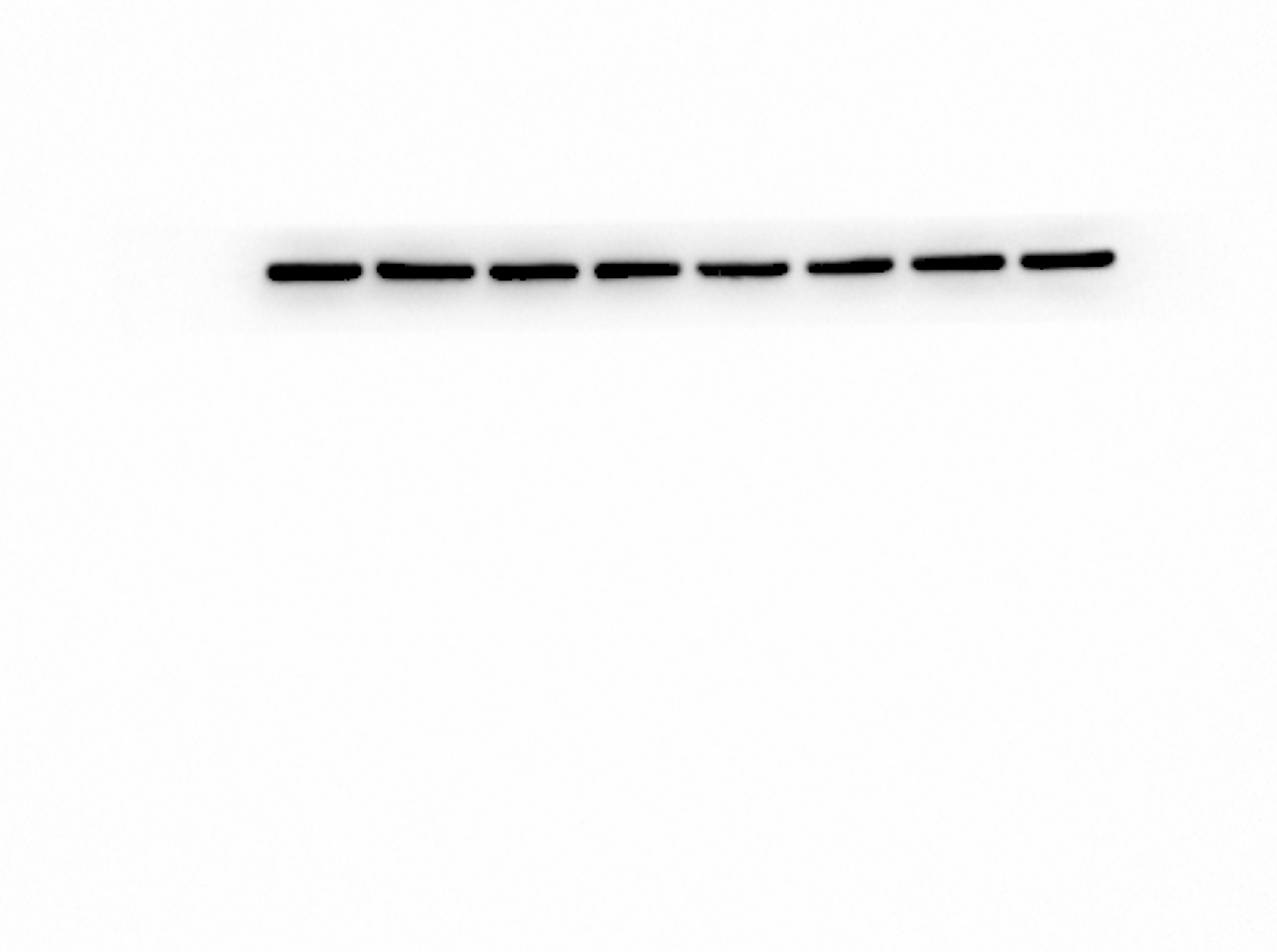

Supplement: Figure 5—source data 1. [file elife-83083-fig5-data1.zip › Figure 5-source data/Figure 5A a┬-Tubulin.tif]

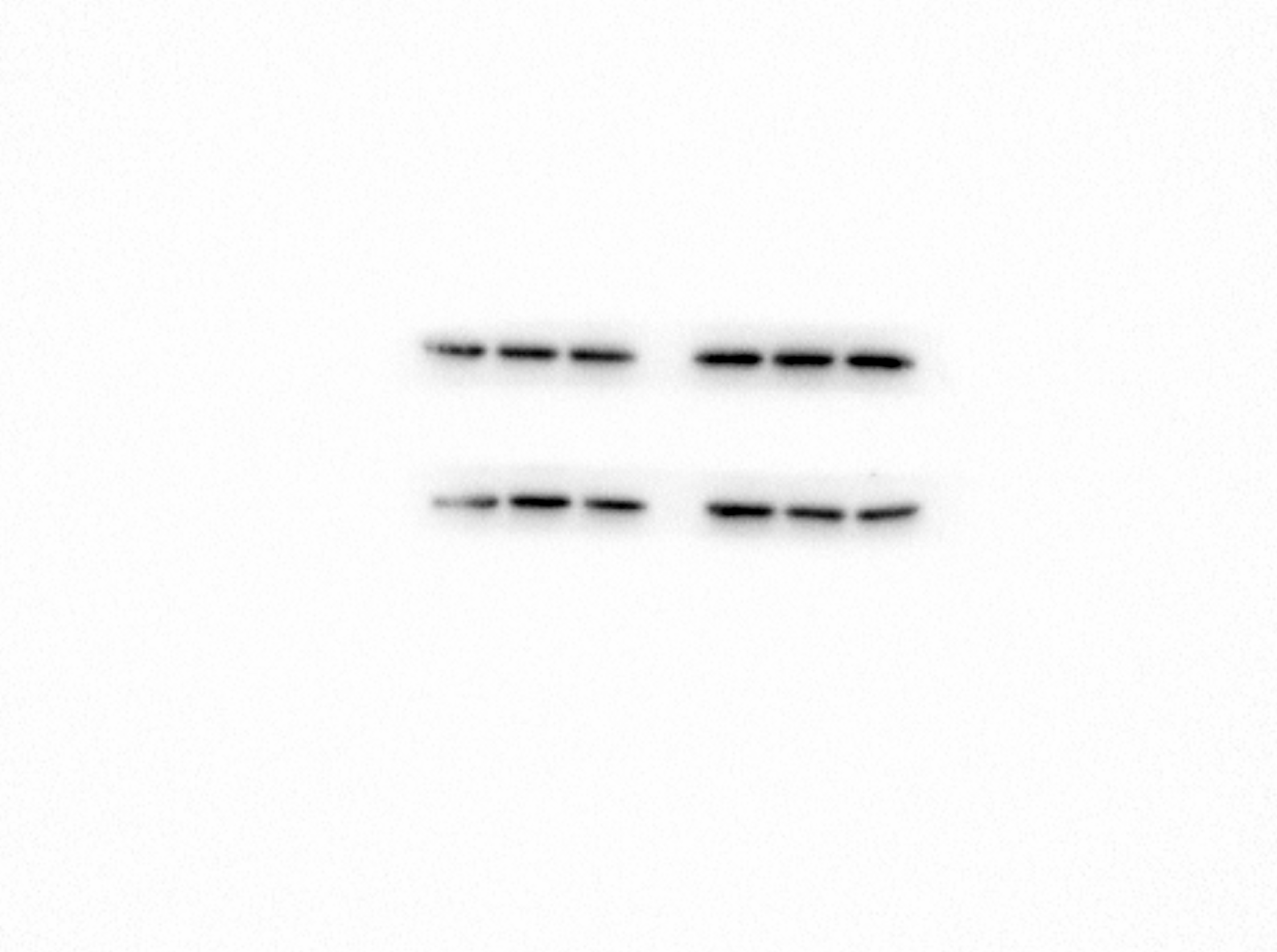

Supplement: Figure 5—source data 1. [file elife-83083-fig5-data1.zip › Figure 5-source data/Figure 5B GAPDH.tif]

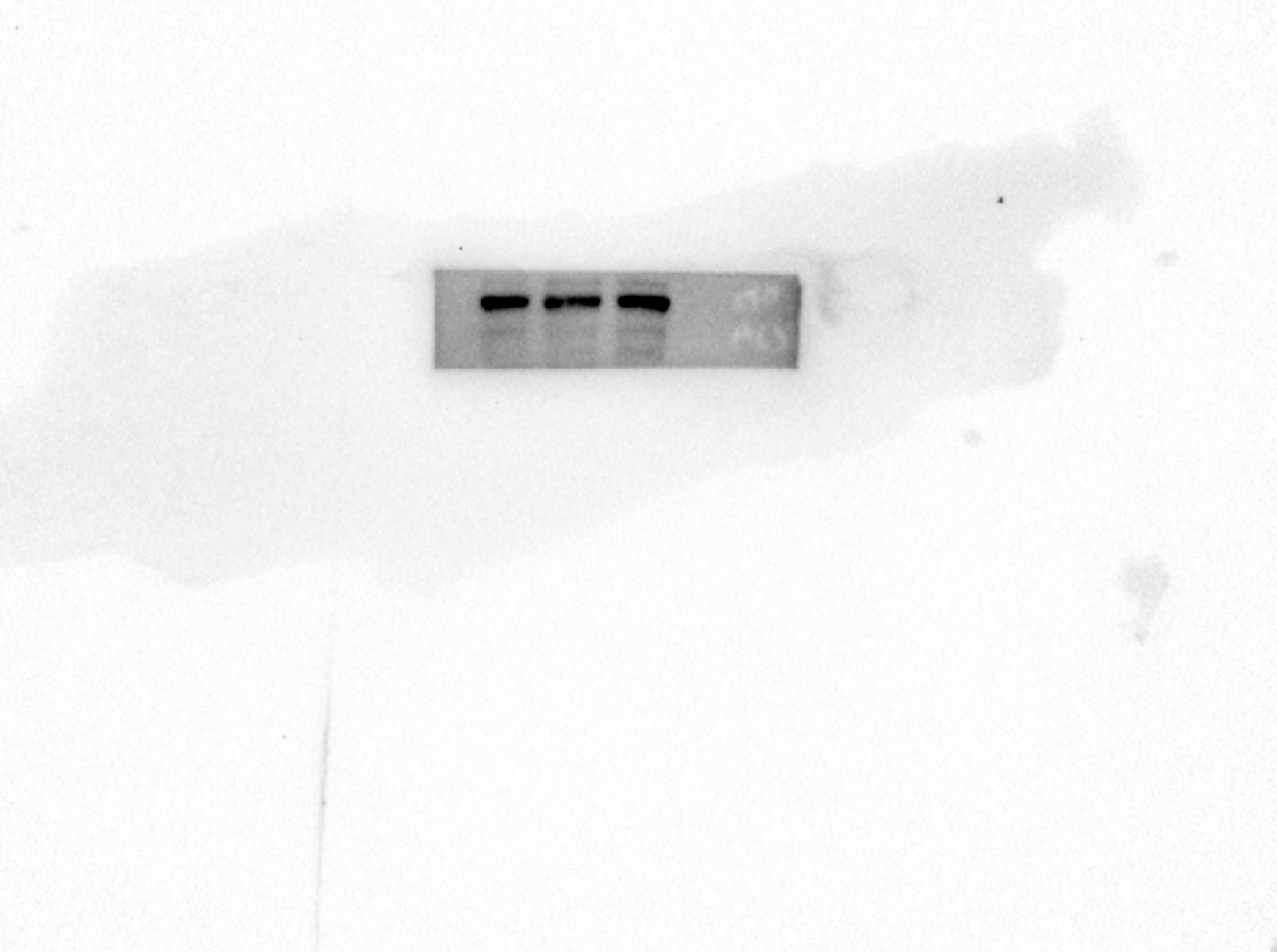

Supplement: Figure 5—source data 1. [file elife-83083-fig5-data1.zip › Figure 5-source data/Figure 5B HDAC5.tif]

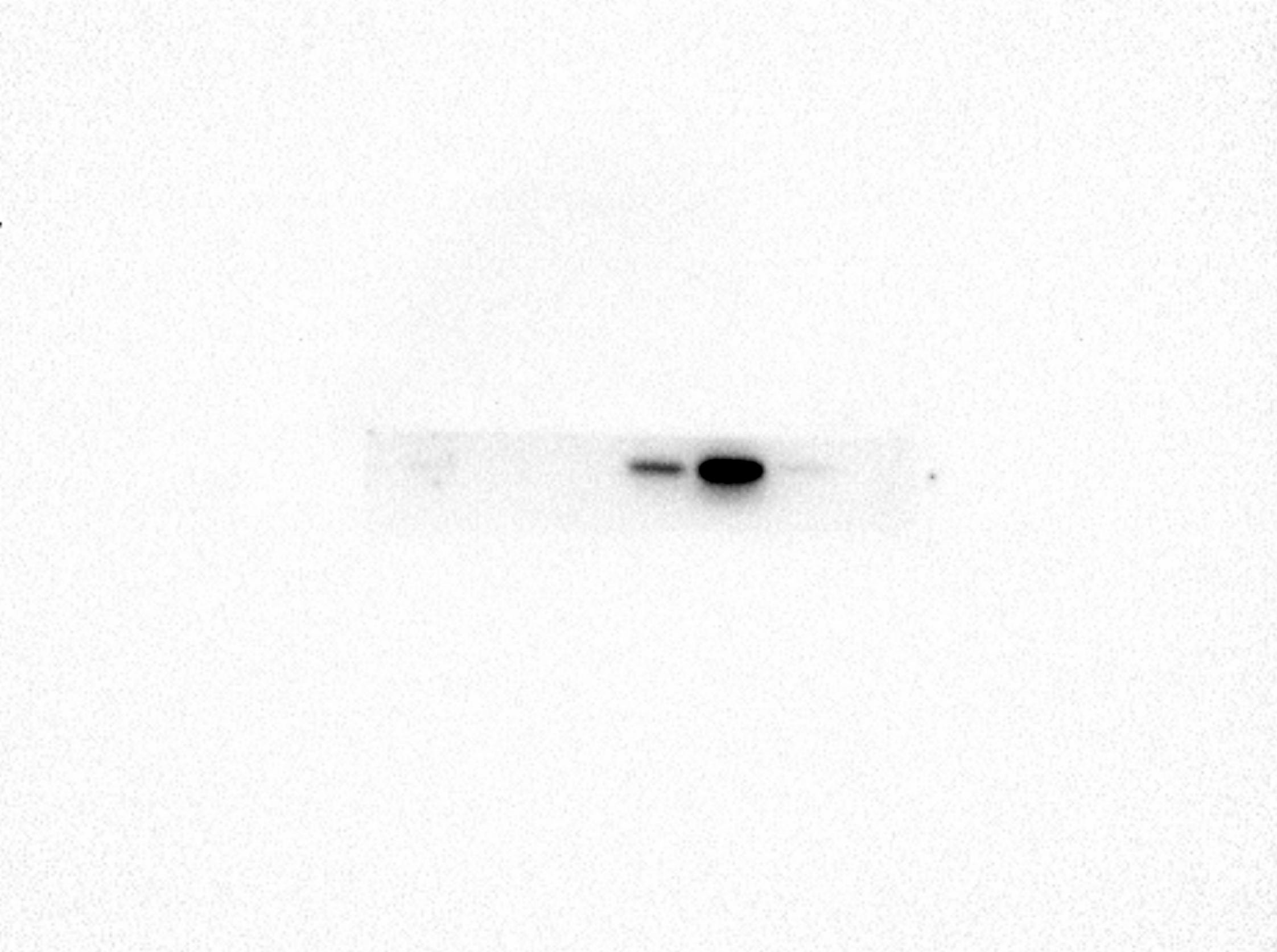

Supplement: Figure 5—source data 1. [file elife-83083-fig5-data1.zip › Figure 5-source data/Figure 5B IGFBP-1.tif]

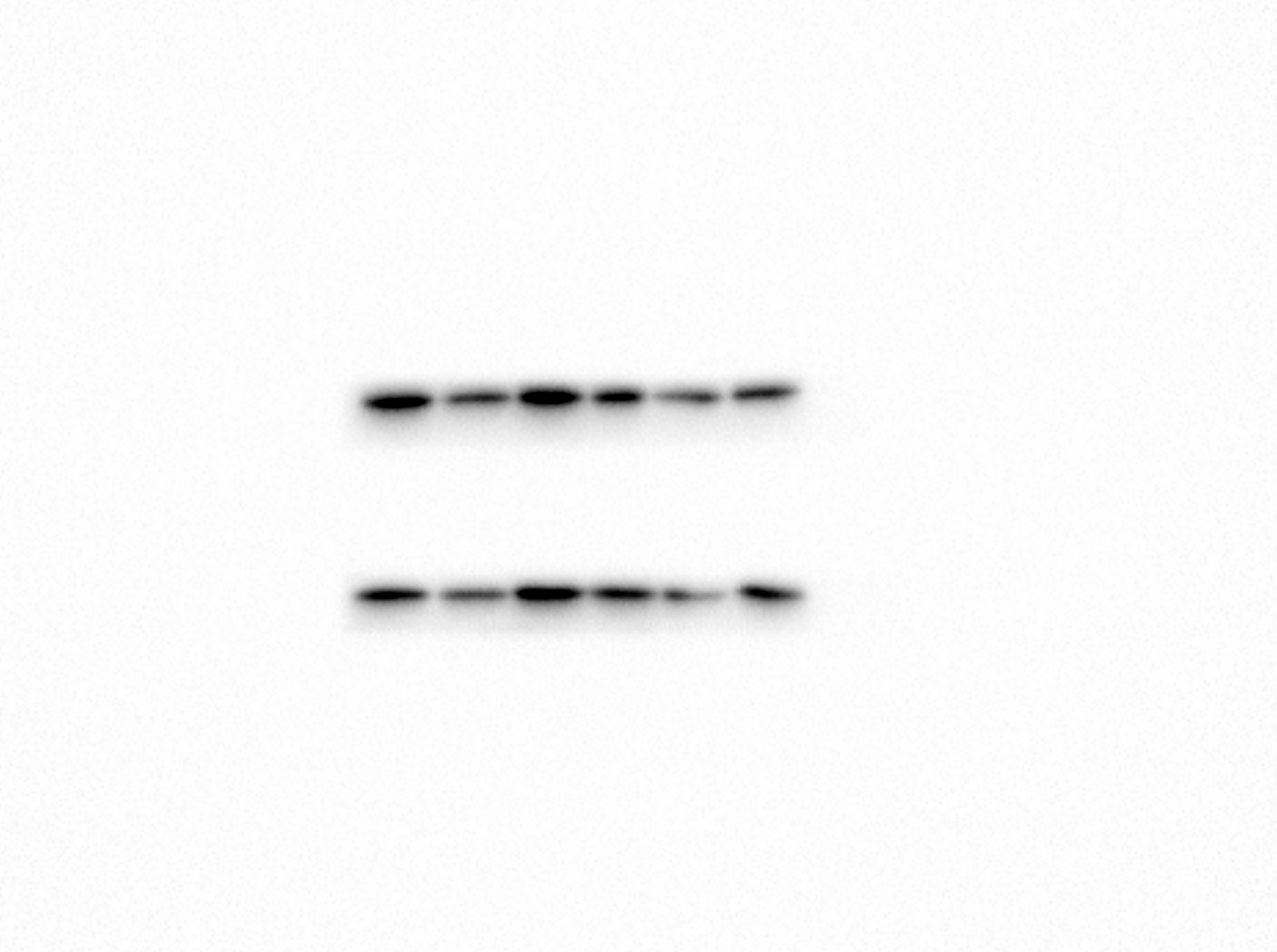

Supplement: Figure 5—source data 1. [file elife-83083-fig5-data1.zip › Figure 5-source data/Figure 5B Ia╩Ba┴.tif]

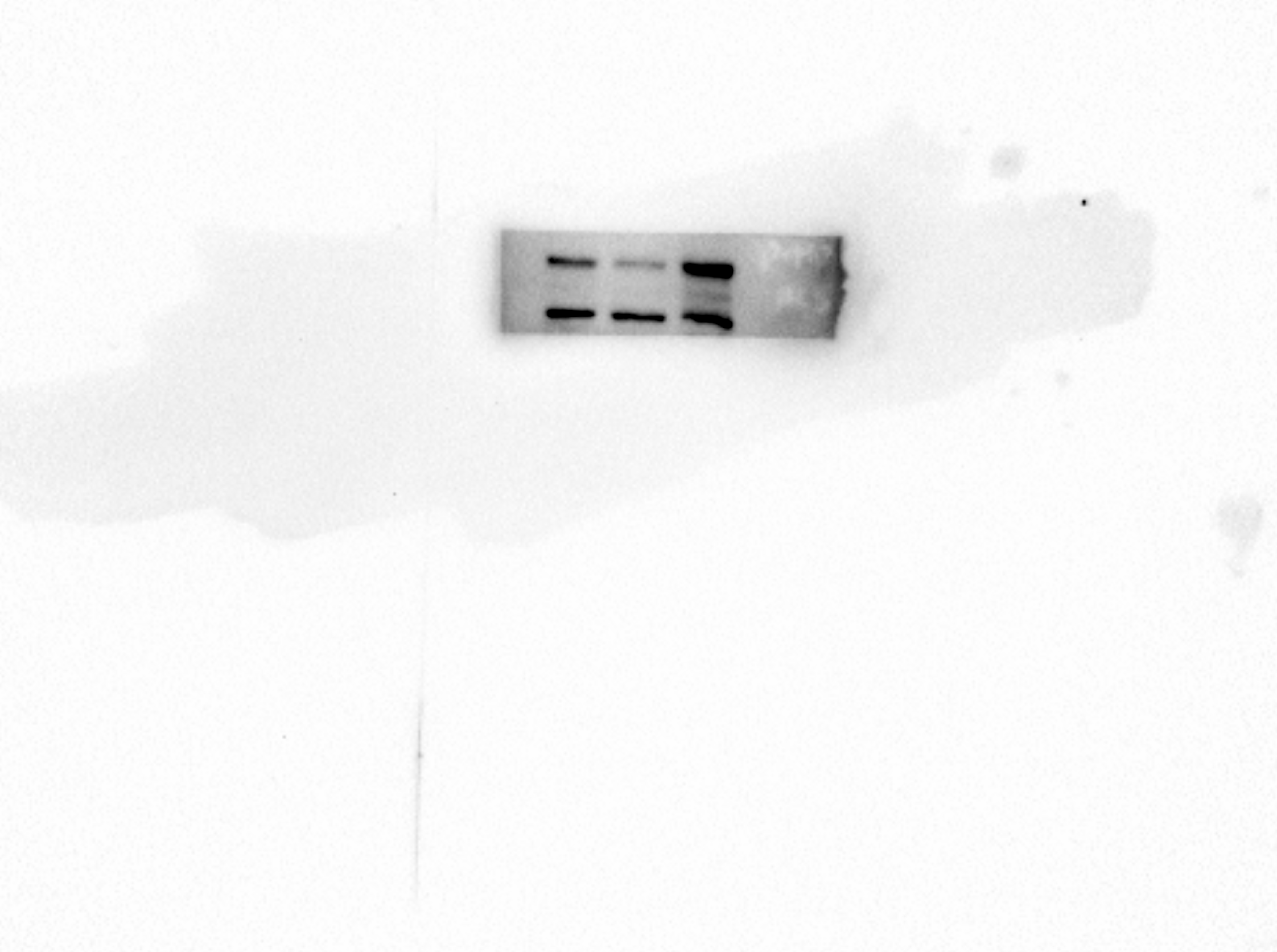

Supplement: Figure 5—source data 1. [file elife-83083-fig5-data1.zip › Figure 5-source data/Figure 5B p-HDAC5.tif]

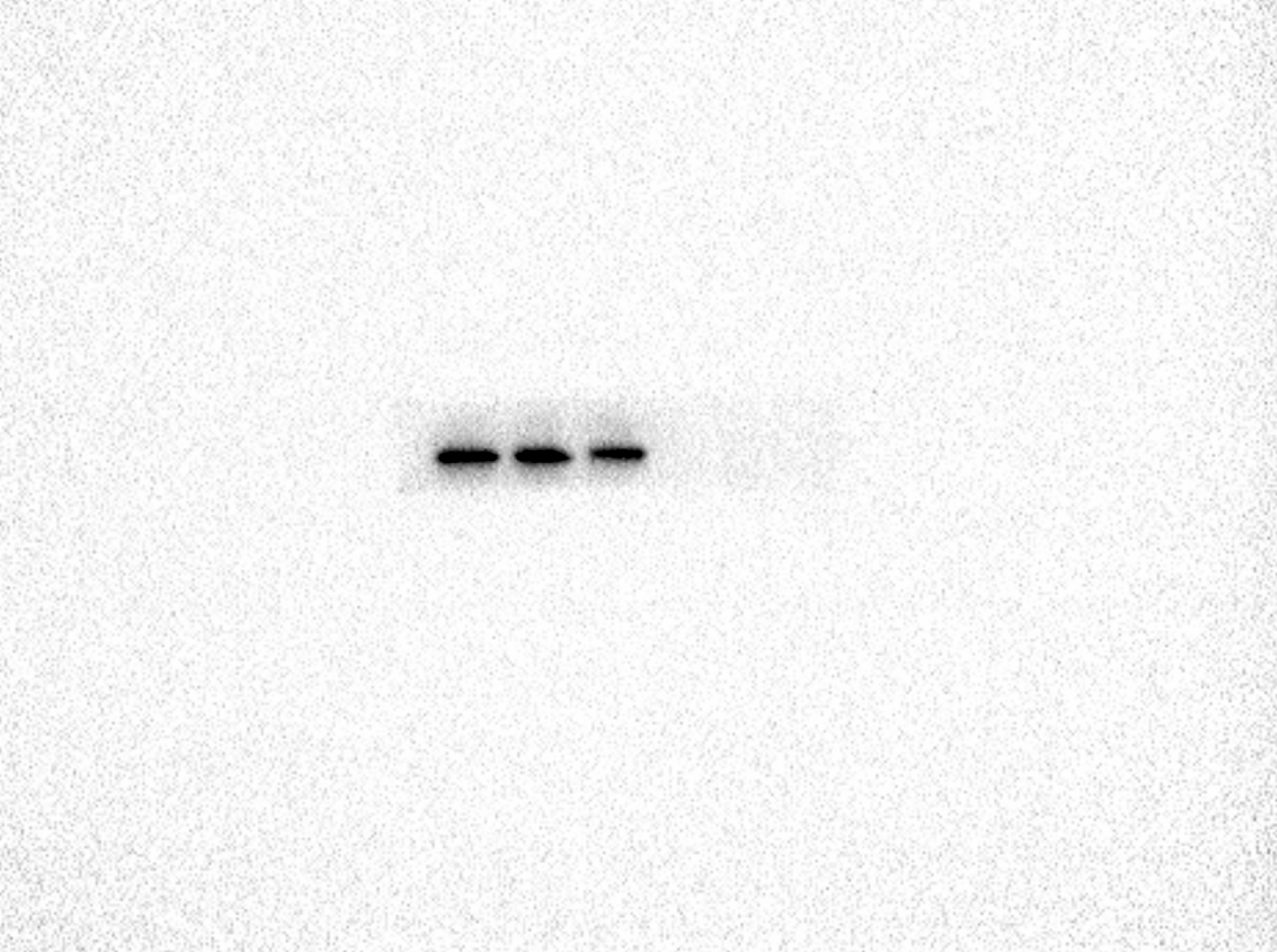

Supplement: Figure 5—source data 1. [file elife-83083-fig5-data1.zip › Figure 5-source data/Figure 5B PKDPKCa╠.tif]

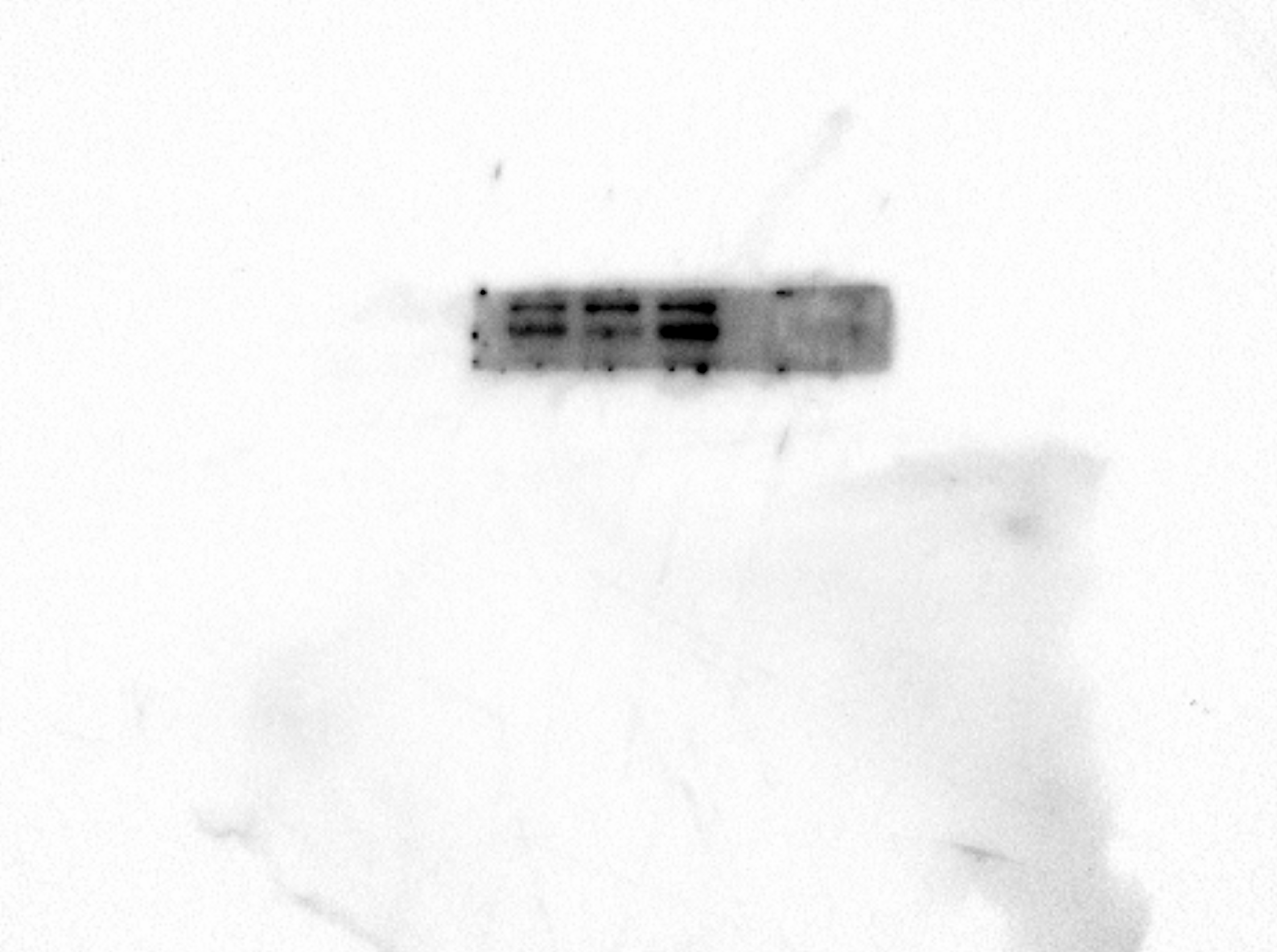

Supplement: Figure 5—source data 1. [file elife-83083-fig5-data1.zip › Figure 5-source data/Figure 5B p-PKDPKCa╠.tif]

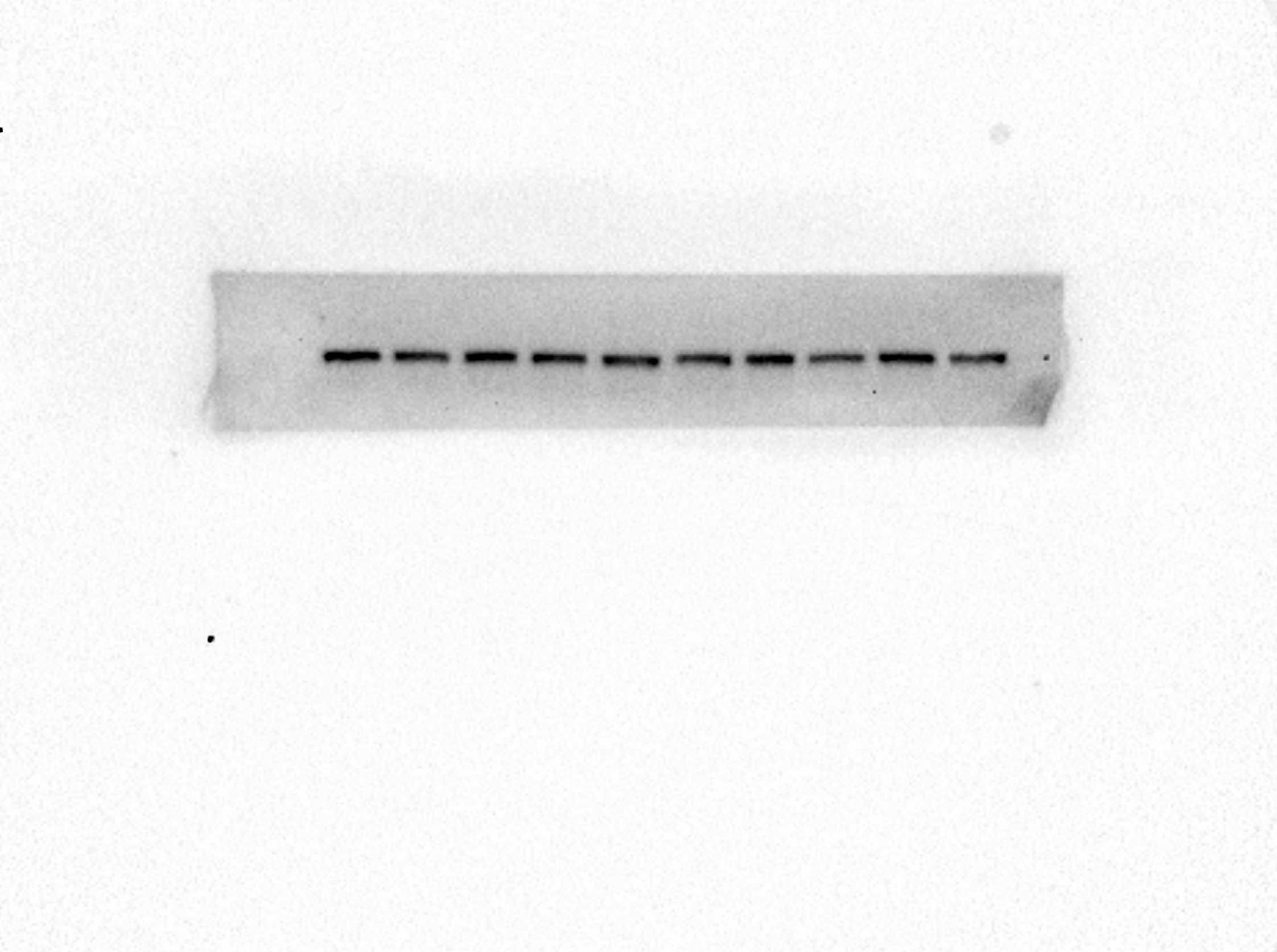

Supplement: Figure 5—source data 1. [file elife-83083-fig5-data1.zip › Figure 5-source data/Figure 5C HDAC5.tif]

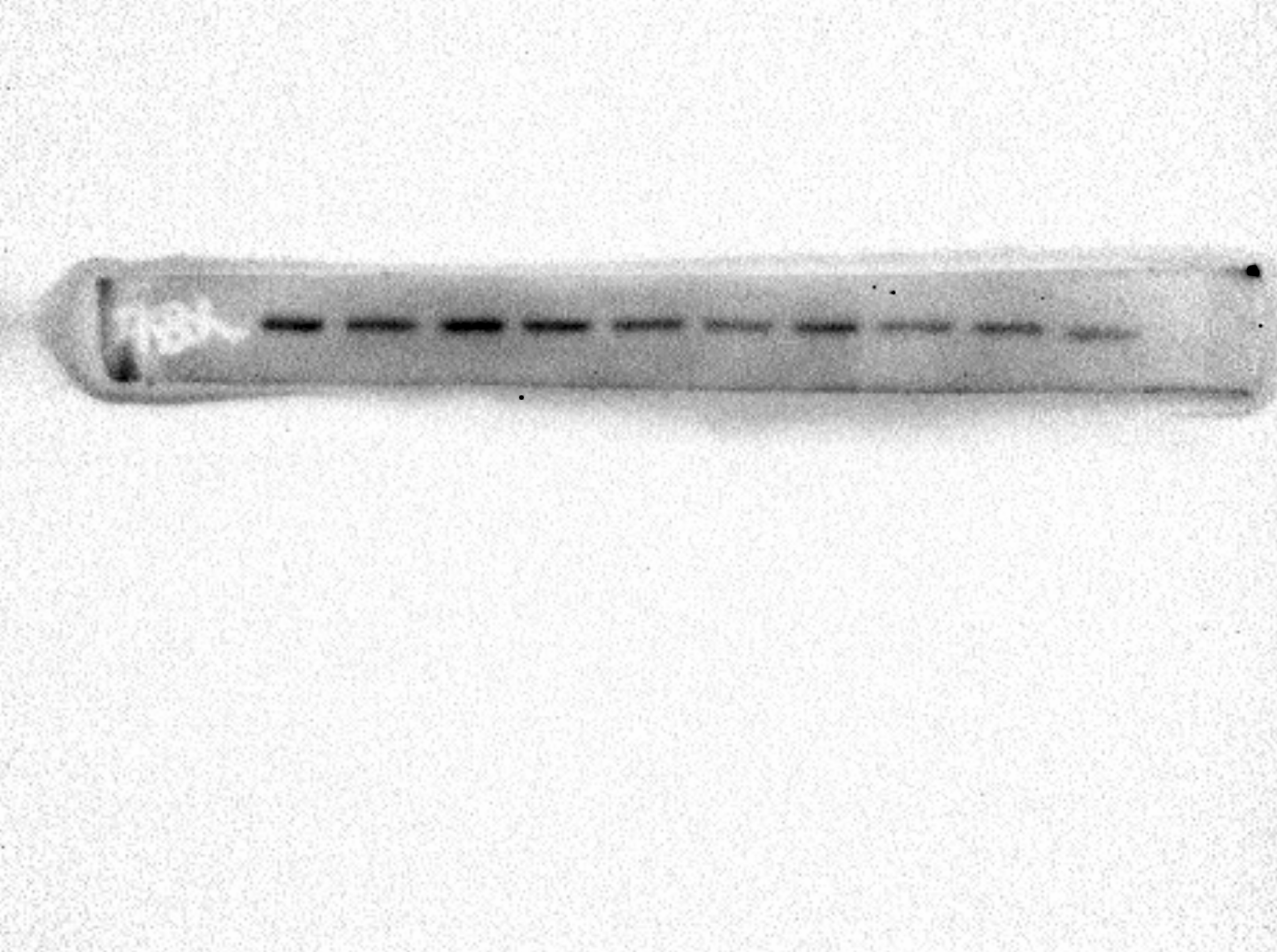

Supplement: Figure 5—source data 1. [file elife-83083-fig5-data1.zip › Figure 5-source data/Figure 5C Ia╩Ba┴.tif]

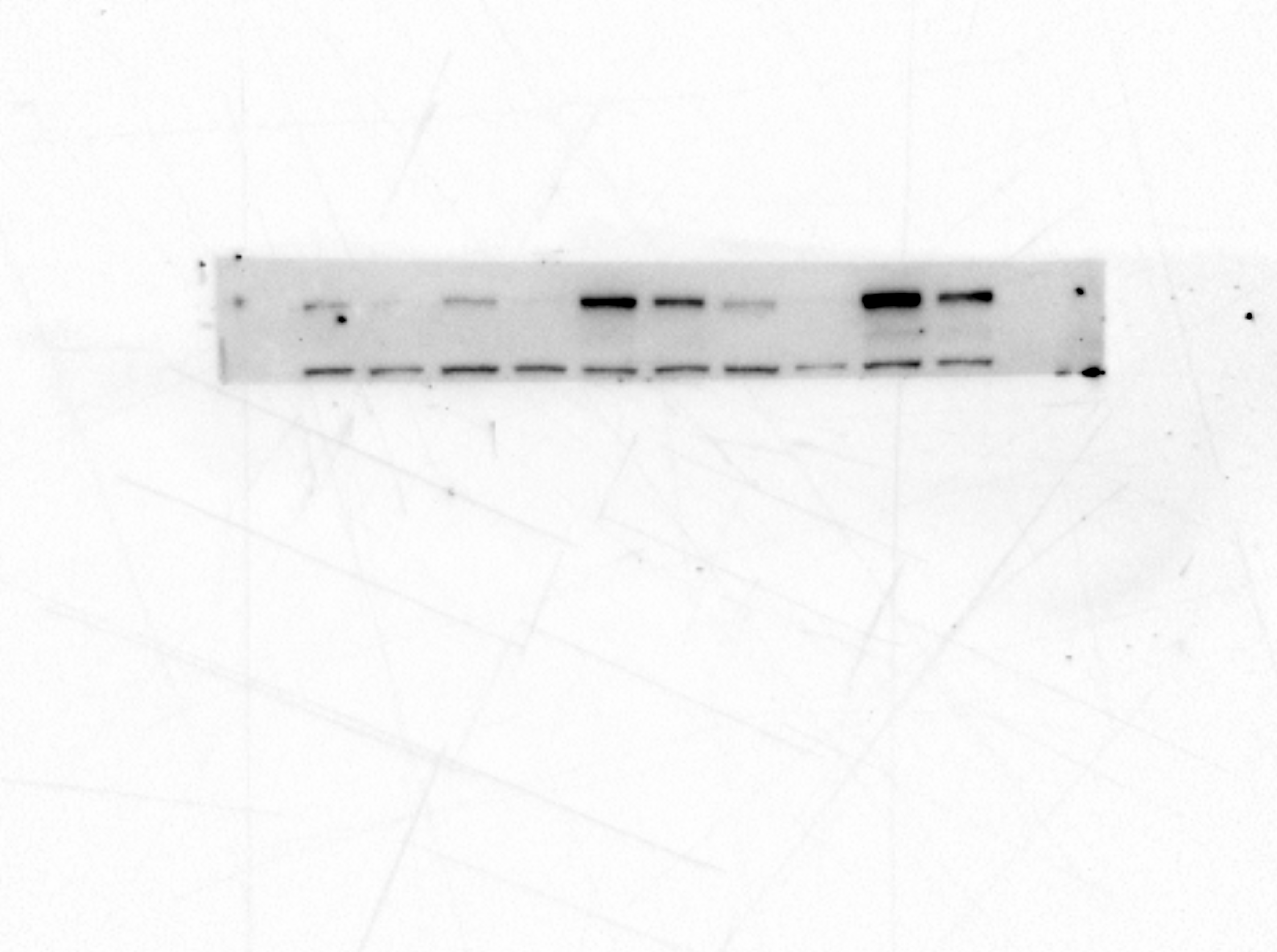

Supplement: Figure 5—source data 1. [file elife-83083-fig5-data1.zip › Figure 5-source data/Figure 5C p-HDAC5.tif]

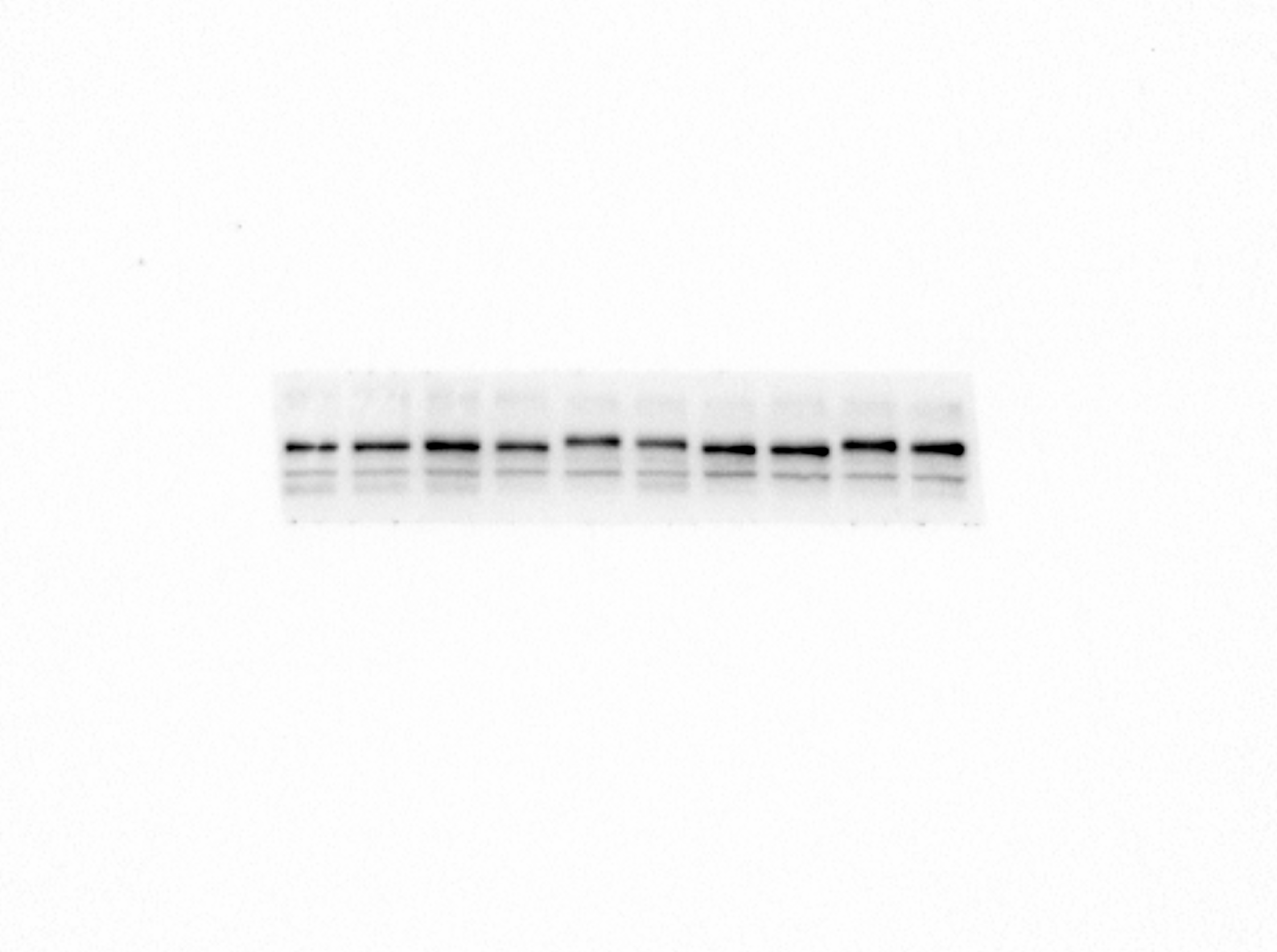

Supplement: Figure 5—source data 1. [file elife-83083-fig5-data1.zip › Figure 5-source data/Figure 5C PKDPKCa╠.tif]

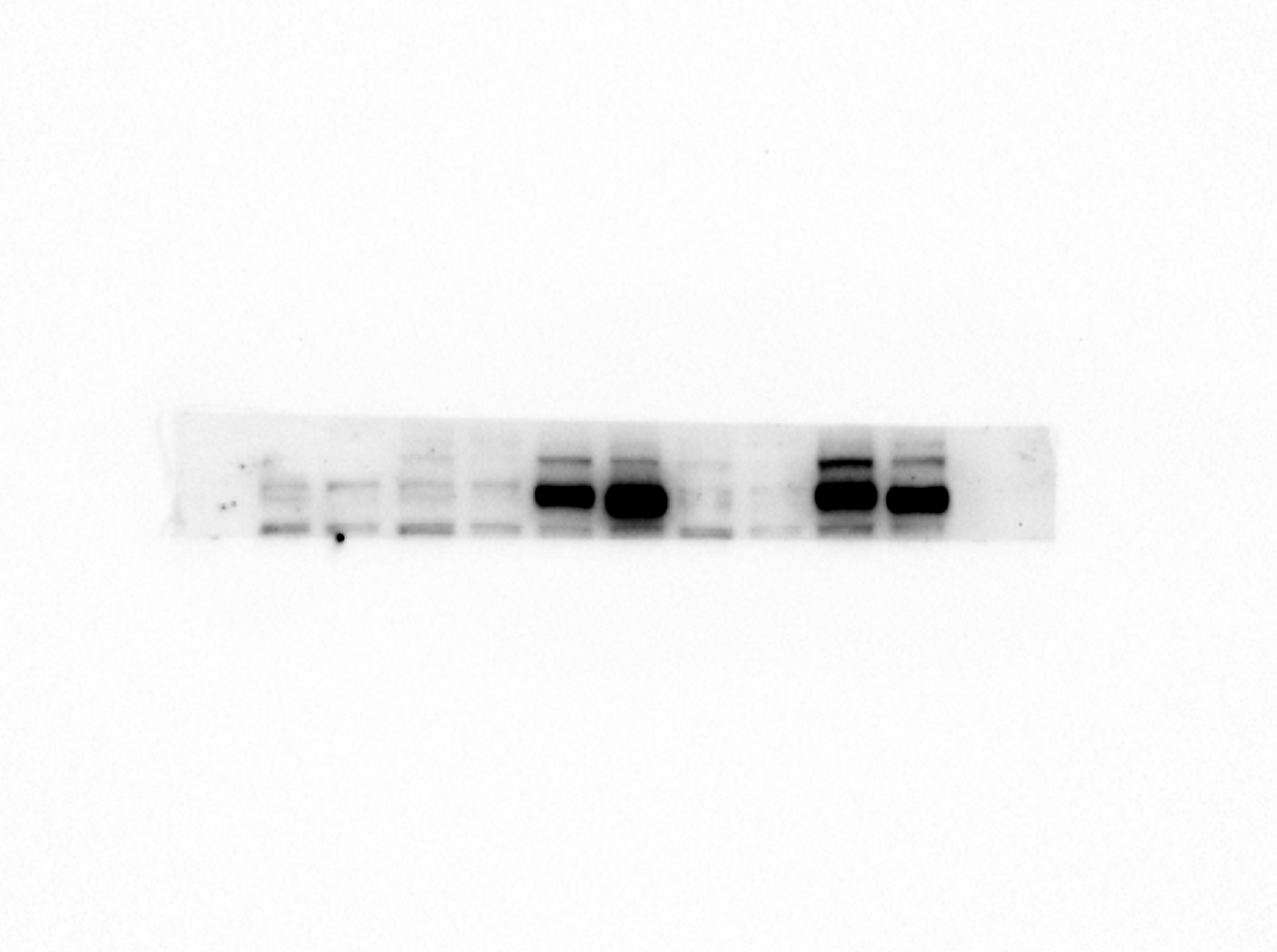

Supplement: Figure 5—source data 1. [file elife-83083-fig5-data1.zip › Figure 5-source data/Figure 5C p-PKDPKCa╠.tif]

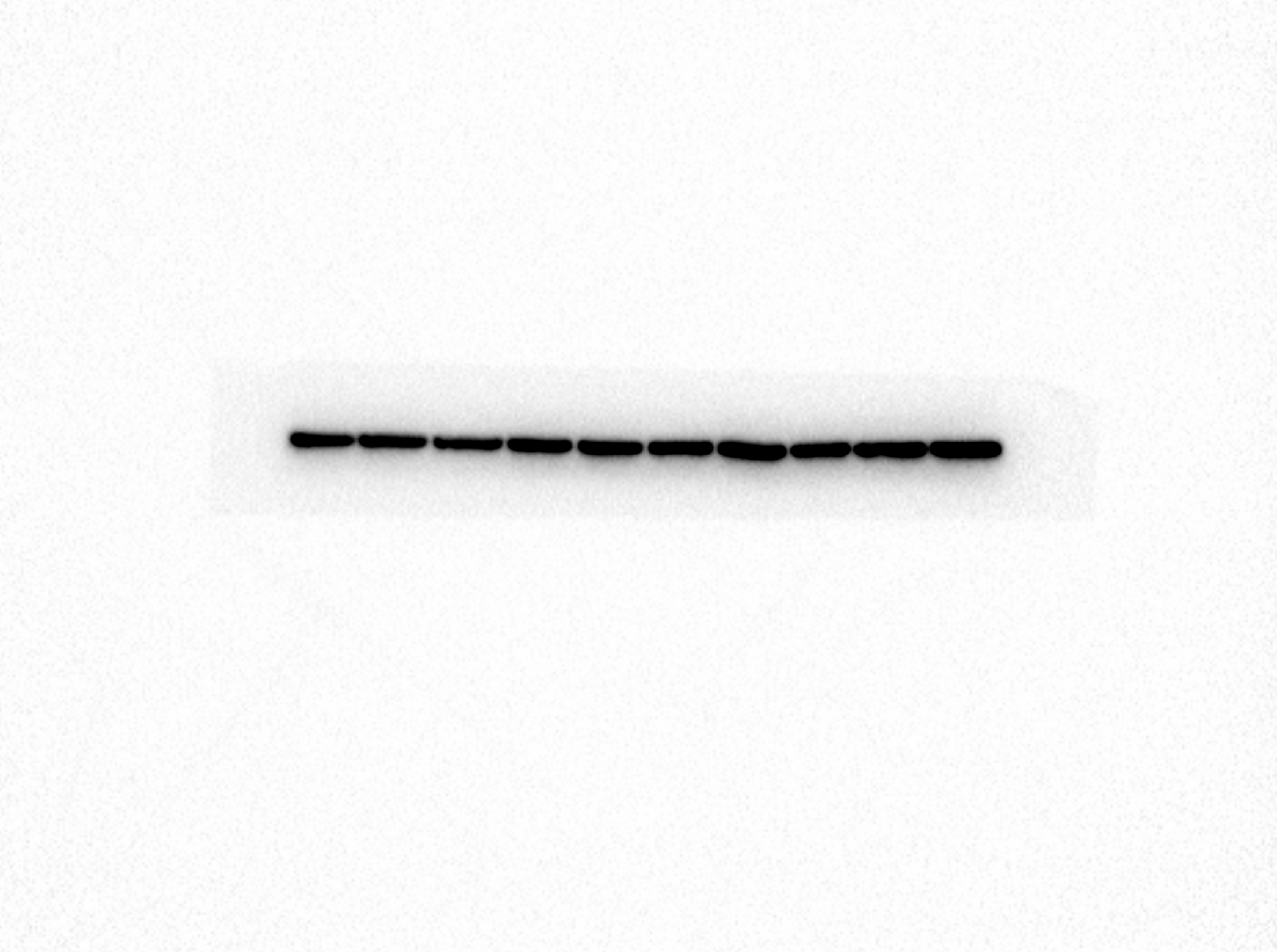

Supplement: Figure 5—source data 1. [file elife-83083-fig5-data1.zip › Figure 5-source data/Figure 5C a┬-Tubulin.tif]

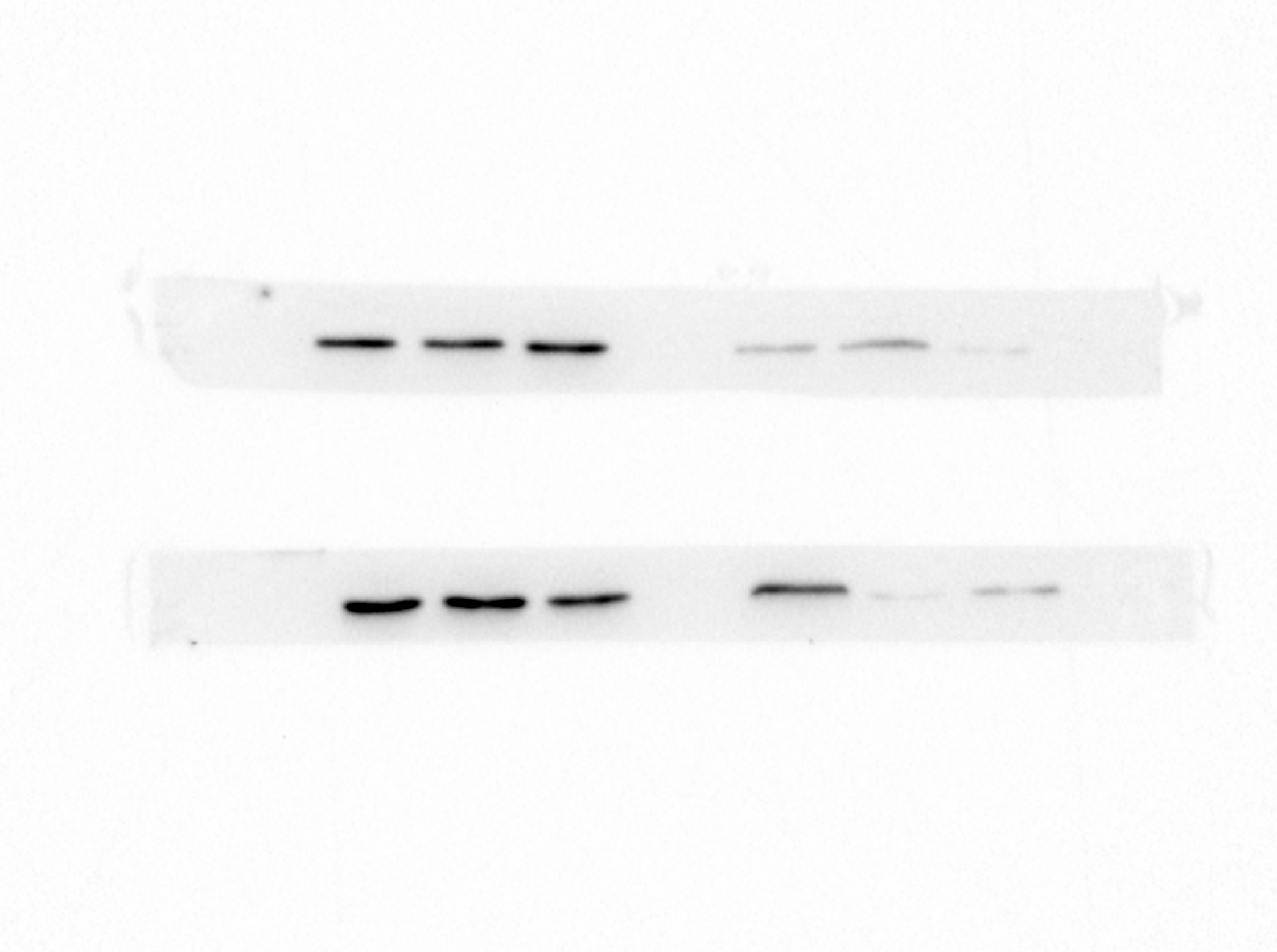

Supplement: Figure 5—source data 1. [file elife-83083-fig5-data1.zip › Figure 5-source data/Figure 5E GAPDH.tif]

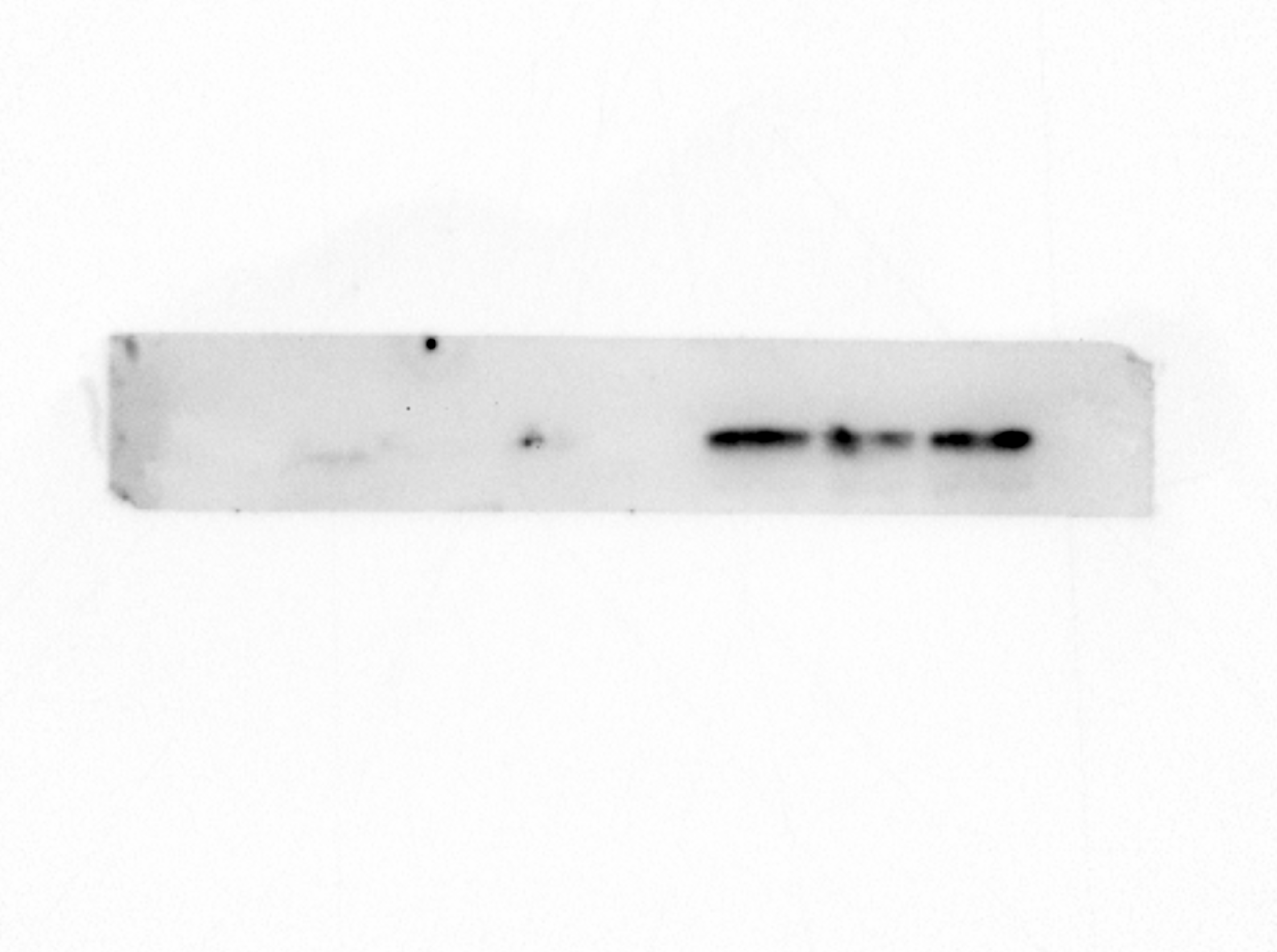

Supplement: Figure 5—source data 1. [file elife-83083-fig5-data1.zip › Figure 5-source data/Figure 5E H3.tif]

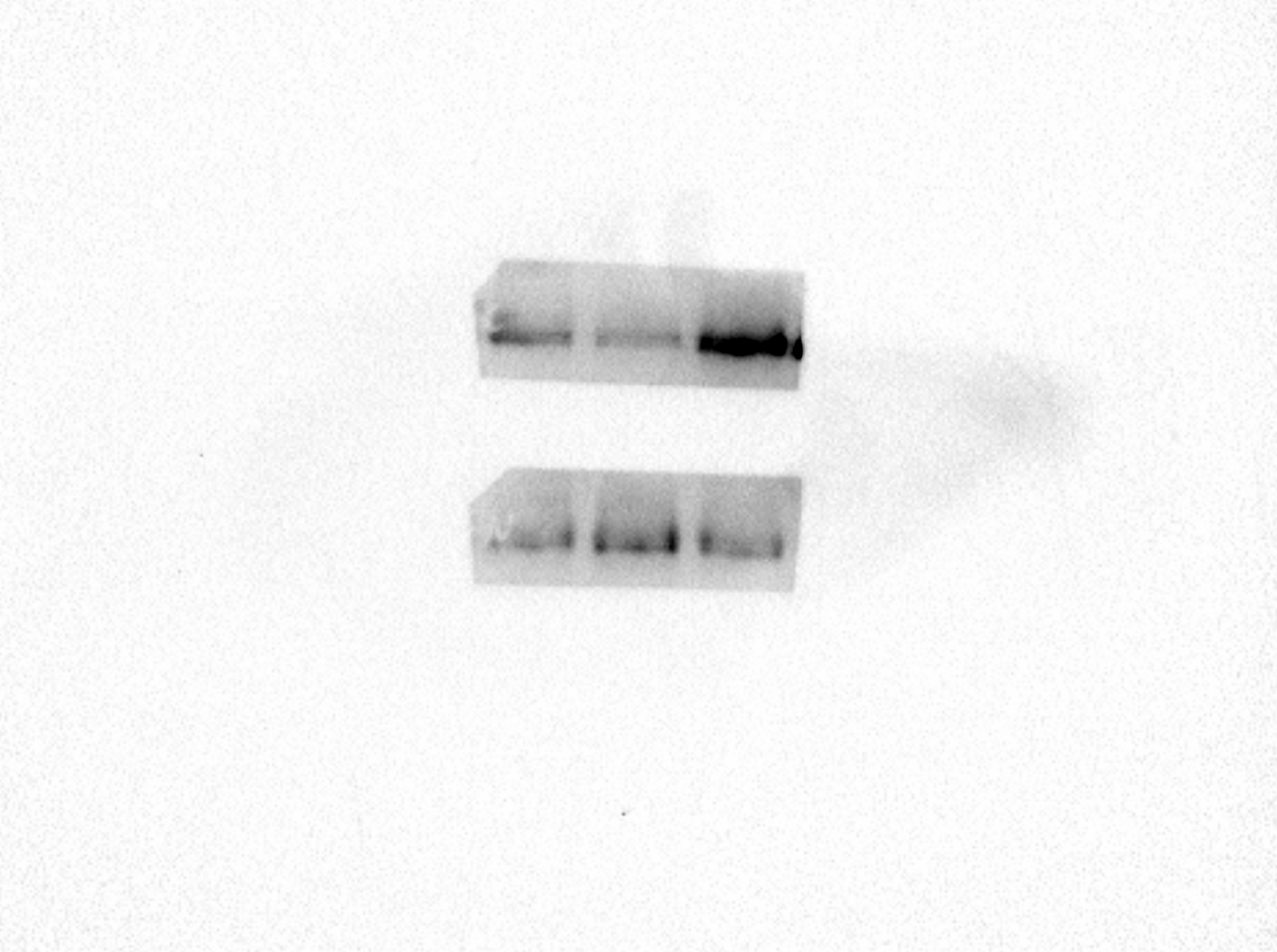

Supplement: Figure 5—source data 1. [file elife-83083-fig5-data1.zip › Figure 5-source data/Figure 5E p-HDAC5.tif]

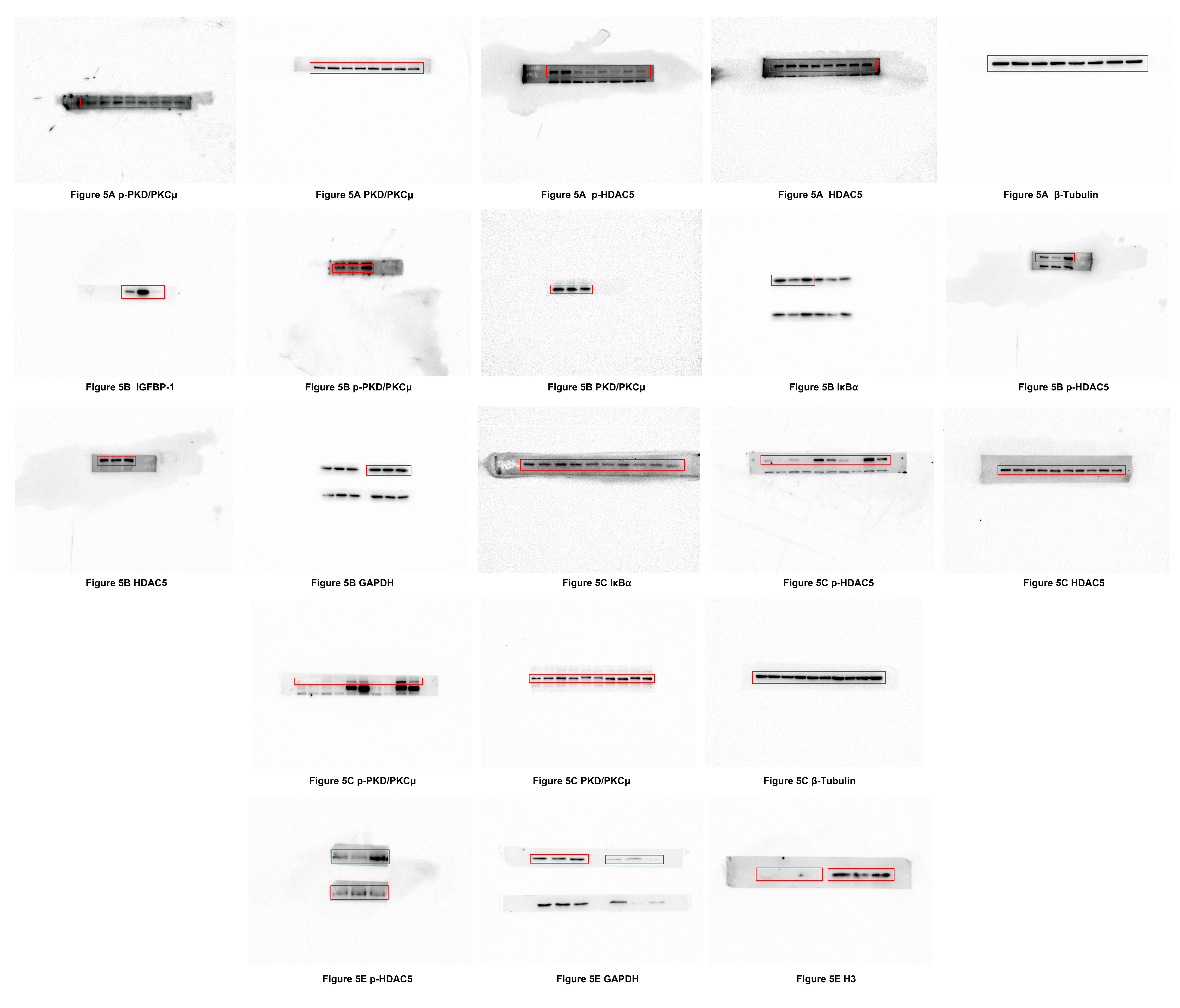

Supplement: Figure 5—source data 1. [file elife-83083-fig5-data1.zip › Figure 5-source data/Figure 5-source data.jpg]

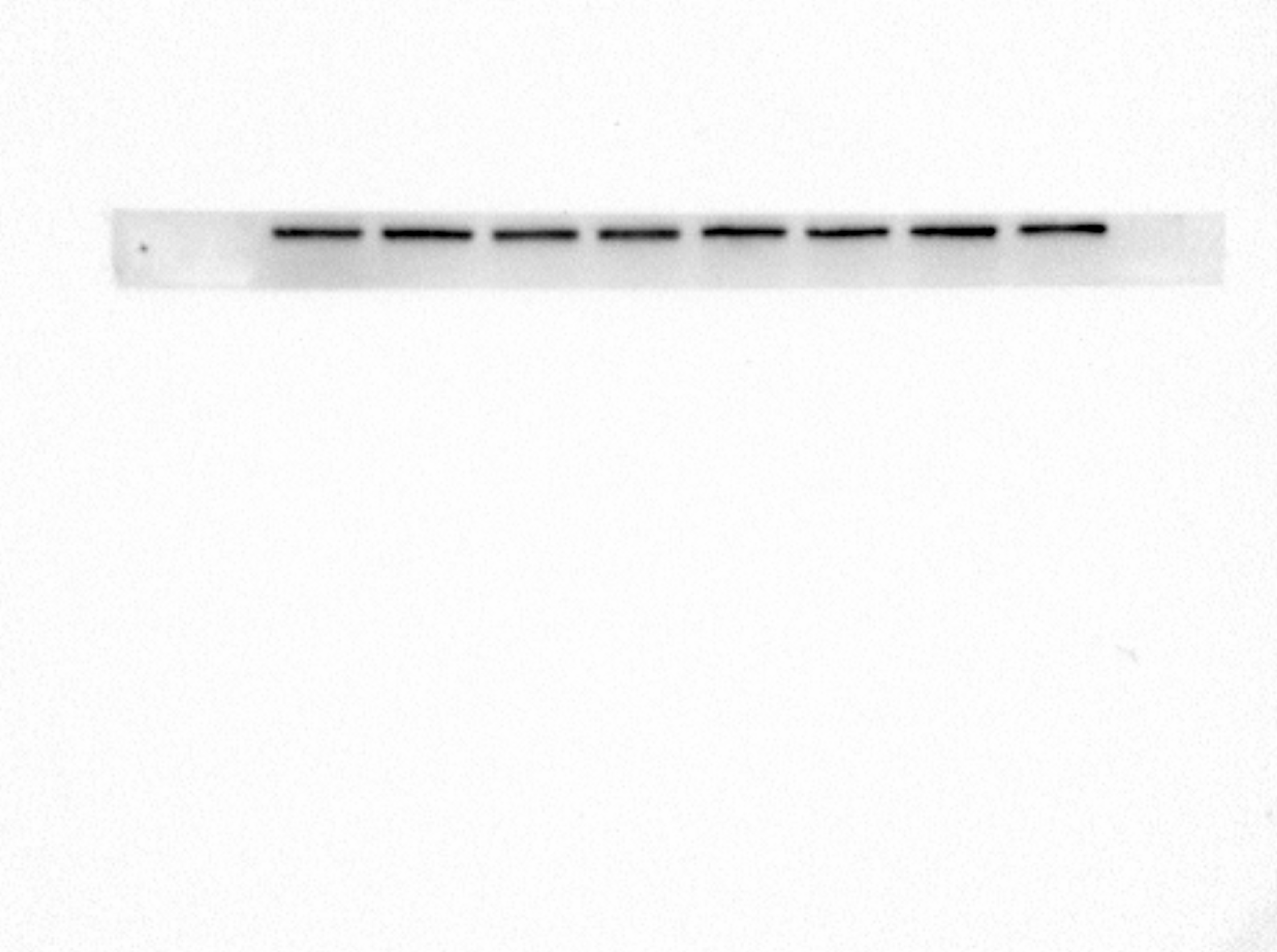

Supplement: Figure 5—figure supplement 1—source data 1. [file elife-83083-fig5-figsupp1-data1.zip › Figure 5-figure supplement 1-source data/Figure 5-Figure supplement 1A PKCa┴.tif]

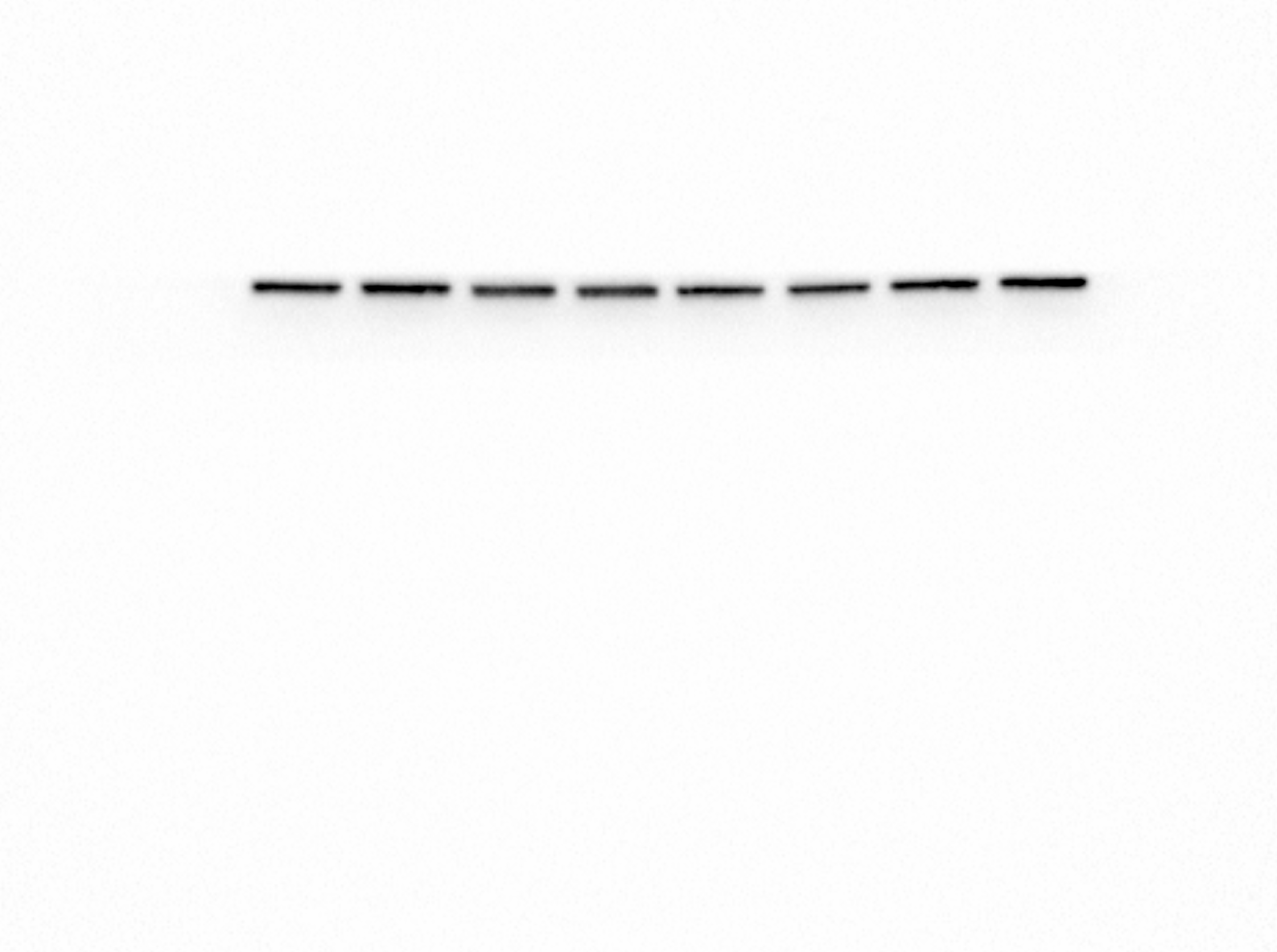

Supplement: Figure 5—figure supplement 1—source data 1. [file elife-83083-fig5-figsupp1-data1.zip › Figure 5-figure supplement 1-source data/Figure 5-Figure supplement 1A p-PKCa┴a┬.tif]

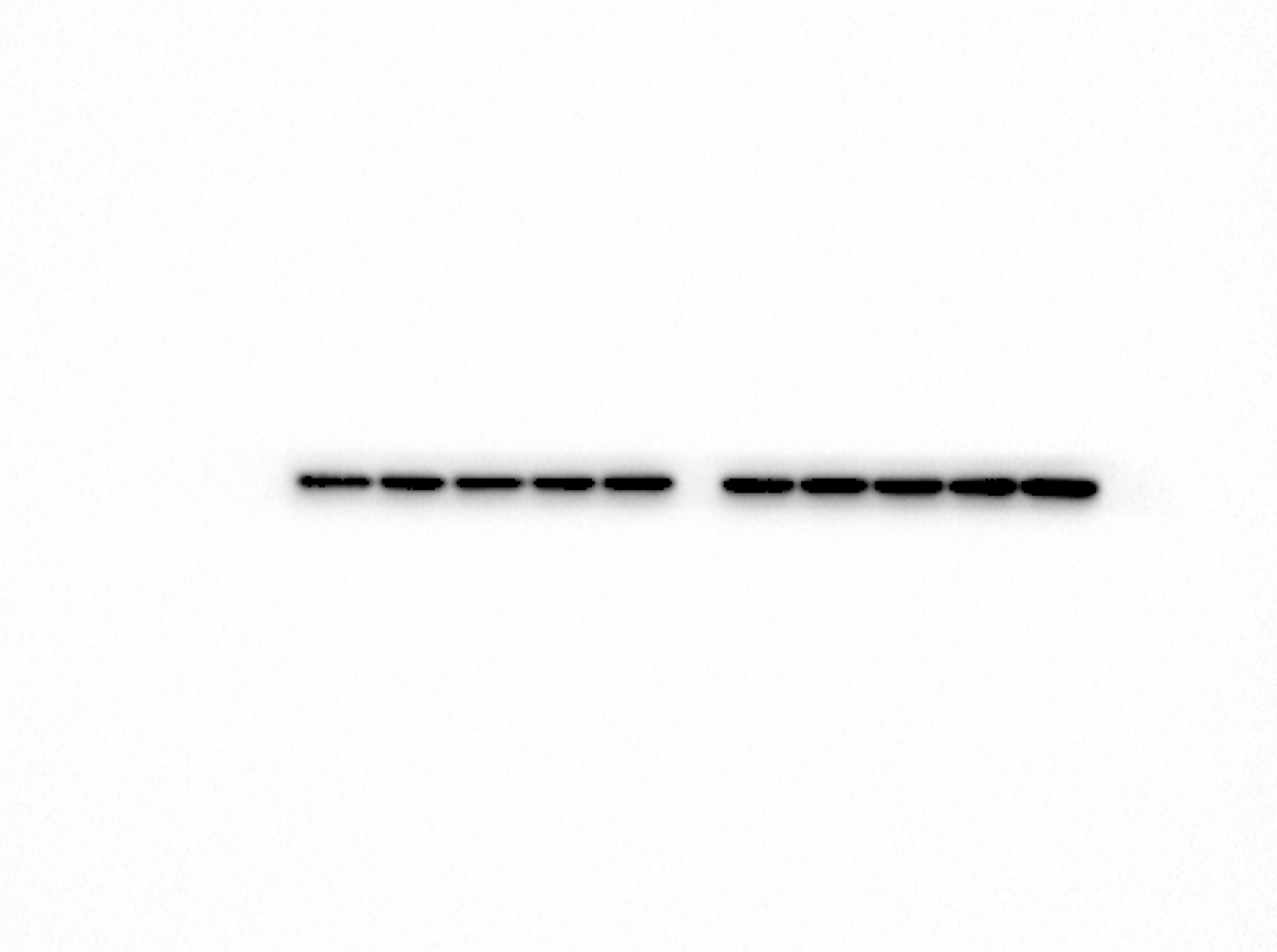

Supplement: Figure 5—figure supplement 1—source data 1. [file elife-83083-fig5-figsupp1-data1.zip › Figure 5-figure supplement 1-source data/Figure 5-Figure supplement 1B GAPDH.tif]

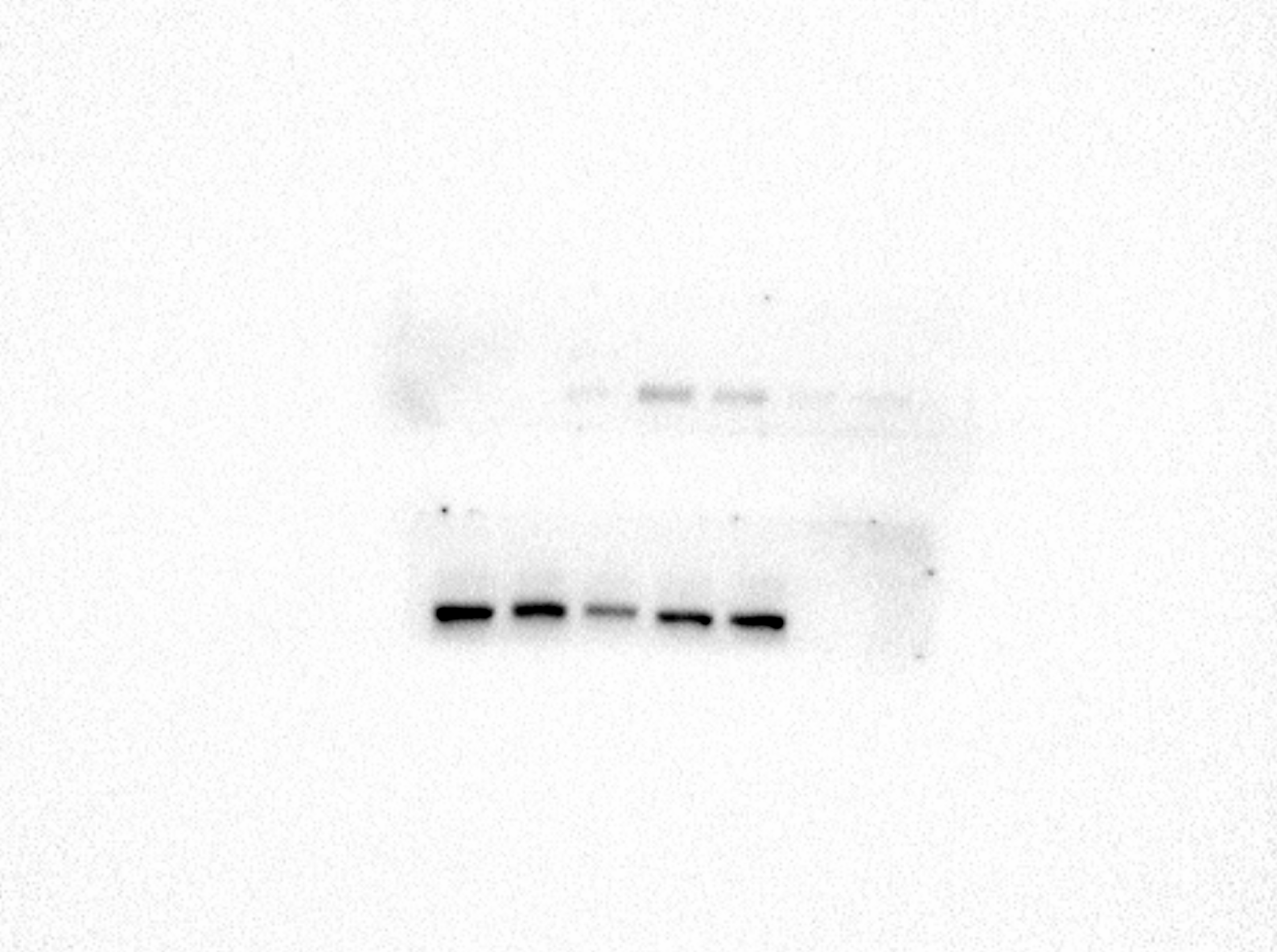

Supplement: Figure 5—figure supplement 1—source data 1. [file elife-83083-fig5-figsupp1-data1.zip › Figure 5-figure supplement 1-source data/Figure 5-Figure supplement 1B PKCPKDa╠.tif]

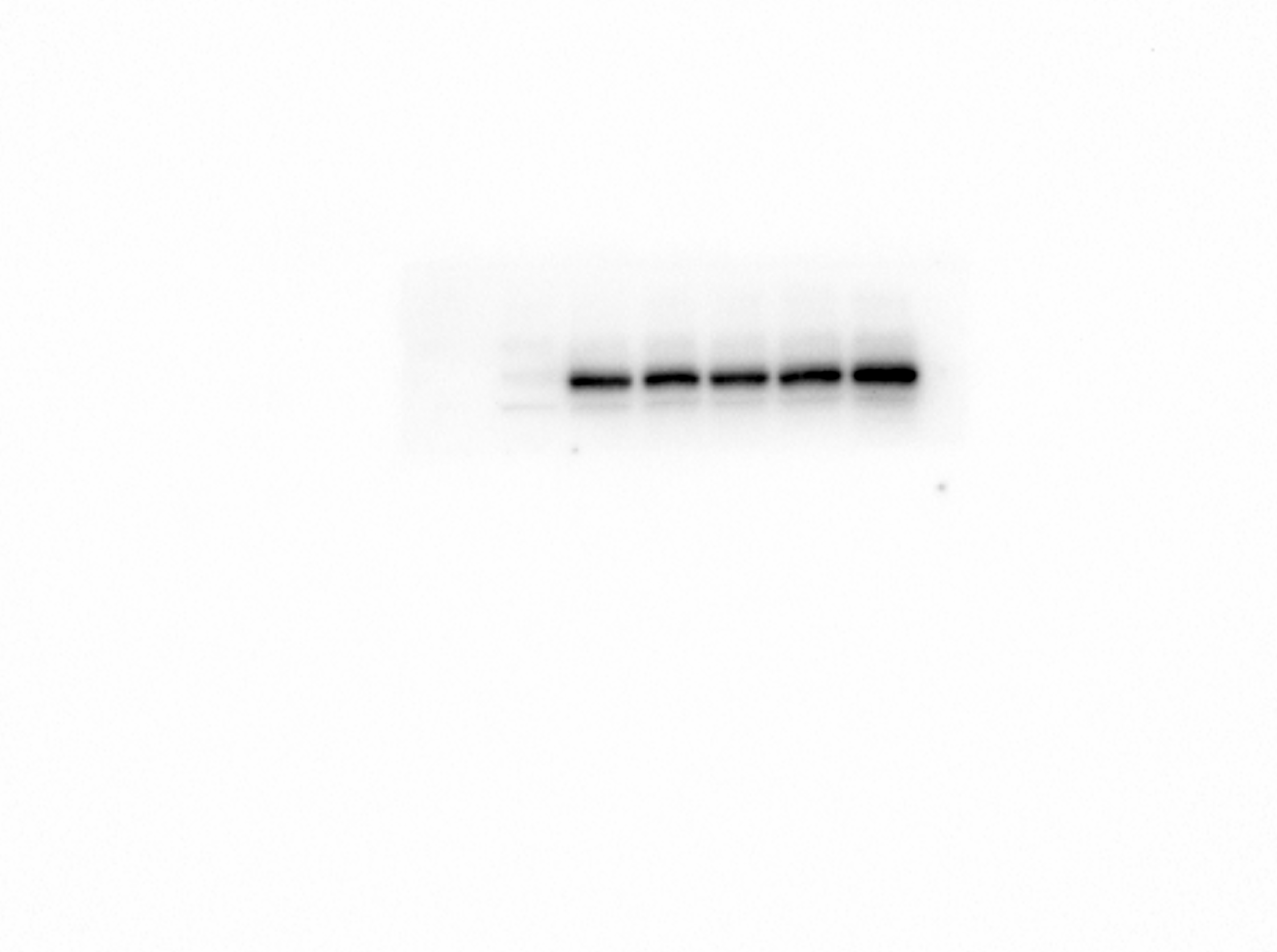

Supplement: Figure 5—figure supplement 1—source data 1. [file elife-83083-fig5-figsupp1-data1.zip › Figure 5-figure supplement 1-source data/Figure 5-Figure supplement 1B PKCa┴.tif]

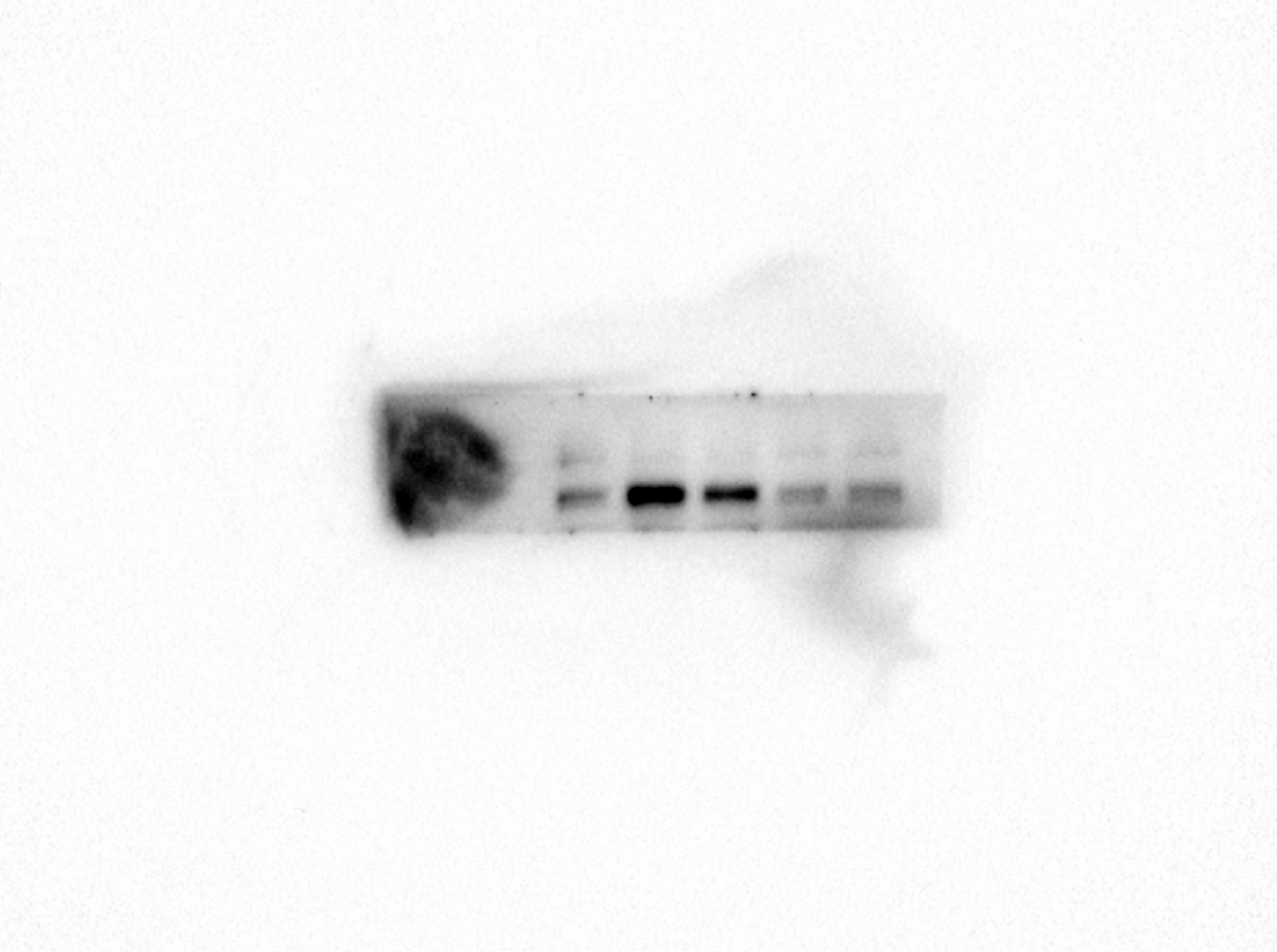

Supplement: Figure 5—figure supplement 1—source data 1. [file elife-83083-fig5-figsupp1-data1.zip › Figure 5-figure supplement 1-source data/Figure 5-Figure supplement 1B p-PKCPKDa╠.tif]

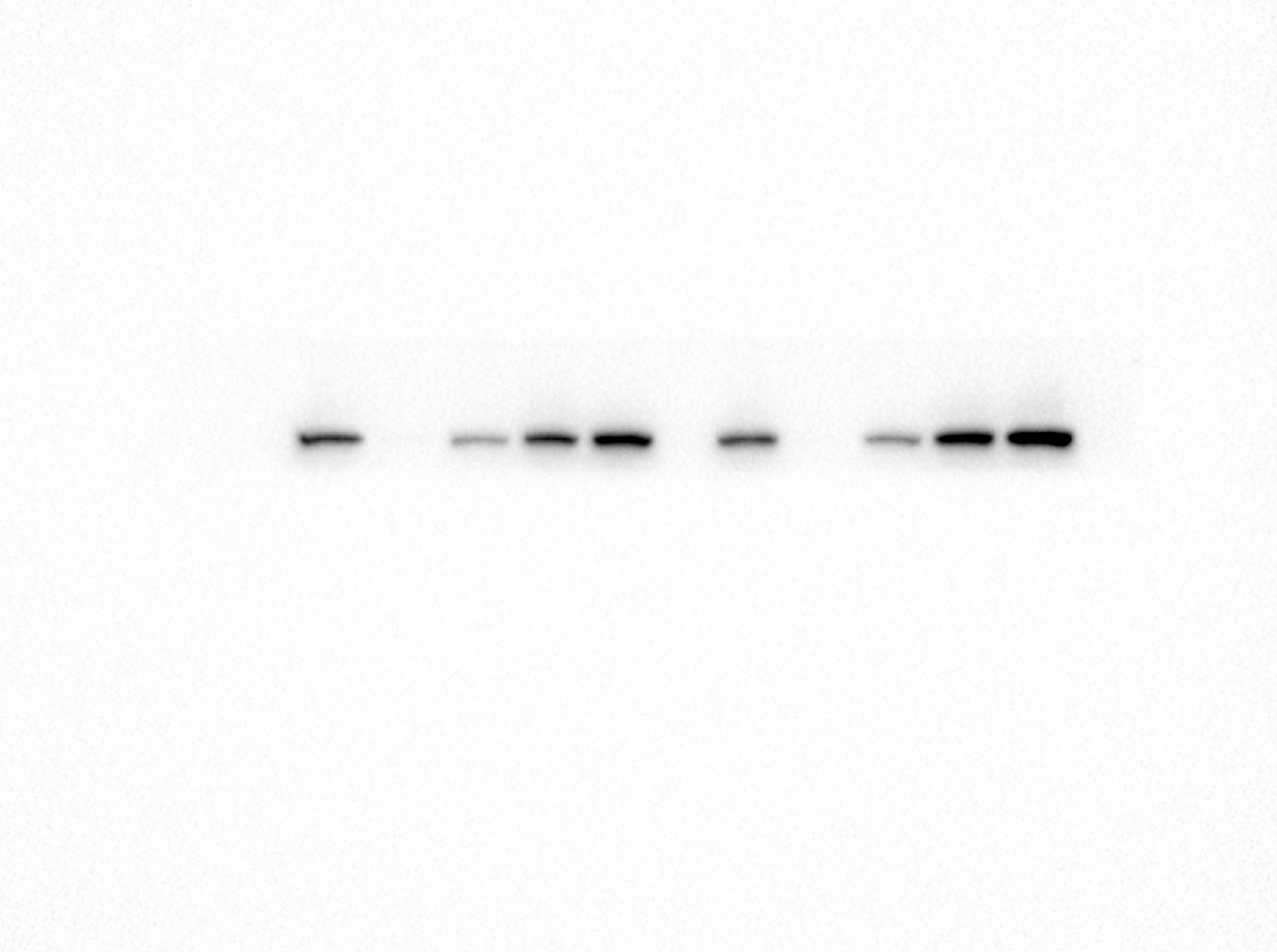

Supplement: Figure 5—figure supplement 1—source data 1. [file elife-83083-fig5-figsupp1-data1.zip › Figure 5-figure supplement 1-source data/Figure 5-Figure supplement 1B p-PKCa┴a┬.tif]

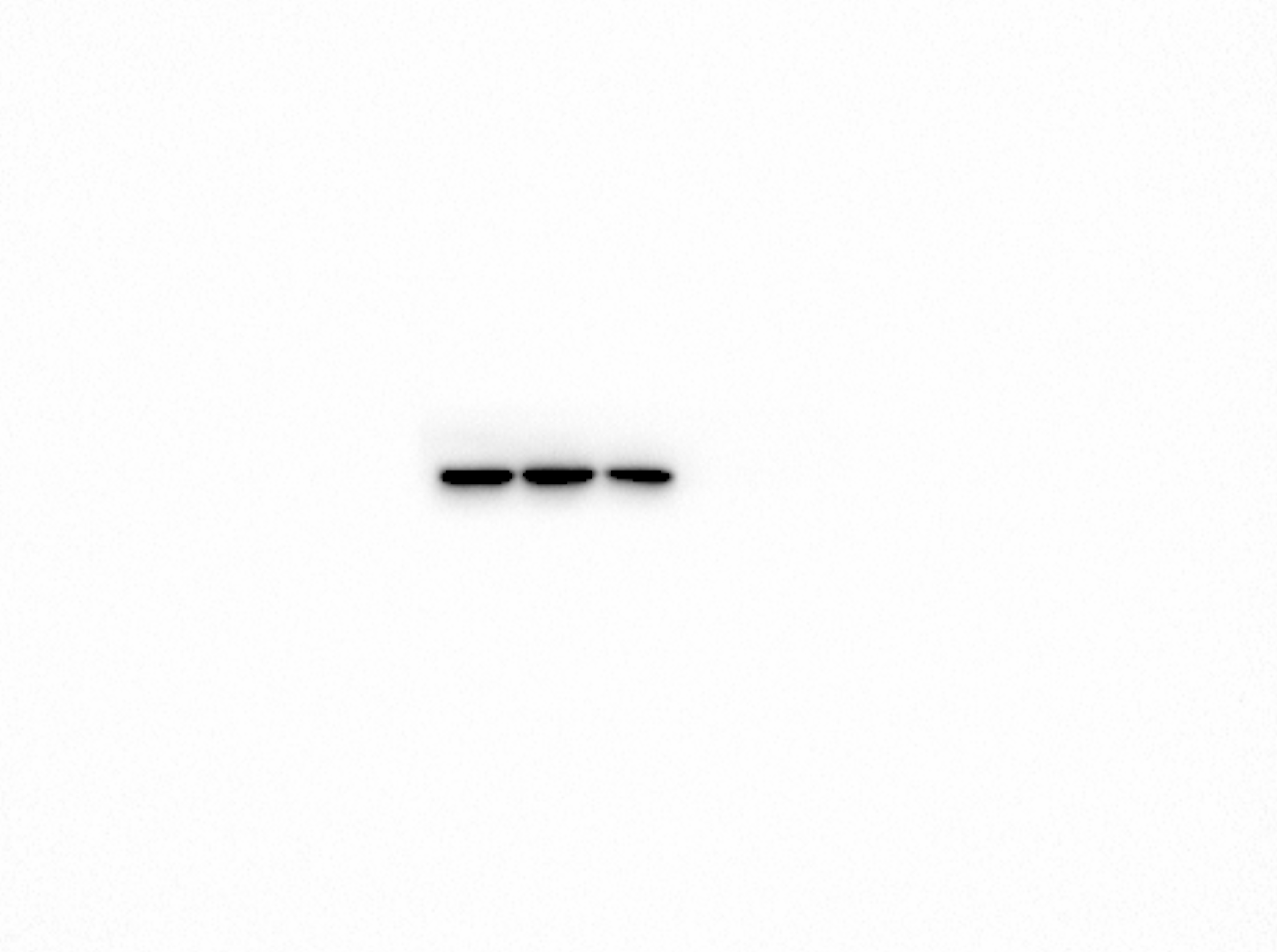

Supplement: Figure 5—figure supplement 1—source data 1. [file elife-83083-fig5-figsupp1-data1.zip › Figure 5-figure supplement 1-source data/Figure 5-Figure supplement 1C PKA C-a┴.tif]

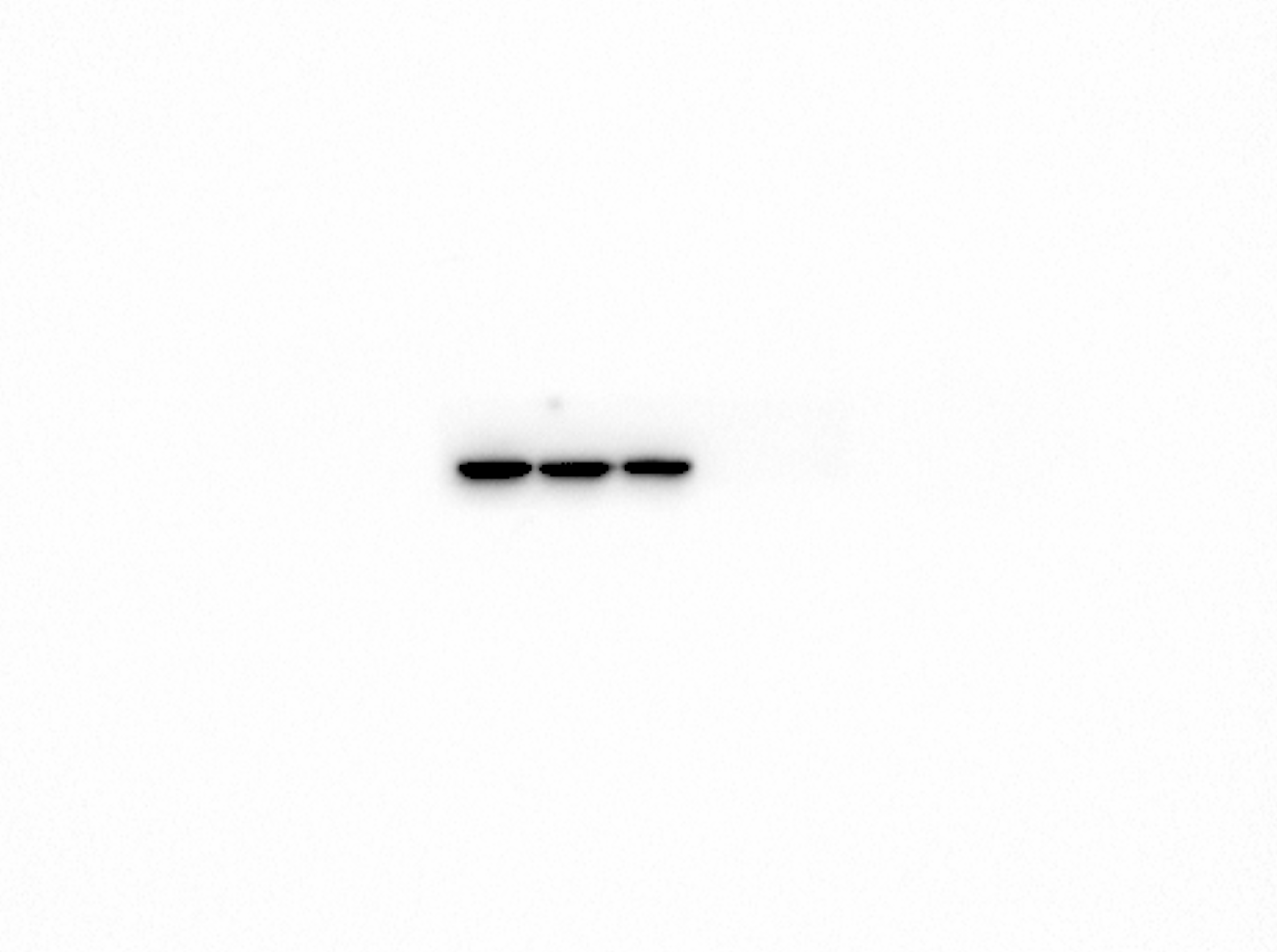

Supplement: Figure 5—figure supplement 1—source data 1. [file elife-83083-fig5-figsupp1-data1.zip › Figure 5-figure supplement 1-source data/Figure 5-Figure supplement 1C p-PKA C.tif]

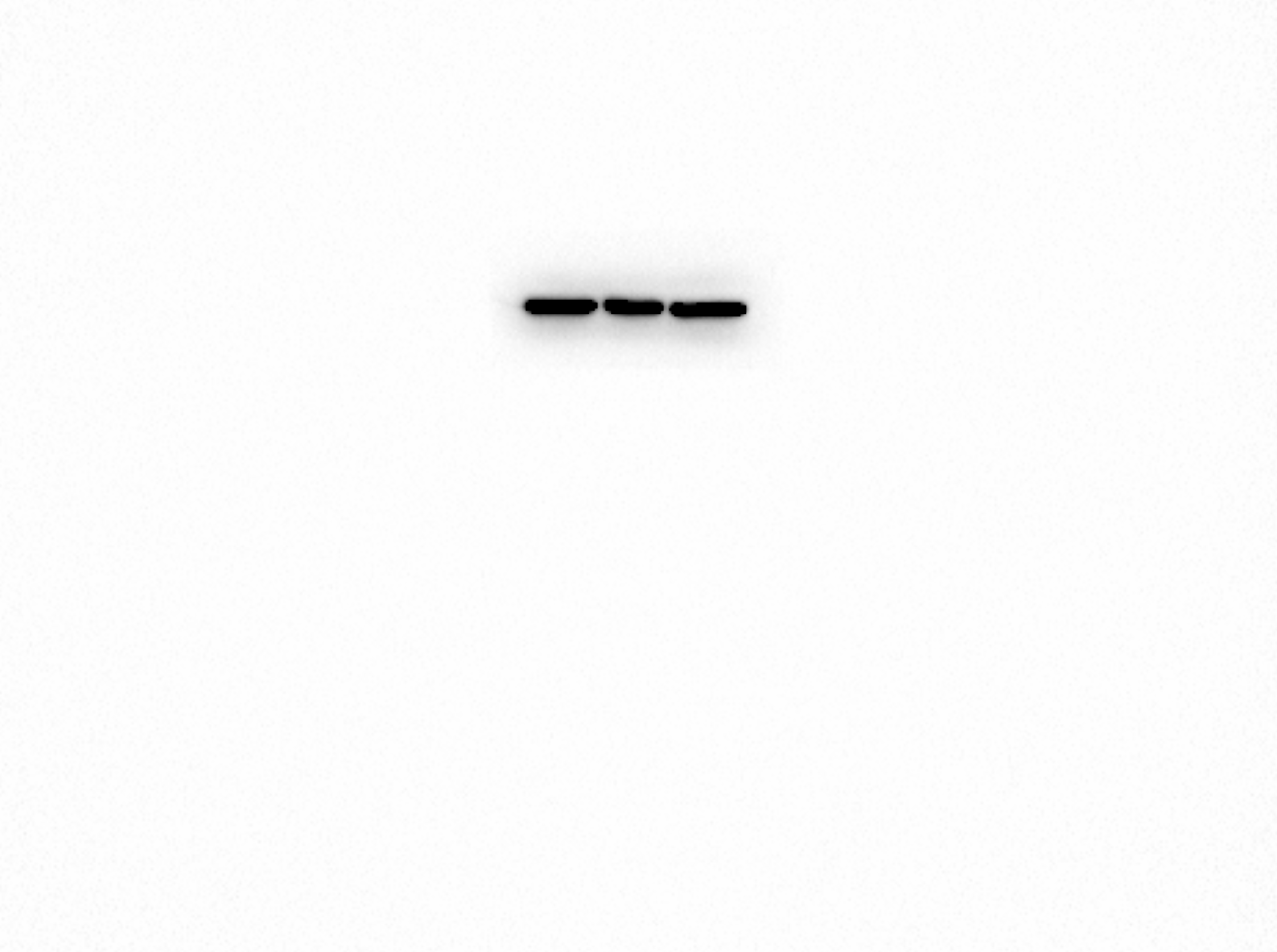

Supplement: Figure 5—figure supplement 1—source data 1. [file elife-83083-fig5-figsupp1-data1.zip › Figure 5-figure supplement 1-source data/Figure 5-Figure supplement 1C a┬-Tubulin.tif]

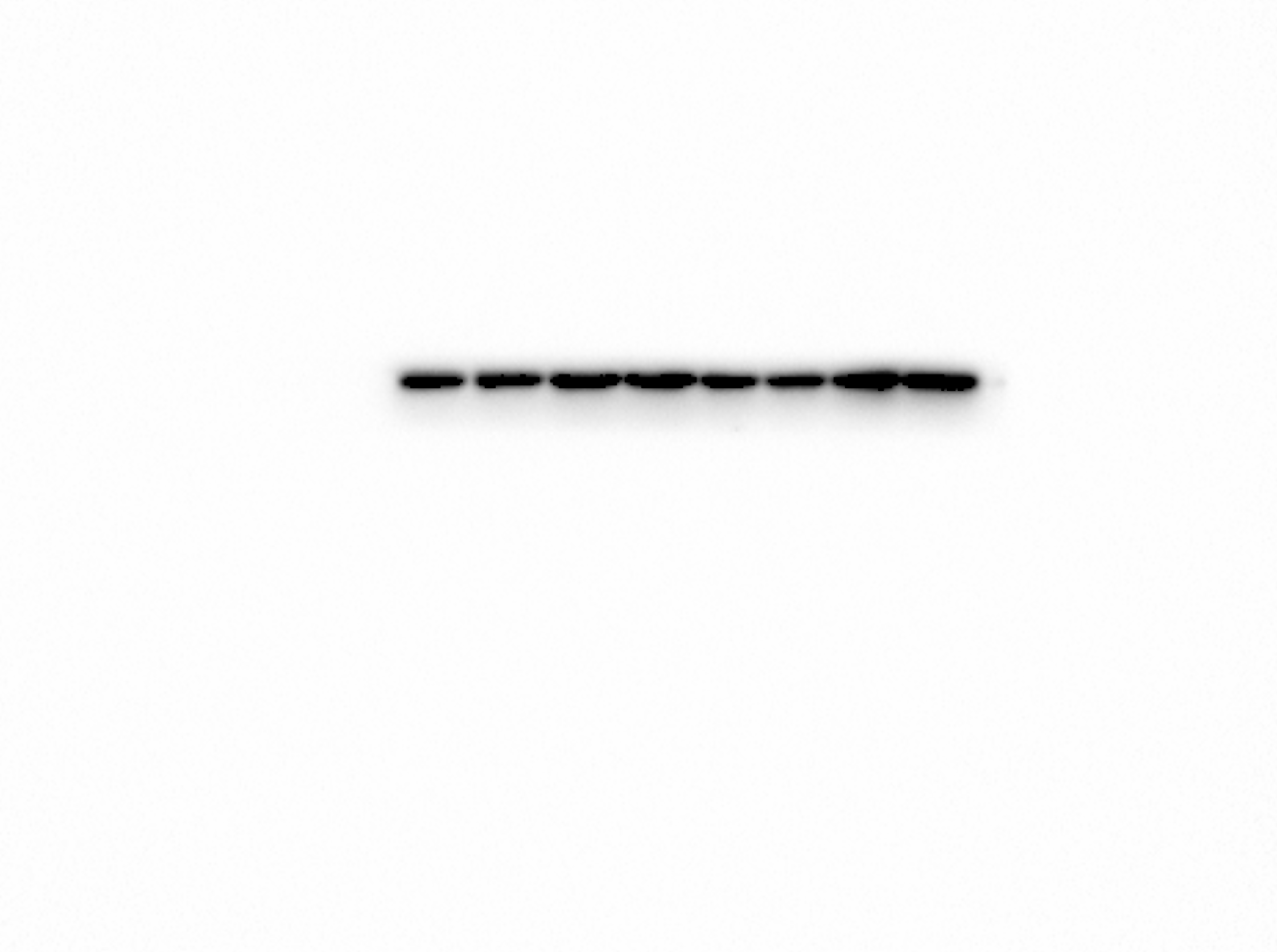

Supplement: Figure 5—figure supplement 1—source data 1. [file elife-83083-fig5-figsupp1-data1.zip › Figure 5-figure supplement 1-source data/Figure 5-Figure supplement 1D GAPDH.tif]

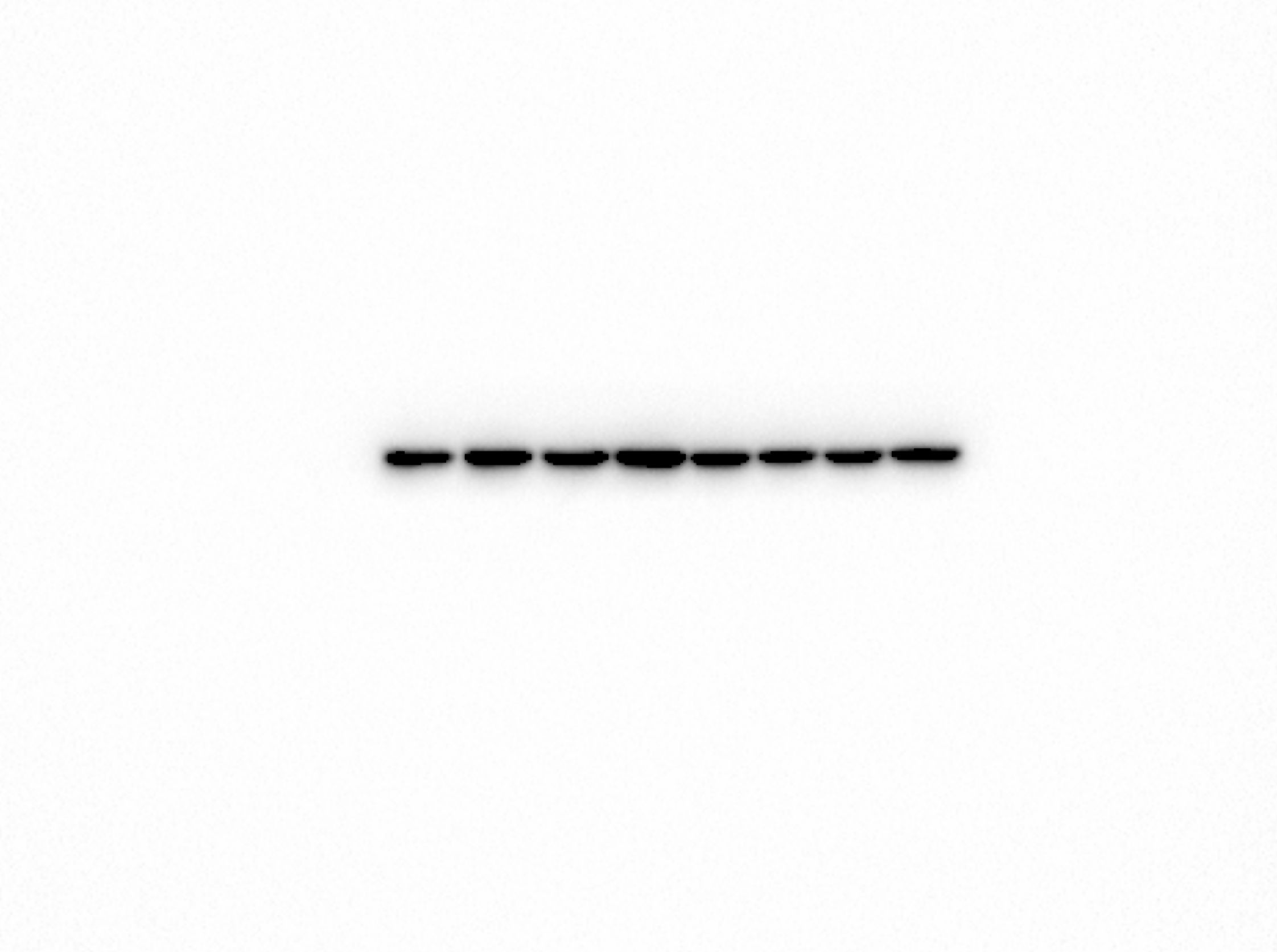

Supplement: Figure 5—figure supplement 1—source data 1. [file elife-83083-fig5-figsupp1-data1.zip › Figure 5-figure supplement 1-source data/Figure 5-Figure supplement 1D PKA C-a┴.tif]

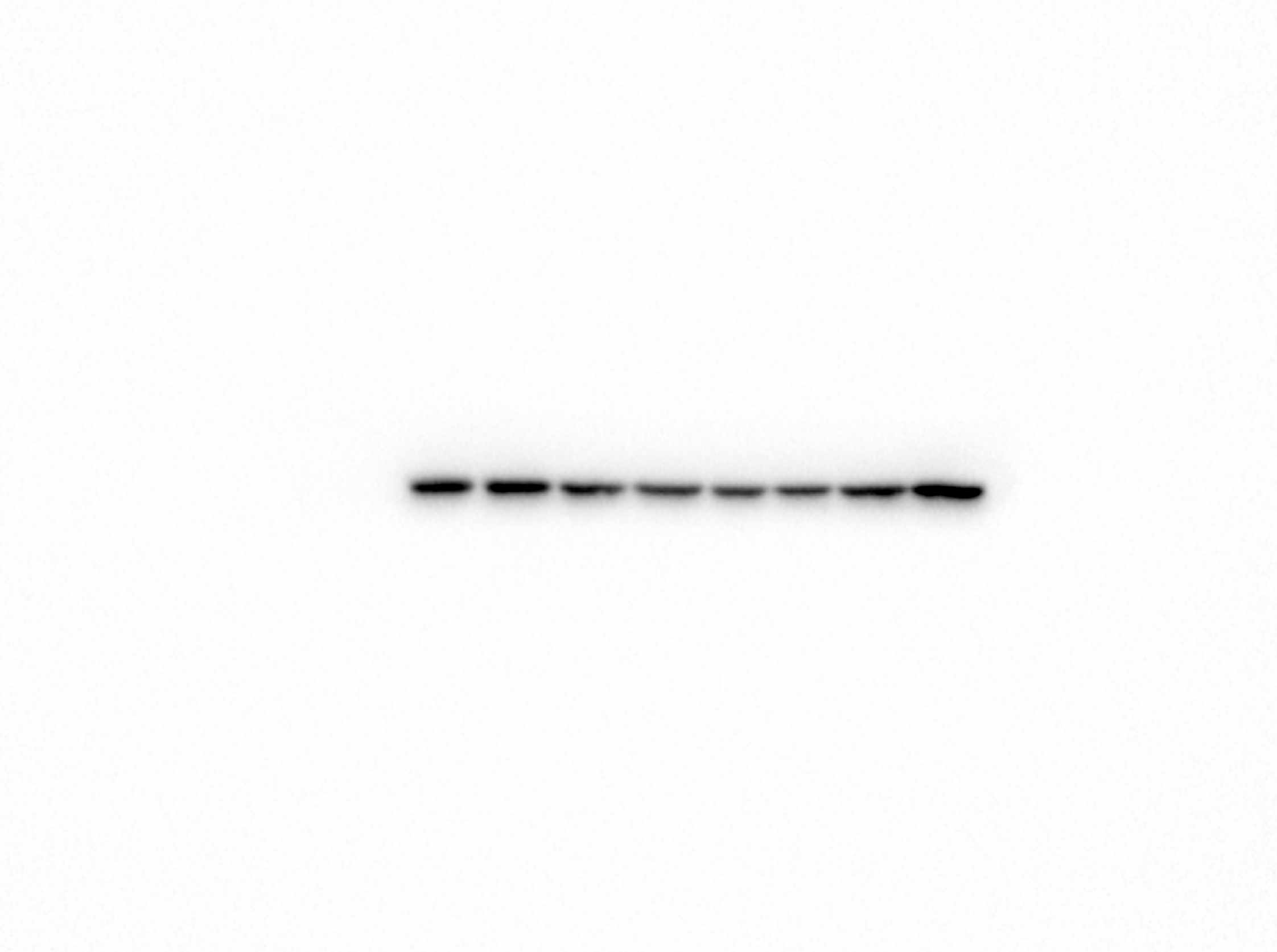

Supplement: Figure 5—figure supplement 1—source data 1. [file elife-83083-fig5-figsupp1-data1.zip › Figure 5-figure supplement 1-source data/Figure 5-Figure supplement 1D p-PKA C.tif]

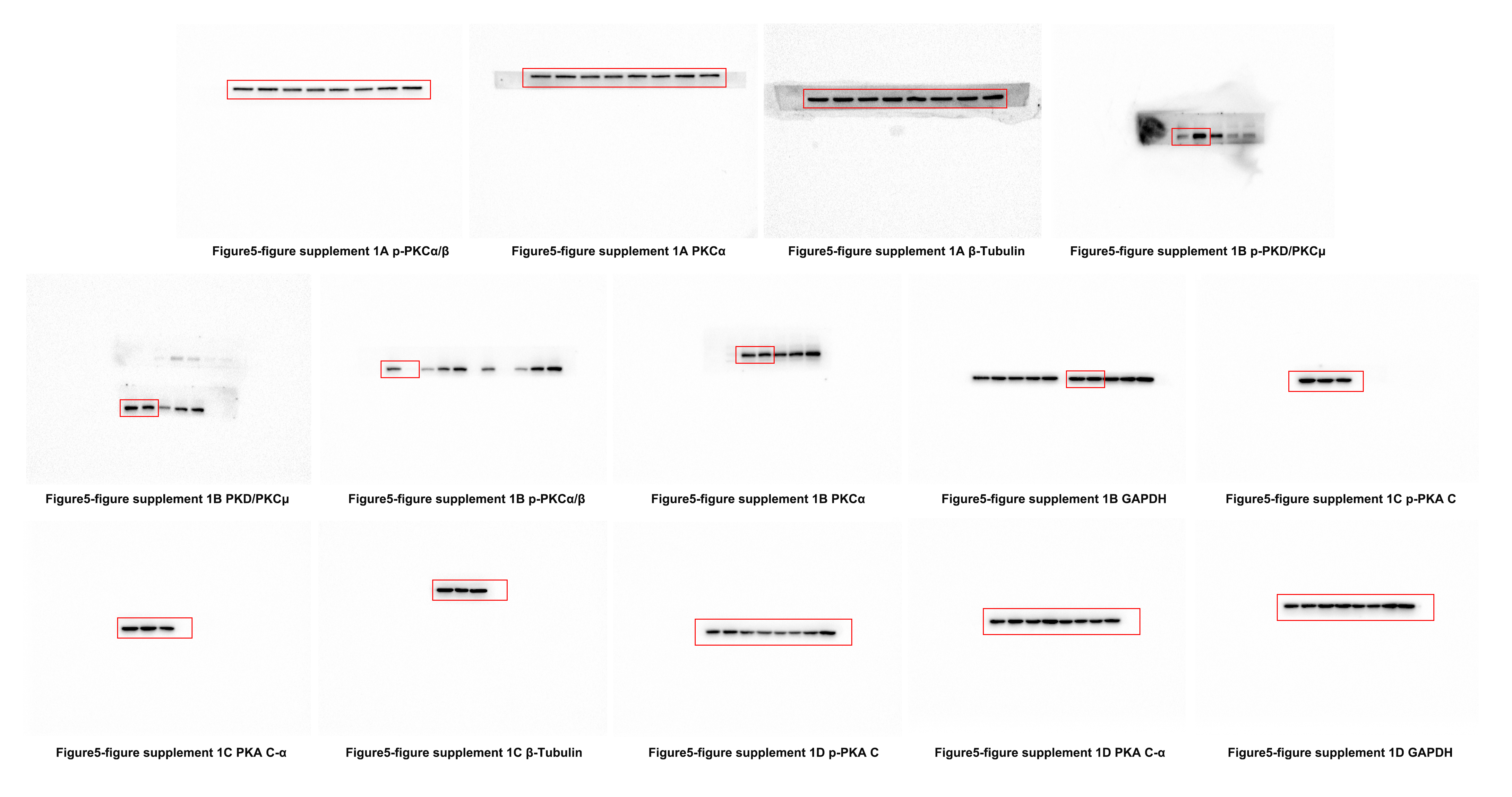

Supplement: Figure 5—figure supplement 1—source data 1. [file elife-83083-fig5-figsupp1-data1.zip › Figure 5-figure supplement 1-source data/Figure 5-figure supplement 1-source data.jpg]
