# Supplementary material for: Gαq-PKD/PKCμ signal regulating the nuclear export of HDAC5 to induce the IκB expression and limit the NF-κB-mediated inflammatory response essential for early pregnancy
Source: eLife. 2023 Jul 27;12:e83083. doi: 10.7554/eLife.83083 (PMC10374280; doi:10.7554/eLife.83083)
Supplement: Figure 6—source data 1. [file elife-83083-fig6-data1.doc]

**Statistical analyses for the role of stromal Gaq for blastocyst hatching and adhesion in the co-culture assay**

| Group | CON | GNAQ-KO | X^2^ | *P* value |
| --- | --- | --- | --- | --- |
| No. Blastocysts | 27 | 27 | - | - |
| No. Hatching blastocyst for co-culture 8h (%) ^a^ | 6(22.2) | 1(3.7) | 2.626 | 0.105 |
| No. Hatching blastocyst for co-culture 16h (%) ^b^ | 13(48.1) | 12(44.4) | 0.074 | 0.785 |
| No. Hatching blastocyst for co-culture 24h (%) ^b^ | 16(59.3) | 17(63.0) | 0.078 | 0.780 |
| No. Adhesion blastocyst for co-culture 72h (%) ^b^ | 8(29.6) | 3(11.1) | 2.854 | 0.091 |
|  |  |  |  |  |

NO., Number. a, Continuity correction chi-square test. b, Chi-square test.
