# Supplementary material for: Gαq-PKD/PKCμ signal regulating the nuclear export of HDAC5 to induce the IκB expression and limit the NF-κB-mediated inflammatory response essential for early pregnancy
Source: eLife. 2023 Jul 27;12:e83083. doi: 10.7554/eLife.83083 (PMC10374280; doi:10.7554/eLife.83083)
Supplement: Supplementary file 1. [file elife-83083-supp1.docx]

**Supplementary Materials:**

| **Supplementary file 1A**  **Characteristics of patients.** | | | | | | |
| --- | --- | --- | --- | --- | --- | --- |
|  | **Age**  **(Years)** | **G/P** | **Ethnicity** | **Gestational weeks** | **Days after ovalution** | **Embryo chromosome test** |
| **RPL** | 31 | 4/1 | HAN/CHINESE | 7+3 | 38 | NEG |
|  | 25 | 2/0 | HAN/CHINESE | 6+4 | 37 | NEG |
|  | 26 | 3/0 | HAN/CHINESE | 8+6 | 30 | NEG |
|  | 35 | 5/1 | HAN/CHINESE | 8+2 | 37 | NEG |
|  | 27 | 2/0 | HAN/CHINESE | 9 | 30 | NEG |
|  | 32 | 4/0 | HAN/CHINESE | 7+1 | 32 | NEG |
|  | 31 | 3/0 | HAN/CHINESE | 6+6 | 33 | NEG |
|  | 34 | 5/1 | HAN/CHINESE | 7+2 | 45 | NEG |
|  | 25 | 3/0 | HAN/CHINESE | 6+3 | 31 | NEG |
|  | 30 | 3/0 | HAN/CHINESE | 6+5 | 32 | NEG |
|  | 28 | 3/0 | HAN/CHINESE | 5+4 | 27 | NEG |
|  | 35 | 5/2 | HAN/CHINESE | 7+3 | 44 | NEG |
|  | 27 | 3/0 | HAN/CHINESE | 6+4 | 37 | NEG |
|  |  |  |  |  |  |  |
| **NOR** | 31 | 3/0 | HAN/CHINESE | 6+5 | 33 | NEG |
|  | 35 | 2/1 | HAN/CHINESE | 7+3 | 35 | NEG |
|  | 26 | 2/1 | HAN/CHINESE | 8+2 | 47 | NEG |
|  | 29 | 4/2 | HAN/CHINESE | 8+2 | 41 | NEG |
|  | 29 | 4/0 | HAN/CHINESE | 8+5 | 45 | NEG |
|  | 27 | 3/0 | HAN/CHINESE | 6+3 | 27 | NEG |
|  | 28 | 3/0 | HAN/CHINESE | 7+2 | 37 | NEG |
|  | 35 | 4/2 | HAN/CHINESE | 8+4 | 47 | NEG |
|  | 32 | 2/0 | HAN/CHINESE | 6+6 | 34 | NEG |
|  | 30 | 1/0 | HAN/CHINESE | 7+3 | 36 | NEG |
|  | 24 | 3/0 | HAN/CHINESE | 7+1 | 37 | NEG |
|  | 29 | 2/1 | HAN/CHINESE | 6+6 | 32 | NEG |
|  | 25 | 2/0 | HAN/CHINESE | 6+2 | 31 | NEG |

RPL, recurrent pregnancy loss. NOR, normal pregnancy. G/P, time of gravida / time of para. NEG, negative.

| **Supplementary file 1B**  **Inhibitors or activators used in the study.** | | | | |
| --- | --- | --- | --- | --- |
| **Kinase** | **Inhibitor or activator** | **Company** | **Catalog number** | **Working concentration** |
| PKC | Phorbol 12-myristate 13-acetate (PMA) | MCE | HY-18739 | 2 nM |
| NFκB | pyrrolidinedithiocarbamate ammonium (PDTC) | MCE | HY-18738 | 10 uM |
| PKD/PKCμ | CID755673 | MCE | HY-12239 | 50 uM |
| HDAC5 | LMK235 | MCE | HY-18998 | 2 uM |

| **Supplementary file 1**C  **Primer sequences (all 5’ to 3’) used in quantitative RT-PCR analysis of target genes.**  **qRT-PCR primer** | | | | | | |
| --- | --- | --- | --- | --- | --- | --- |
| Abbrevia-tion | Gene | | Forward primer | | | Reverse primer |
| GNAQ | Guanine nucleotide-binding protein G(q) subunit alpha | | GTTGATGTGGAAG AAGGTGTCTA | | | GTAGGCAGGTAGGCAGGGT |
| PRL | Prolactin | | CTACATCCATAACCTCTCCTCAG | | | GGGCTTGCTCCTTGTCTTC |
| IGFBP-1 | Insulin-like growth factor binding protein-1 | | AGAGTCGTAGAGAGTTTAGC | | | ACACTGTCTGCTGTGATAA |
| IL-11 | Interleukin-11 | | CTCATCTTCTTCACCATT | | | ACTTCCTACTTCCTACAT |
| IL-6 | Interleukin-6 | | CTTTTGGAGTTTGAGGTATACCTAG | | | CGCAGAATGAGATGAGTTGTC |
| IL-1β | Interleukin-1 beta | | ATGGCTTATTACAGTGGCA | | | GTAGTGGTGGTCGGAGATT |
| IL-1R 1 | Interleukin-1 receptor 1 | | CTTCCTCTCCAGCCTTCT | | | GGACCGTTATTGACCTGAA |
| IL-1R 2 | Interleukin-1 receptor 2 | | GAGAAGAAGAGACACGGATG | | | CAGGACACAGCGGTAATAG |
| IL-11 | Interleukin-11 receptor | | GACGAAGCGAGTGGAT | | | CCTTTGACCTGGAGACA |
| NFKBIA | NF-kappa-B inhibitor alpha | | AACATGGACTTGTATATTTG | | | CTCAGAATTTCAATGATCTT |
| GAPDH | Glyceraldehyde-3-phosphate dehydrogenase | | ATTTGGCTACAGCAACAGG | | | TTGAGCACAGGGTACTTTATT |
| **ChIP RT–qPCR primer** | | | | | | |
| Abbrevia-tion | Gene | | | Forward primer | | Reverse primer |
| NFKBIA | NF-kappa-B inhibitor alpha | | | AAAAAACGGAAAGGACCG | | TGGCTTCGTCCTCTGCTA |
| **Vector sequence** | | | | | | |
| Gene | | Forward primer | | | Reverse primer | |
| GANQ CDS | | ACTCTGGAGTCCATCATGGCG | | | AGCGTAATCTGGAACATCGTATGGGTA (HA)  GACCAGATTGTACTCCTTCAGGTTCAAC | |
| NFKBIA CDS | | ATGTTCCAGGCGGCCGAGCG | | | GCGGCCGCTTAAGCGTAATCTGGAACATCGTATGGGTATAACGTCAGACGCTGGC | |
